# Developing Photoaffinity Probes for Dopamine Receptor D_2_ to Determine Targets of Parkinson’s Disease Drugs

**DOI:** 10.1021/acschemneuro.2c00544

**Published:** 2022-10-02

**Authors:** Spencer T. Kim, Emma J. Doukmak, Raymond G. Flax, Dylan J. Gray, Victoria N. Zirimu, Ebbing de Jong, Rachel C. Steinhardt

**Affiliations:** † Department of Chemistry, Syracuse University, Syracuse, New York 13244, United States; ‡ BioInspired Institute, Syracuse University, Syracuse, New York 13244, United States; § SUNY Upstate Medical University, Syracuse, New York 13244, United States; ∥ Department of Biomedical and Chemical Engineering, Syracuse University, Syracuse, New York 13244, United States

**Keywords:** dopamine receptors, photo-cross-linking, photoaffinity labeling (PAL), proteomics, bioinformatics, endocannabinoid pathway, GABA receptor, muscarinic receptor M1, pramipexole, ropinirole, DRD2

## Abstract

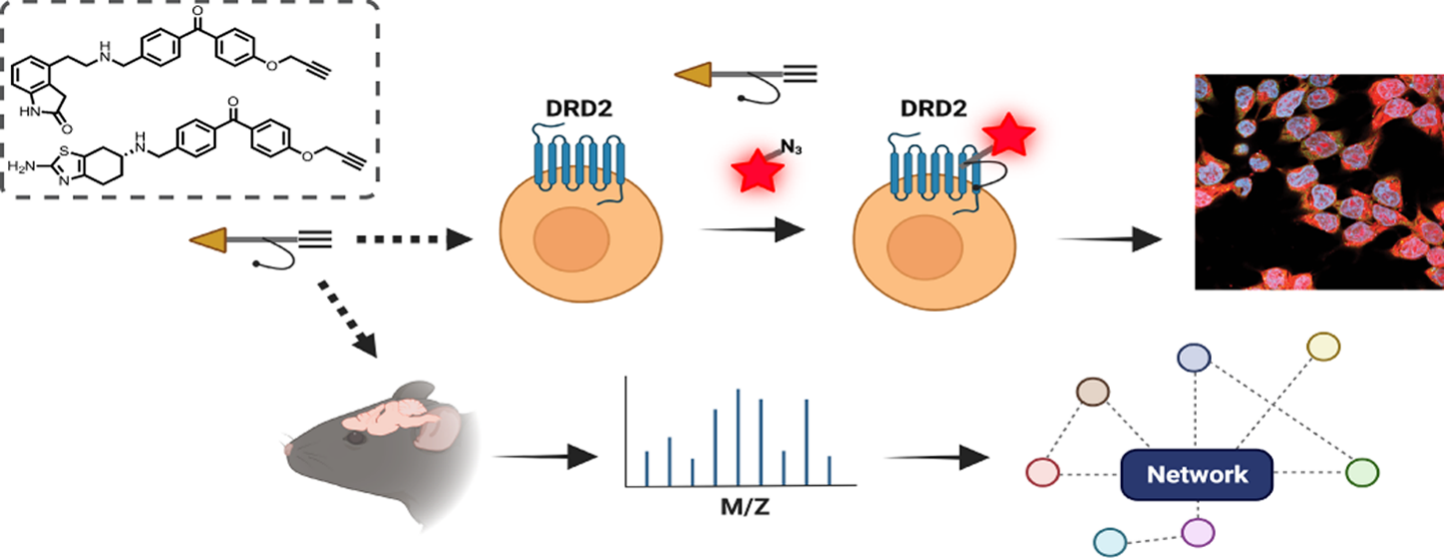

Dopaminergic pathways control highly consequential aspects of physiology and behavior. One of the most therapeutically important and best-studied receptors in these pathways is dopamine receptor D_2_ (DRD2). Unfortunately, DRD2 is challenging to study with traditional molecular biological techniques, and most drugs designed to target DRD2 are ligands for many other receptors. Here, we developed probes able to both covalently bind to DRD2 using photoaffinity labeling and provide a chemical handle for detection or affinity purification. These probes behaved like good DRD2 agonists in traditional biochemical assays and were able to perform in chemical–biological assays of cell and receptor labeling. Rat whole brain labeling and affinity enrichment using the probes permitted proteomic analysis of the probes’ interacting proteins. Bioinformatic study of the hits revealed that the probes bound noncanonically targeted proteins in Parkinson’s disease network as well as the retrograde endocannabinoid signaling, neuronal nitric oxide synthase, muscarinic acetylcholine receptor M1, GABA receptor, and dopamine receptor D_1_ (DRD1) signaling networks. Follow-up analysis may yield insights into how this pathway relates specifically to Parkinson’s disease symptoms or provide new targets for treatments. This work reinforces the notion that the combination of chemical biology and omics-based approaches provides a broad picture of a molecule’s “interactome” and may also give insight into the pleiotropy of effects observed for a drug or perhaps indicate new applications.

## 1 Introduction

Physiological states ranging from euphoria to psychosis are governed by the neuroanatomical pathways of the dopaminergic nervous system.^1^ The dopaminergic neurons comprising this system function via binding of the neurotransmitter dopamine to its receptors. There are a handful of subtypes of dopamine receptors expressed by these neurons that control diverse aspects of behavior, and it is hypothesized that individual subtypes combine and contribute to different biochemical pathways.^2,3^ Unfortunately, though, it is extremely difficult to selectively target individual dopamine receptor subtypes, let alone pathways, with drugs or other nonendogenous stimuli.^1^ From the standpoint of directing neurochemistry via small molecules, the wide variety of physiological responses controlled by the dopaminergic system—coupled with the lack of selective drugs—makes drug/probe development highly challenging.

There are canonically five subtypes of dopamine receptors, D_1–5_, which are separated into two families: D_1_-like (D_1_ and D_5_) and D_2_-like (D_2–4_), with receptors D_1_ and D_2_ exhibiting the highest expression density of all dopamine receptors in the human central nervous system.^4^ Further, there are several isoforms of the individual receptor subtypes.^5^ Perhaps, the best-studied and most medically important dopamine receptor is D_2_ (DRD2), which is the focus of therapeutic intervention for diseases such as psychosis and Parkinson’s.^6^ In fact, dopamine receptors 1, 3, and 4 are also bound/blocked to some degree by drugs targeting DRD2, but it is unclear how much the pharmacodynamics of these subtypes contribute to the drug’s clinical effectiveness.^1^

This poor selectivity of available drugs is likely due in large part to the lack of structural data regarding DRD2, coupled with the high structural homology between receptor subtypes.^7−9^ Only a handful of structures exist, and those that do rely on extensive mutation to enable easier isolation and temperature stability—even to the point of altering the receptor’s ligand binding.^10,11^ It is hypothesized that DRD2 does not have a “rigid” orthosteric site, further complicating analysis.^11^

Combined photoaffinity labeling and proteomic analysis are powerful tools for showing the breadth of proteins bound by a drug, as well as the specific peptide sequence in the vicinity of the drug binding site.^12^ These works are enabled by the strategies used in activity-based protein profiling (ABPP), photoaffinity labeling, and advances in mass spectrometry.^13^ This strategy has recently been used to great effect to study the activity profiles of NSAIDs, cannabinoid drugs, and methamphetamine, for example.^14−16^ Detailed receptor binding site studies have been enabled by photo-cross-linking the CNS drugs granisetron, propofol, glutamate receptor modulators, and others.^17−19^ DRD2 itself has a long history of use with photoaffinity technology to aid the biochemical characterization of this hard-to-handle membrane-bound protein.^20,21^

Here, we have adapted these technologies to show both broad target engagement of probes based on DRD2-binding pharmacophores and the specific peptides in the local environment of the probe when bound to the DRD2 receptor. Together, these data may show potential targets of drugs based on similar scaffolds, as well as provide more insight into the functional structure of DRD2.

## 2 Results and Discussion

### 2.1 Design of Probes

The framework for our probe design was to employ a DRD2-binding pharmacophore attached to a photoreactive group and an alkyne for CuAAC chemistry. To fabricate novel ligands for DRD2, we required a pharmacophore to retain high affinity while tolerating the addition of (1) a photoaffinity group for covalent attachment to the receptor and (2) an alkyne as a handle for attachment of a fluorophore. For the photoaffinity moiety, two of the most common photo-cross-linking groups are benzophenones and diazirines, which upon irradiation with UV light generate ketyl radicals and carbenes, respectively.^22^ There are benefits and drawbacks to both groups; for example, benzophenone generates a longer-lived reactive intermediate and is relatively easy to synthesize but is hampered by its large size. In contrast, the carbene is highly reactive and shorter-lived—which can be advantageous—but is relatively harder to synthesize in high yield and may degrade quickly. Additionally, off-target/nonspecific binding proteomic profiles vary for probes incorporating the two different cross-linkers.^23−25^ We thus chose to assay the performance of both groups as cross-linking moieties in our probes and synthesized a panel of derivatives to assay, which focused on replacing the N-alkyl groups of ropinirole with photo-cross-linking groups. This allowed us to leave the ropinirole pharmacophore mostly intact while adding new functionality for photo-cross-linking and CuAAC chemistry.

**Figure 1:**
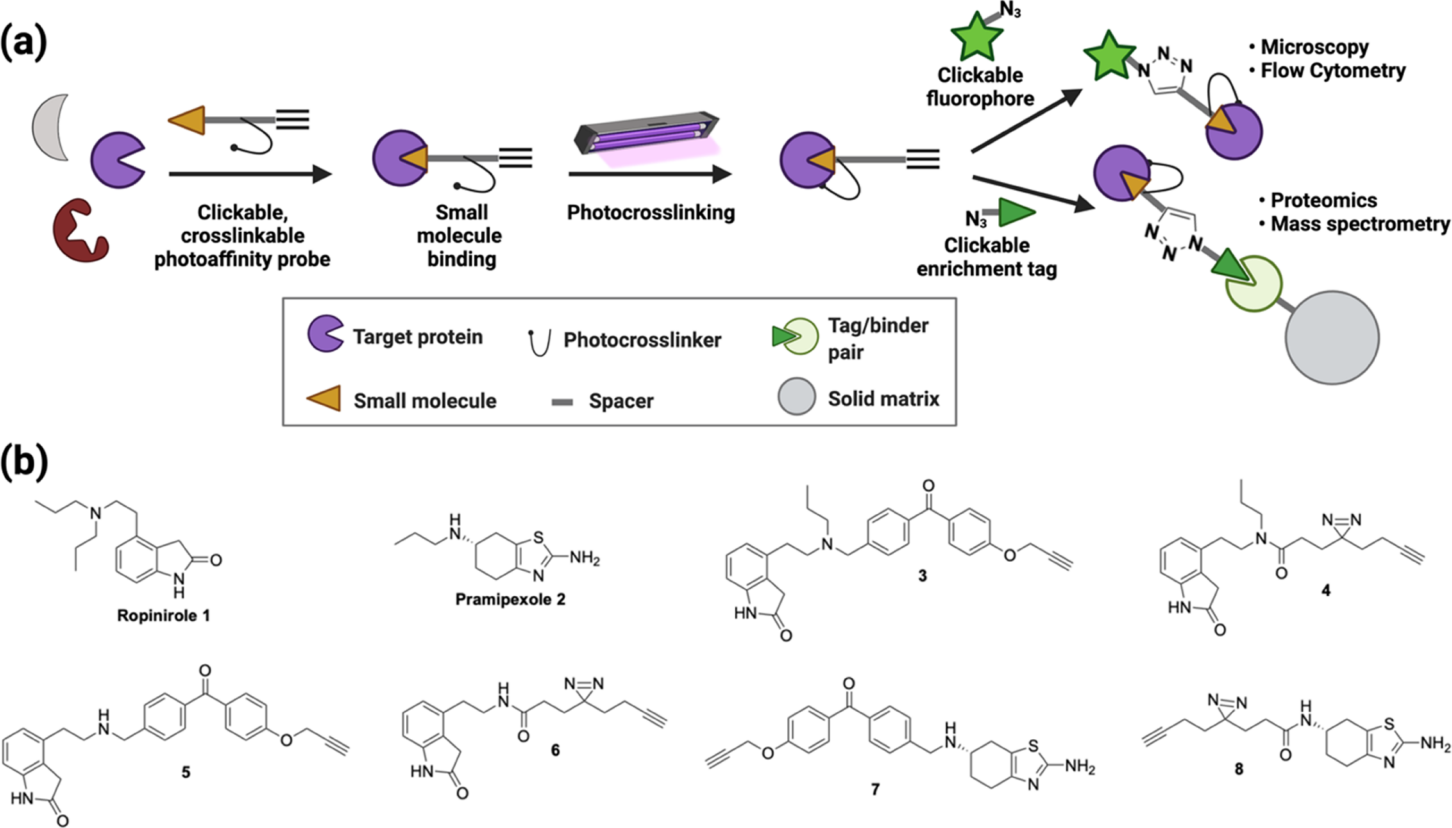
(a) Photoaffinity labeling for the simultaneous determination of protein targets and sites of probe labeling. Probes bear two handles: one for visualization and one for enrichment. This allows monitoring of probe–target interactions by imaging as well as enrichment for proteomics. (b) Ropinirole, pramipexole, and clickable, photo-cross-linkable target probes based on their pharmacophores.

**Figure 2:**
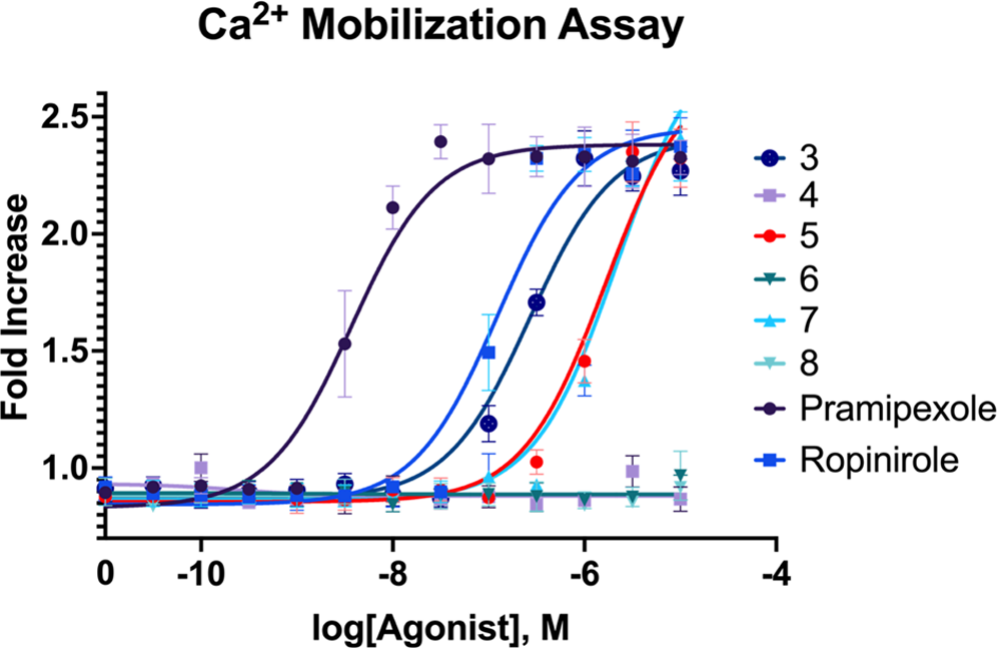
Intracellular calcium flux assay. HEK293T cell line stably expressing constructs for human DRD2 and a chimeric G-protein is loaded with calcium-sensing dye, Fura-4. After dosing the probe, confocal microscopy is used to determine the calcium flux in the cell by change in dye fluorescence. EC_50_ curves determined with GraphPad software using a Hill slope of 1.0.

We chose to build our probes based on the core structures of two highly prescribed DRD2 agonists: ropinirole and pramipexole (Figure 1b, **1** and **2**) (in 2018, they were ranked as the 147th and 187th most-prescribed drugs in the United States, although ropinirole’s manufacture has since been discontinued).^26,27^ The drugs treat the symptoms of Parkinson’s disease and other neurological maladies. However, both come with many serious adverse drug reactions, such as confusion, hallucinations, psychosis, excessive somnolence—which may persist even after discontinuing use—and tardive dyskinesia.^11,28^ Troublingly, as much as 74% of patients experience such adverse drug reactions in the case of pramipexole.^29^ The high demand for such treatments and the high adverse reaction rate to currently available drugs highlight the need for (1) a better understanding of how DRD2 ligands bind the receptor; (2) elucidation of the biophysics of how small molecules can direct DRD2 to various signaling pathways; and (3) the other off-target proteins such drugs engage. These three factors are critical for both effective DRD2 drugs and preventing off-target effects.

From a chemical perspective, these scaffolds represent two very distinct structures. Ropinirole is a substituted oxindole, while pramipexole is a cyclohexyl-thiazoline with a chiral center. The two molecules have different three-dimensional structures and surface areas, and H-bond donating and accepting potential. Their most critical similarity is that they are both substituted with an alkylamine. Through structure–activity studies and comparison with the native ligand, dopamine, it can be surmised that this basic nitrogen is critical to receptor binding.^30−36^ Conversely, the analysis of DRD2 ligands—as well as work on structurally related receptors—indicated that this basic nitrogen was a likely region to permit more steric bulk to be attached to a pharmacophore.^33,37,38^

### 2.2 Synthesis of Probes

**Scheme 1:**
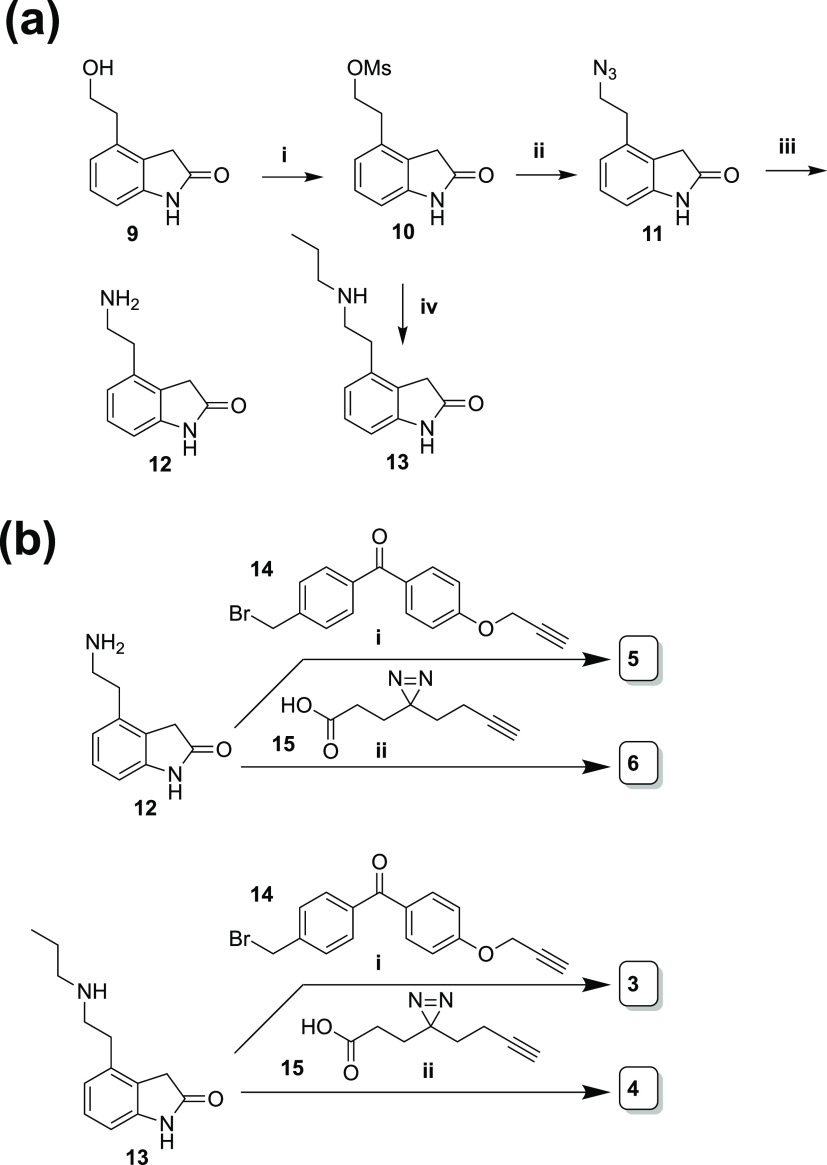
Synthesis of Ropinirole-Based Targets with Multifunctional Cross-Linkers (a) (i) MsCl, TEA, CH_2_Cl_2_, 74%; (ii) NaN_3_, H_2_O, 72%; (iii) polymer-bound PPh_3_, 22%; (iv) *N*-propylamine, reflux 29%. (b) (i) CsCO_3_, KI, **14**, 33% for **5**, 10% for **3**; (ii) EDC-HCl, HATU, DIPEA, **15**, 27% for **6**; 32% for **4**

The synthesis of ropinirole derivatives started with the commercially available 4-substituted hydroxyindole **9** (Scheme 1a). With the goal of substituting the alcohol for an amino group as in published ropinirole syntheses, we began by mesylating the alcohol to provide **10**. This is because, in our hands, tosylation of **9** gave a mixture of products, which were challenging to separate via chromatography, whereas mesylate **10** synthesis proceeded cleanly. The yield suffers some from the likely competing elimination reaction; this may be due to the easier formation of an antiperiplanar conformation for a more facile E2 elimination reaction versus syn elimination.^39^ The mesyl was displaced with sodium azide to provide **11**, which was then reduced to amine **12** using polymer-bound triphenylphosphine. To alkylate or acylate amines **12** and **13** (Scheme 1b), we synthesized known linkers **14** and **15**.

**Scheme 2:**
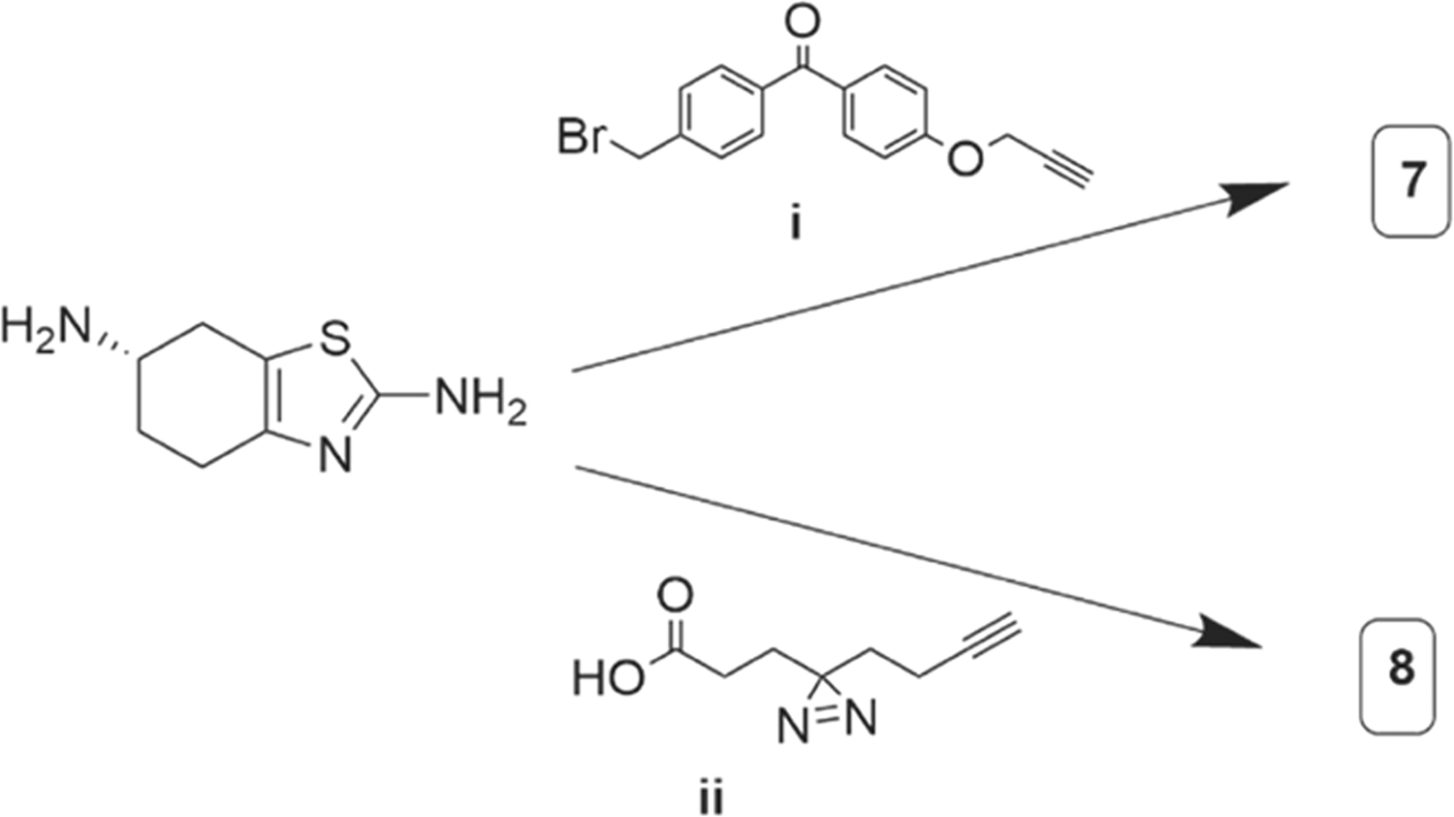
Synthesis of Pramipexole-Based Targets with Multifunctional Cross-Linkers. (i) CsCO_3_, KI, **14**, 79%; (ii) EDC-HCl, HATU, DIPEA, **15**, 45%

For pramipexole, derivatives were synthesized by alkylation or acylation of commercially available aminothiazole **2** to furnish a set of pramipexole derivatives (Scheme 2). With the probes in hand, we then turned our attention to biochemical analysis.

### 2.3 Pharmacological Analysis of Probes

DRD2 is able to transduce extracellular ligand binding into intracellular signals via a variety of effector molecules, notably the G_i_ protein and β-arrestin.^40,41^ We reasoned that if our probes could recapitulate the biological activity of the core pharmacophore, it was likely that they were binding the receptor in a similar fashion to the original receptor. We thus focused on these two pharmacologically important signal transduction pathways, G_i_ protein and β-arrestin, to determine how well our derivatized pramipexole and ropinirole recreated the activity of the original drug.

### 2.4 Intracellular Ca^2+^ Mobilization Assay

Intracellular calcium measurement was used to determine G-protein-mediated signaling by DRD2. Because DRD2 is linked to the G-protein subtype G_i_, agonist binding inhibits adenyl cyclase activity.^42^ To mitigate this effect, we created an HEK293T cell line stably expressing human DRD2, long form, and a chimeric G-protein, G_qi_, which alters the DRD2 receptor’s G-protein coupling so that signaling can occur through G_q_, resulting in an intracellular calcium flux.^43^ The assay is performed by first loading the cell line with a calcium-sensing dye. Binding of the ligand to the receptor results in an influx of calcium to the cytoplasm, which can be characterized in real time by monitoring the increase in dye fluorescence with confocal microscopy.

Our calcium flux bioactivity data, shown in Figure 2, suggest regions on the pramipexole and ropinirole pharmacophores that are highly tolerant toward extensive elaboration into a multifunctional probe, as well as those necessary for receptor binding. We found that ropinirole derivative **5** and pramipexole derivative **7** are active with low micromolar potency. Both probes have extensive bulk and molecular weight added to the basic nitrogen. The key requirement appears to be the retention of basicity of the nitrogen within the context of the pharmacophore, as evidenced by the greatly diminished activity of probes **4** and **8**. These probes contain chemically similar substitutions, except for the employment of an amide bond to derivatize the key nitrogen. The activity constants are tabulated in Table 1.

**Table 1:** Constants from Ca Assay^a^

compound	EC_50_	*R*^2^
5	1.67 μM	0.95
7	2.19 μM	0.95
ropinirole	124 nm	0.95
pramipexole	3.72 nm	0.96

a *R*^2^ value is calculated using nonlinear regression.

### 2.5 PRESTO-TANGO Assay

**Figure 3:**
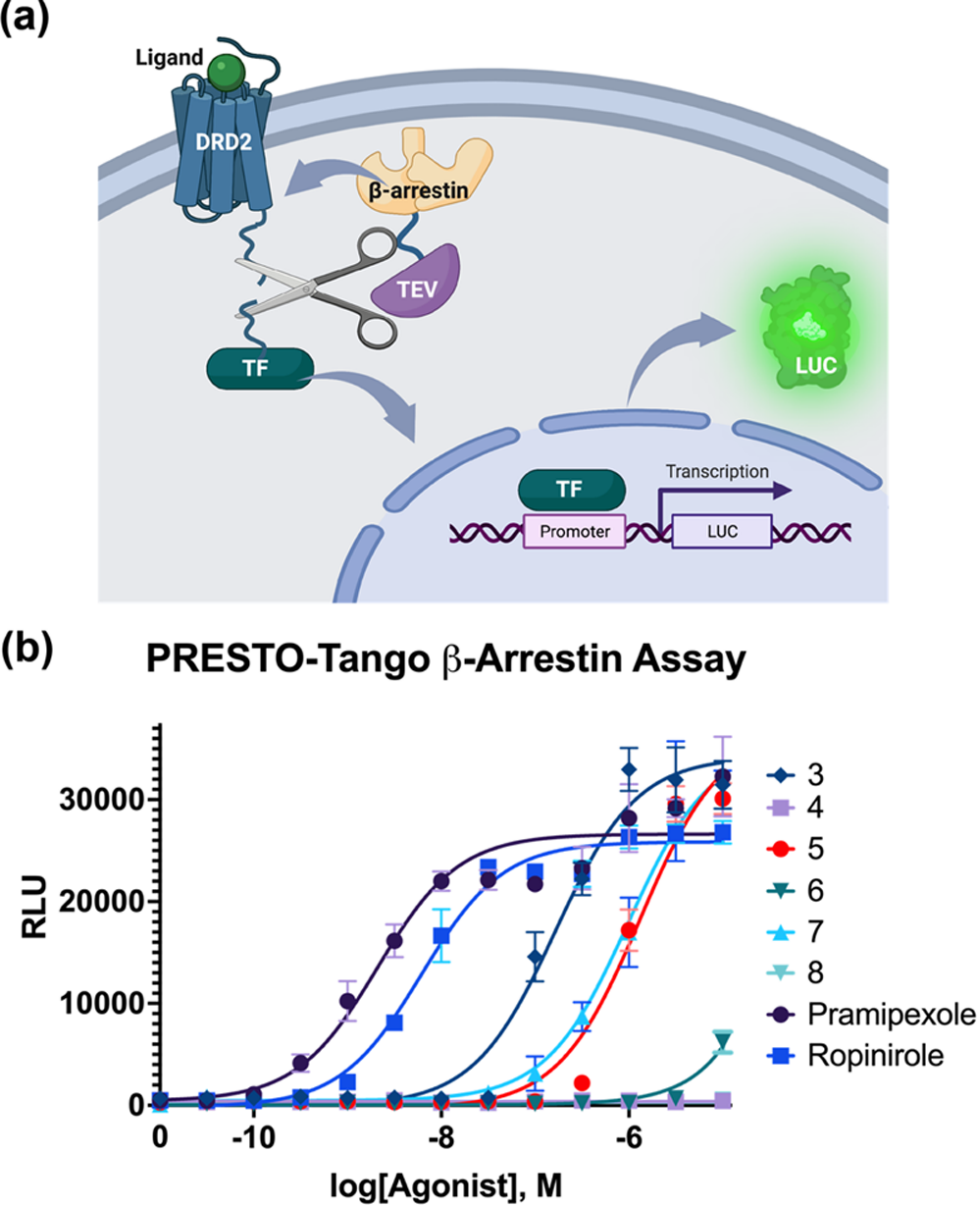
β-Arrestin recruitment analysis. (a) PRESTO-TANGO assay schematic: a ligand binds a chimeric DRD2 receptor, which then recruits β-arrestin fused with a TEV protease. The protease cuts a site between the receptor and a fused transcription factor, which then transits to the nuclease to initiate transcription of a luciferase gene. The luciferase activity is subsequently quantified. (b) Agonism of β-arrestin recruitment is quantified in EC_50_ curves via the detection of luciferase activity, using a Hill slope of 1.0.

To characterize the response of the β-arrestin effector pathway to our ligands, we used the Parallel-Receptor-ome Expression and Screening via Transcriptional Output TANGO (PRESTO-TANGO) assay developed in the Roth lab.^44,45^ This assay uses luciferase activity to monitor β-arrestin recruitment by DRD2 (Figure 3a).

The β-arrestin recruitment data (Figure 3b) indicate that probes **5** and **7** are again the most active derivatives, like what was found with the calcium assay. However, in contrast to the calcium assay, both pramipexole and ropinirole demonstrate low nanomolar activity, and probes **5** and **7** exhibit low micromolar activity, a difference of approximately ∼1000× for both probes vs their parent compounds. This is different than the calcium assay, where ropinirole and **5** instead showed only an approximate 10× difference. This may indicate that the substitutions on **5** are more disruptive of receptor binding interactions involving G-protein signaling vs β-arrestin. The activity constants are tabulated in Table 2.

**Table 2:** Constants from PRESTO-TANGO Assay^a^

compound	EC_50_	*R*^2^
5	1.32 μM	0.96
7	990 nm	0.97
ropinirole	6.14 nm	0.99
pramipexole	2.09 nm	0.94

a *R*^2^ value is calculated using nonlinear regression.

### 2.6 Colocalization Analysis via Confocal Microscopy of Cells

**Figure 4:**
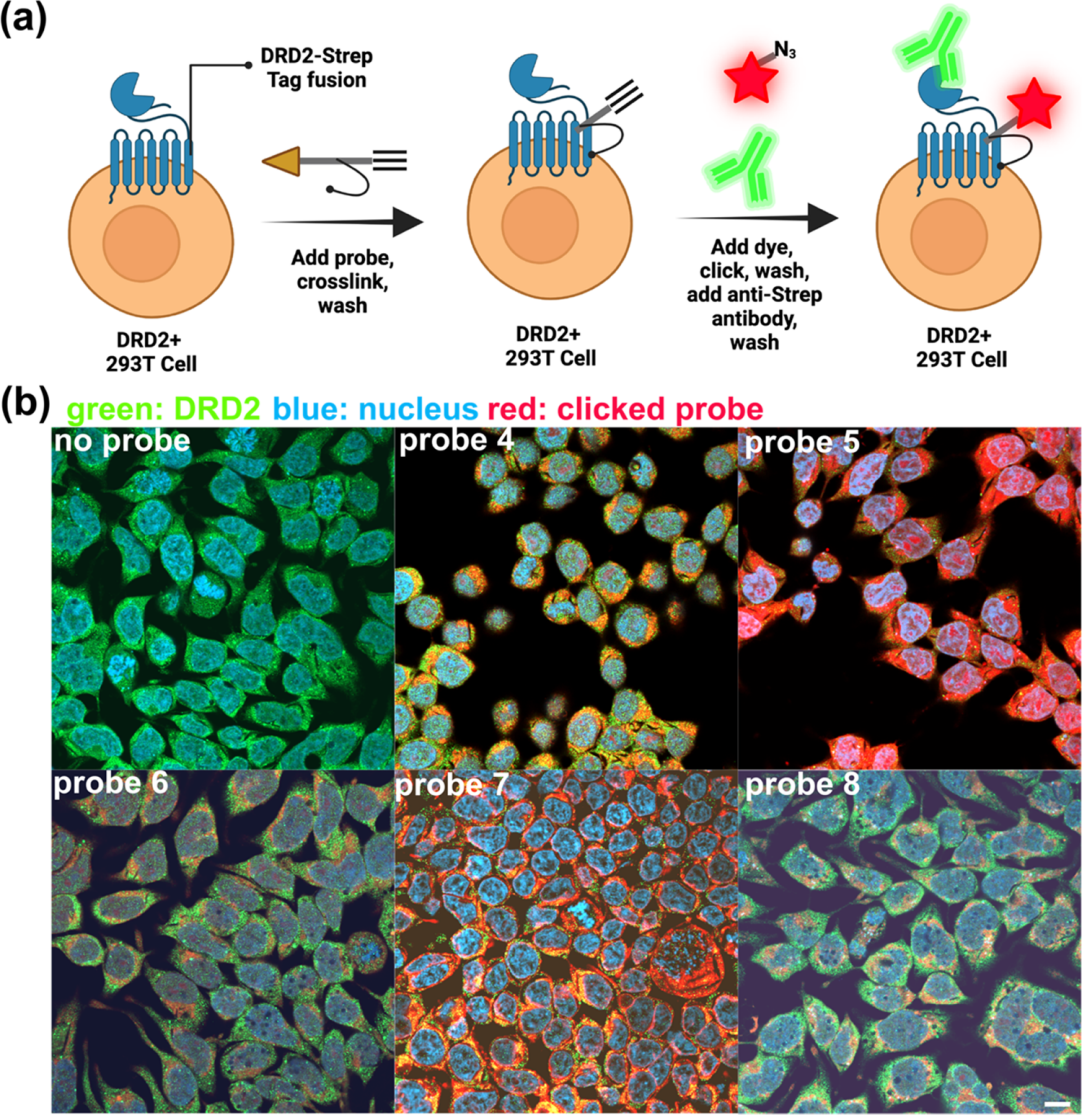
Photo-cross-linking of dye-clicked probe: confocal microscopy. (a) Schematic of the methodology used in the labeling process. Cells stably expressing DRD2 fused to an N-terminal Strep Tag II are treated with DRD2-targeting probes **5** or **7** at 5 μM, photo-cross-linked, and excess probe is washed out. An Alexa Fluor 555 azide is then clicked to the probe, washed out, and cells are treated with an anti-Strep-Tag II antibody and fluorescent secondary to visualize DRD2. Nuclei were stained with DAPI. (b) Confocal microscopy results of labeled cells. All probes show some degree of labeling. However, probes **5** and **7** show a notable increase in the labeling density. Images taken with 40× magnification, scale bar: 10 μm.

After the biochemical characterization of the dynamic interaction between the probe and target, we set out to characterize the static interaction: colocalization of probe and receptor in cells, with an emphasis on both on- and off-target labeling. Specifically, due to the known propensity for photo-cross-linking groups to nonspecifically label, we wanted a holistic view of how the probes interacted with whole cells. Thus, we treated our DRD2-expressing cell line with a 5 μM solution of the probe, photo-cross-linked, “clicked” on an azido fluorophore, and labeled the DRD2 receptor via an antibody conjugated to a complementary fluorophore (Figure 4a). Our results (Figure 4b) indicate that there is indeed background labeling with the probes. However, it was also possible to see some qualitative differences—probes **5** and **7** appeared to label DRD2-expressing cells with a higher avidity relative to the other probes. We therefore proceeded to quantify the specific vs nonspecific binding of the probe.

### 2.7 Flow Cytometry Quantification of Probe Labeling of Cells

**Figure 5:**
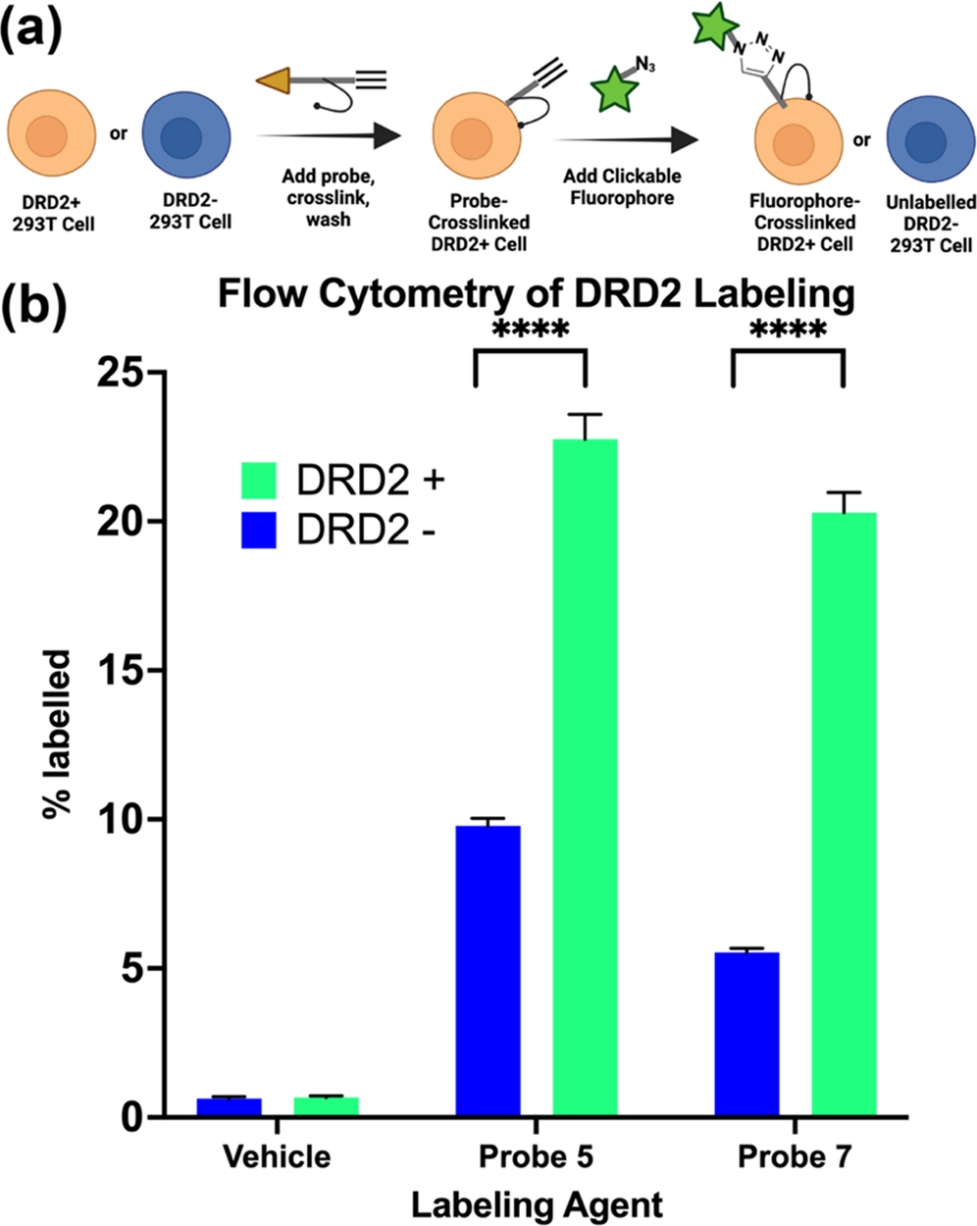
Flow cytometry quantification of probe labeling. (a) Schematic of flow cytometry workflow. (b) DRD2-expressing 293T cells or untransduced 293T cells (negative control) are treated with a 100 nm probe, which is photo-cross-linked, and an Alexa Fluor 555 azide is then “clicked” onto the probe. The cells were then analyzed by flow cytometry. *P* values determined using two-way ANOVA in GraphPad; **** corresponds to *P* < 0.0001.

Flow cytometry was used to quantify the number of cells that were successfully labeled by probes **5** and **7** as these were the most promising biochemically and seemed to display the highest propensity for labeling DRD2-expressing cells. To perform the quantification, our 293T cell line-expressing DRD2 or unmodified 293T cells as a negative control were treated with probes at a 100 nm concentration, the lower concentration than the EC_50_ determination, likely due in large part to the covalent nature of the binding event versus the noncovalent EC_50_ assays. The probes were covalently cross-linked to the cells with UV, and the unbound probe was washed out. The azide-containing fluorescent dye was then clicked onto the probe, the unbound dye was washed out, and the cells were analyzed by flow cytometry (Figure 5a). The results show that there is a highly statistically significant increase in the labeling of cells expressing DRD2 over those that do not (Figure 5b), with a *P-*value of less than 0.0001. High background labeling is well known for photo-cross-linking methodologies and has been extensively discussed in the literature.^23−25^ In these experiments, we were encouraged to see that the inclusion of the DRD2-binding pharmacophore enhanced the probes’ selectivity for DRD2-expressing cells.

### 2.8 Western Blot Analysis of DRD2-Probe Binding

**Figure 6:**
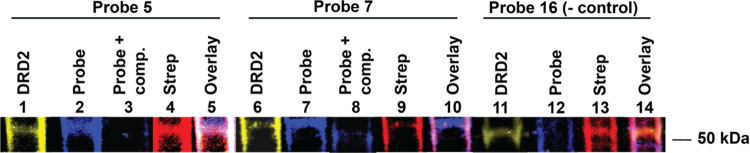
Photo-cross-linking of probes **5** and **7** to DRD2 visualized with Western blot. Lanes 1–5 correspond to samples with probe **5** at 100 nm, lanes 6–10 correspond to samples treated with probe **7** at 100 nm, and lanes 11–14 correspond to samples treated with negative control probe **16**. Lanes: 1, 6, and 11 are the anti-DRD2 antibody channel, lanes 2, 3, 7, 8, and 12 are fluorescence of the Alexa Fluor 555 clicked to the probes **5**, **7**, or **16**, lanes 4, 9, and 13 are the anti-Strep Tag antibody channel, and lanes 5, 10, and 14 are the overlaid channels for the respective probes.

To observe the photo-cross-linking of DRD2 at the protein level, we analyzed the colocalization of DRD2 by clicking a fluorophore on the probe and performing Western blots. We further observed the competition with the parent pharmacophore of each probe and the ability of a negative control benzophenone (**16**) to cross-link to DRD2. Our DRD2-expressing cells or unmodified 293T cells (control group) were suspended in media, treated with the probe at a 100 nm concentration, and photo-cross-linked. The cells were then lysed, and the membrane fraction was separated and enriched for DRD2 by pulldown of the Strep Tag II fusion using magnetic beads. After “clicking” a fluorescent tag to the probe, the membrane fraction was run on an SDS-PAGE gel, transferred to a nitrocellulose membrane, and treated with antibodies against DRD2 and Strep Tag II. The Western blot (Figure 6 and Supporting Information Figure S1) showed that the probe fluorescence colocalized with the signal for the anti-DRD2 antibody, suggesting that the probe was binding DRD2. We also observed many nonspecific binding bands for the probe, which is consistent with our other studies, as well as the observations of the field when it relates to photo-cross-linking groups. The competition assay showed that a 50 μM concentration of competitor was sufficient to greatly diminish the binding of **5** and **7**, indicating that the probes bind at the same site as the parent pharmacophore. The negative control probe **16** did not show colocalization with the DRD2 band. Overall, these results supported the conclusion that probes **5** and **7** were binding to the DRD2 receptor. With this confirmation, we proceeded to proteomic analysis of probe interactions.

### 2.9 Identification of Primary Rat Brain Protein Networks Covalently Modified by Probes (Interactome) via Mass Spectrometry Proteomics

**Figure 7:**
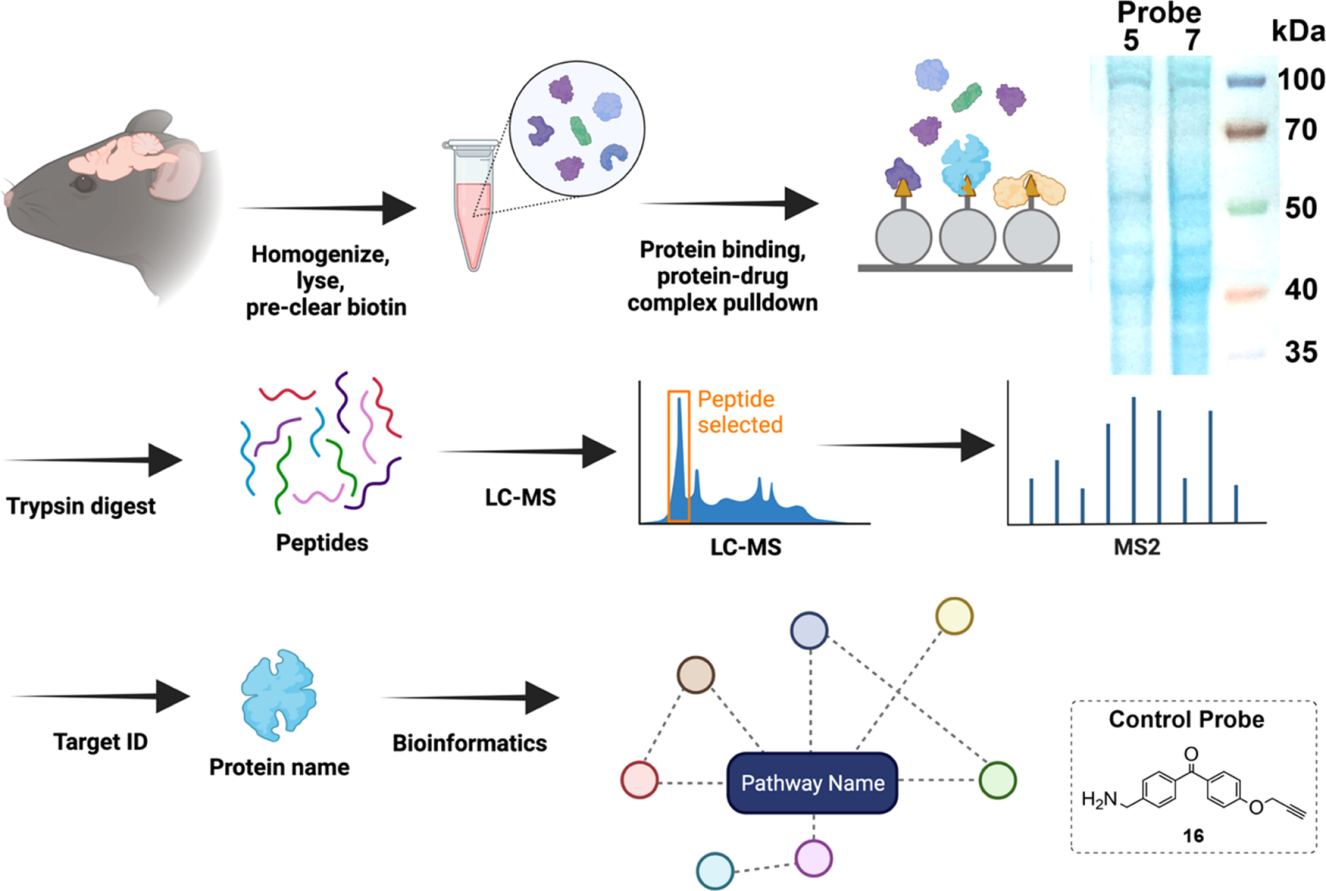
Workflow for affinity purification and proteomic analysis. Whole brain tissue is homogenized and lysed, and endogenous biotin is removed. The lysate is treated with probe, photo-cross-linked, and biotin is clicked onto the probe. Streptavidin beads pull down proteins and their interactors via the linked biotin. An example Coomassie-stained gel of resulting proteins is shown in the inset. The proteins are trypsin-digested while on-bead, eluted, desalted, and peptides are run on LC-MS. Proteomic analysis (SEQUEST) is performed to ID the proteins, followed by bioinformatic analysis of functional protein association networks (KEGG, STRING).

We used a combination of affinity purification and proteomic analysis to identify the pathways that associate with proteins bound by probes **5** and **7**, following the workflow shown in Figure 7. DRD2 is a membrane-bound GPCR, and, as mentioned previously, it is known to be exceedingly difficult to study in isolation from its host cell. Further, DRD2 expression levels differ widely according to brain region, layer, and other biochemical variables.^46−48^ Thus, to validate our approach, we first determined whether we could detect the DRD2 expression using our mutant cell line, which exogenously expresses DRD2. We found that DRD2 protein could indeed be detected via mass spectrometry following photo-cross-linking of probe and biotin–streptavidin pulldown. We were encouraged that we were able to detect the DRD2 protein, although we were unable to determine the specific peptide modified by our probes (see Supporting Information Excel File 1). With these data, we proceeded to our experiments in primary cells.

Whole brain assays were designed to assay the binding partners of **5** and **7**, as well as a control for the inherent binding of the benzophenone cross-linker using molecule **16** (Figure 7). Here, we use a protein extraction methodology that does not bias the assay toward the membrane fraction. We chose this methodology to maximize the amount of unique hits we were likely to record. A drawback to this assay, however, is that GPCRs need highly specific extraction/purification conditions to be detected by LC-MS (or other protein detection methodologies) at the end point. Thus, by biasing our assay toward a “wide lens” to record a picture of diverse interactions, we missed the specific focus necessary to observe DRD2 and structurally related GPCRs, and we were unable to directly observe DRD2 in the proteomic analysis.

To perform the assay, the whole brain minus the olfactory bulb from an adult female Sprague Dawley rat was homogenized and treated with probes or linker control **16** at a 50 μM concentration and photo-cross-linked. A streptavidin resin was used to deplete endogenous biotin and then clicked with azido biotin, followed by streptavidin pulldown. Coomassie gels of the pulldowns are shown in Figure 7. The streptavidin beads were processed by tryptic digest, followed by LC/MS proteomic analysis. Proteins identified in both control **16** and the **5** and **7** treated samples were removed as background (for full results, see the Supporting Information, Excel File 2). We found that probes **5** and **7** had 58 proteins in common, probe **5** showed 81 unique proteins, and probe **7** had 57 hits. All targets (peptides and proteins) were identified with a maximum false discovery rate of 5% or a *q*-value of >0.05. Most peptides and proteins were identified with a false discovery rate of 1%, *q*-value of >0.01 (Supporting Information, Excel File 2).

γ-Aminobutyric acid receptor subunit α1 and neuronal nitric oxide synthase (nNOS) were two of the most interesting hits in common between the two probes. γ-Aminobutyric acid is the major inhibitory neurotransmitter, and the γ-aminobutyric acid receptor subunit α1 is the target of sedative/hypnotic drugs.^49−51^ nNOS catalyzes the production of the neurotransmitter nitric oxide in the brain. Nitric oxide regulation contributes to a variety of physiological states, such as long-term potentiation, and diseases, such as schizophrenia.^52,53^ The inclusion of these neurotransmission-associated proteins as hits in our pulldown assay for both the ropinirole- and pramipexole-derived probes suggests that these neurotransmitter pathways may be contributing to the pharmacology of these drugs. In the hits unique to each of the probes, for **5** two of the standout hits were the muscarinic acetylcholine receptor M1 and CB1 cannabinoid receptor-interacting protein 1. The muscarinic acetylcholine receptors are GPCRs for the neurotransmitter acetylcholine. These receptors are critical to the fundamental neurological function, in addition to being effective drug targets.^54^ Endogenous cannabinoids typically act presynaptically to suppress neurotransmitter release, and endocannabinoid receptors are GPCRs and are abundantly expressed in the brain.^55^ CB1 cannabinoid receptor-interacting protein 1 competes with β-arrestin for binding to the cannabinoid receptor and may inhibit β-arrestin-mediated internalization of the cannabinoid receptor.^56^ These proteins may play a role in eliciting distinctive physiological responses for ropinirole.

As many of the most medically relevant brain proteins (including DRD2) have highly variable and exquisitely controlled expression levels, their presence at any one point in time may be extremely low in abundance. Therefore, we used bioinformatic analysis on the LC/MS hits to understand the broader pathways that may be targeted by these probes. In an initial search, we determined pathways that ropinirole and pramipexole are already known to interact with, via the STICH platform, which catalogs drug–pathway interactions, and KEGG database, which here we used to determine disease-associated pathways.^57−64^ For ropinirole, KEGG analysis indicated that in addition to the anticipated dopaminergic synapse and neuroactive ligand–receptor interaction pathways, ropinirole also interacts with the tryptophan metabolism, cocaine addiction, alcoholism, gap junction, and chemical carcinogenesis pathways. For pramipexole, the dopaminergic synapse, neuroactive ligand–receptor interaction, cocaine addiction, alcoholism, and gap junction pathways are again represented, with the addition of the serotonergic synapse pathways via the serotonin receptors Htr2a and Htr2c (see Supporting Information Figures S2 and S3 and Tables S1 and S2 for further details). With this information in mind, we then turned to analyzing the pathways represented by the hits from our LC/MS experiments.

For the hits in common between probes **5** and **7**, KEGG analysis indicated that there was significant enrichment in proteins from pathways directly involved in Parkinson’s disease, Alzheimer’s disease, and amyotrophic lateral sclerosis, among others (see Supporting Information Table S3). These data serve as a positive confirmation that probes **5** and **7** are recapitulating the mechanism of action of their parent pharmacophores, which are Parkinson’s disease treatments. We next analyzed and visualized the biochemical connections between hits using the STRING database. The hits had significantly more interactions than expected for a random collection of proteins (*p*-value < 1 × 10^–16^; Supporting Information Table S4). There was significant enrichment in Parkinson’s disease, Alzheimer’s disease, regulation of ornithine decarboxylase and RAS signaling pathways, parkin–ubiquitin pathway (early-onset Parkinson’s disease), and oxidative phosphorylation pathways, among others (see Supporting Information Tables S4 and S5).^65^

**Figure 8:**
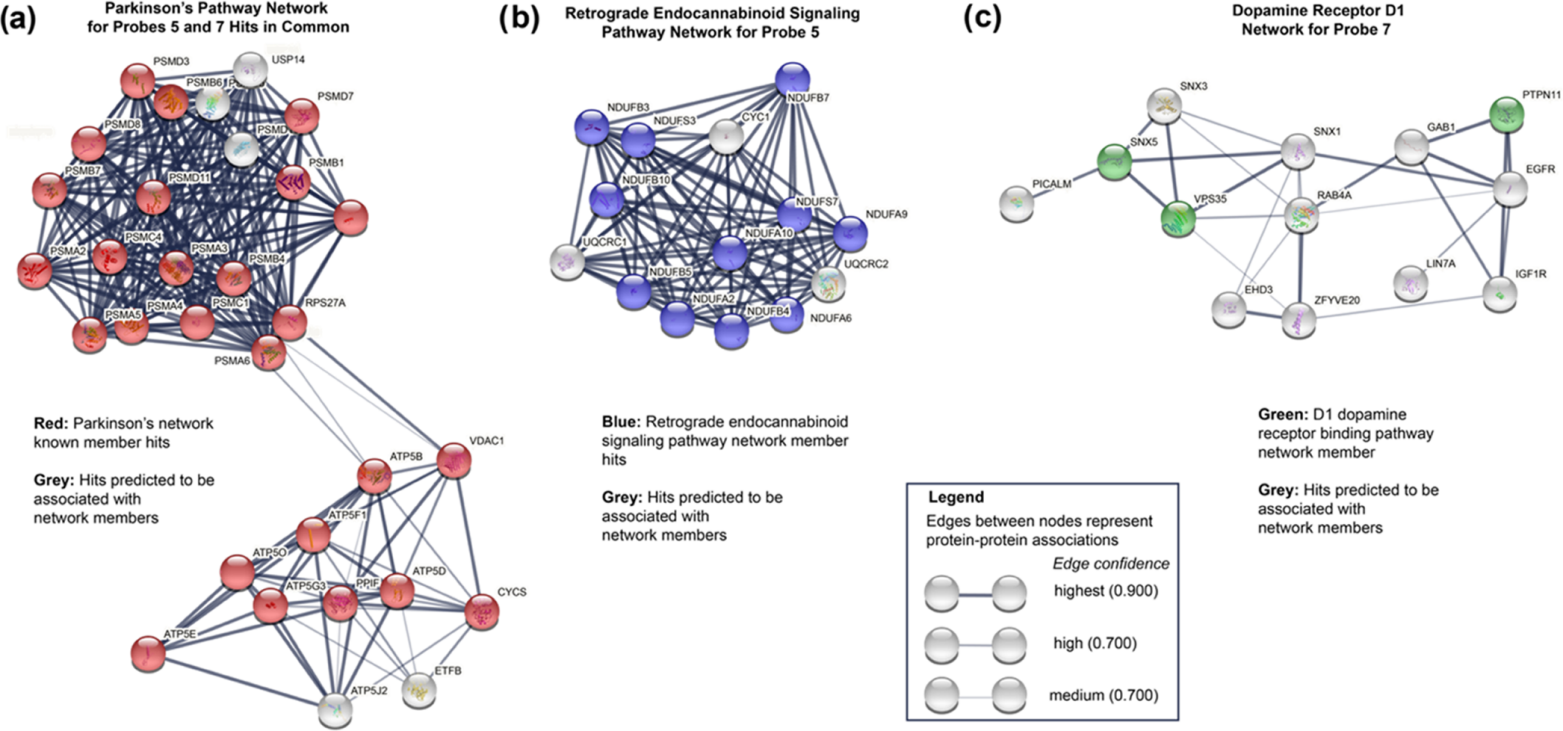
Protein networks identified by bioinformatic analysis. (a) Both probes **5** and **7** hit multiple genes in Parkinson’s disease network according to STRING and KEGG analyses. (b) Probe **5** (ropinirole-based) hit many genes in the retrograde endocannabinoid signaling pathway according to STRING and KEGG analyses. (c) Probe **7** (pramipexole-based) hit genes in the dopamine receptor D_1_ (DRD1) signaling network according to STRING and gene ontology analyses.

For hits unique to probe **5**, KEGG analysis indicated retrograde endocannabinoid signaling, Huntington’s disease, and nonalcoholic fatty liver disease among others (Supporting Information Table S8). The endocannabinoid pathway members targeted by **5** were visualized by STRING (Figure 8, Supporting Information Table S7). The targeting of this pathway is particularly intriguing as this mechanism suppresses neurotransmitter release, as well as regulating motor control.^66^ We then analyzed hits unique to **7**. A combination of molecular function (gene ontology) analysis and STRING analysis indicated that the dopamine receptor D1 receptor binding pathway as a hit (Supporting information, Tables S9 and S10). Dopamine receptor D1 is bound poorly by pramipexole. However, dopamine receptors dimerize, multimerize, and oligomerize, and our assay may be detecting some of the more biochemically robust members of this pathway: the cytosolic intracellular signal transduction proteins.^67,68^ These combined data suggest that there may be multiple disease-related and neurological targets for these drugs, which may contribute to their overall pharmacology.

## 3 Conclusions

In conclusion, the design and use of bioactive photoaffinity probes for DRD2 were demonstrated. These probes demonstrated excellent activity in “workhorse” biochemical assays used for traditional dopamine receptor-targeted drugs, as well as chemical–biological techniques of receptor and cell labeling, and chemiproteomics. Most drugs have a variety of targets that may be missed by focusing on a small set of biochemical assays or looking at drug activity through the lens of a particular disease symptom. Here, our work reinforces the notion that omics-based approaches, which provide a broad picture of a molecule’s “interactome”, may also give insight into the pleiotropy of effects observed for a drug or perhaps indicate new applications.^22,69−71^ Specifically, probes **5** and **7** bound other protein networks including the retrograde endocannabinoid signaling pathway, neuronal nitric oxide synthase, GABA receptor components, and muscarinic acetylcholine receptor M1. Follow-up analysis may yield insights into how this pathway relates specifically to Parkinson’s disease symptoms or provide new targets for treatments.

## 4 Methods

### 4.1 Chemistry

For general synthesis methods, see the Supporting Information. Compounds **14** and **15** were synthesized as described previously; compound **16** was obtained commercially from Sigma-Aldrich.^72,73^

#### 4.1.1 2-(2-Oxoindolin-4-yl)ethyl Methanesulfonate (**10**)

To a solution of 4-(2-hydroxyethyl)oxyindole (10 g, 56.4 mmol) in pyridine (22 mL, 282 mmol) was added methanesulfonyl chloride (5.24 mL, 67.7 mmol) in CH_2_Cl_2_ (50 mL) dropwise at 5–10 °C. The reaction was stirred at this temperature for 3 h, then aqueous NaHSO_4_ (50 mL) was added, and the organics were extracted with CH_2_Cl_2_ (3 × 50 mL). The combined organic layers were washed with saturated aqueous Na_2_CO_3_, water, saturated aqueous NH_4_Cl, and brine. The layers were then dried over MgSO_4_, filtered, and concentrated in vacuo to yield 10 as a pale yellow solid (12.7 g, 49.9 mmol, 88%). ^1^H NMR (CDCl_3_) δ 8.63 (s, 1H), 7.22 (t, *J* = 7.8 Hz, 1H), 6.91 (d, *J* = 7.6 Hz, 1H), 6.83 (d, *J* = 7.6 Hz, 1H), 4.46 (t, *J* = 6.8 Hz, 2H), 3.54 (s, 2H), 3.03 (t, *J* = 6.7 Hz, 2H), 2.93 (s, 3H); ^13^C NMR (100 MHz, acetone-*d*_6_) δ 177.3, 142.7, 132.8, 128.5, 124.7, 122.8, 108.7, 68.9, 37.5, 35.1, 32.9; HRMS (ESI+) *m*/*z* calcd for fragment C_11_H_13_NO_4_S [M + H]^+^ 256.06, found 256.0644.

#### 4.1.2 4-(2-Azidoethyl)indolin-2-one (**11**)

To a solution of mesylate **10** (1.53 g, 5.99 mmol) in DMF (234 mL) was added NaN_3_ (1.17 g, 18.0 mmol) and the reaction mixture was refluxed at 60 °C. After 5 h, water (1000 mL) and diethyl ether (500 mL) were added, and the phases were separated. The aqueous layer was extracted with diethyl ether (2 × 100 mL), and the combined organics were washed with water, dried over MgSO_4_, and concentrated in vacuo. Purification by column chromatography (silica, 40% EtOAc in hexanes) afforded **11** as a yellow solid (573 mg, 2.83 mmol, 72%). ^1^H NMR (400 MHz, CDCl_3_) δ 9.02 (bs, 1H), 7.19 (t, *J* = 8.0 Hz, 1H), 6.88 (d, *J* = 7.6 Hz, 1H), 6.81 (d, *J* = 7.6 Hz, 1H), 3.55 (t, *J* = 6.8 Hz, 2H), 3.52 (s, 2H), 2.82 (t, *J* = 6.8 Hz, 2H); ^13^C NMR (100 MHz, CDCl_3_) δ 177.2, 142.6, 134.6, 128.4, 124.4, 122.6, 108.3, 51.4, 35.0, 32.5; HRMS (ESI+) *m*/*z* calcd for fragment C_10_H_11_ON_4_ [M + H]^+^ 203.09, found 203.0934.

#### 4.1.3 4-(2-Aminoethyl)indolin-2-one **(12)**

To a solution of **11** (1.25 g, 6.18 mmol) in THF (100 mL) were added water (0.67 mL, 37.11 mmol) and resin-linked triphenylphosphine (mesh, 3 mmol/g, 4.12 g), and the resulting slurry was stirred gently at 85° C overnight. The reaction mixture was then filtered by gravity, rinsed 3× with THF, and concentrated in vacuo. The resulting residue was then dissolved in 1 M NaHSO_4_ (80 mL) and poured into a separatory funnel containing 80 mL of diethyl ether. The aqueous layer was basified to pH 9 with 2 M NaOH, extracted with ethyl acetate (3 × 80 mL), dried over MgSO_4_, filtered, and concentrated in vacuo. The resulting oil was purified by column chromatography (Biotage Sfar KP-Amino, 0–10% MeOH in CH_2_Cl_2_) yielding **12** as a light brown solid (240 mg, 1.36 mmol, 22%). ^1^H NMR (400 MHz, acetone-*d*_6_) δ 9.30 (bs, 1H), 7.10 (t, 1H, *J* = 8 Hz), 6.84 (d, 1H, *J* = 8.4 Hz), 6.72 (d, 1H, *J* = 8 Hz), 3.48 (s, 2H), 3.44 (t, 2H, *J* = 7.6 Hz), 2.81 (t, 2H, *J* = 6.8 Hz), 2.82 (bs, 2H); ^13^C NMR (100 MHz, D_2_O) δ 180.1, 142.5, 133.2, 128.4,124.9, 122.9, 109.0, 39.2, 30.8, 23.2; HRMS (ESI+) *m*/*z* calcd for fragment C_10_H_12_N_2_O [M + H]^+^ 177.09, found 177.1019.

#### 4.1.4 2-(2-Oxoindolin-4-yl)ethyl Methanesulfonate (**13**)

Compound **13** was synthesized from **10** according to the general method of Capuano et al.^33^ The spectra matched those reported.

#### 4.1.5 4-(2-((4-(4-(Prop-2-yn-1-yloxy)benzoyl)benzyl)(propyl)amino)ethyl)indolin-2-one (**3**)

Compound **3** was made according to the general method of Chen et al., with some modifications.^74^ A 10 mL round-bottomed flask was charged with **13** (100 mg, 0.46 mmol), benzophenone cross-linker **14** (158 mg, 0.46 mmol), cesium carbonate (150 mg, 0.46 mmol), and potassium iodide (115 mg, 0.69 mmol). Acetonitrile (3 mL) was added to the mixture, and the reaction was stirred under reflux at 85 °C for 2 h. The solvent was evaporated in vacuo, washed with water (5 mL), and extracted with ethyl acetate (3 × 5 mL). The combined organic layers were washed with water and brine, dried over MgSO_4_, filtered, and concentrated. The resulting crude product was purified by column (silica, 2.5% MeOH in CH_2_C_l2_) to yield **3** as an amorphous orange solid (71 mg, 0.015 mmol, 33%) (400 MHz, acetone-*d*_6_) δ 9.29 (bs, 1H), 7.82 (d, *J* = 8.8 Hz, 2H), 7.67 (d, *J* = 8.2 Hz, 2H), 7.46 (d, *J* = 8.1 Hz, 2H), 7.17 (d, *J* = 8.8 Hz, 2H), 7.09 (t, *J* = 7.8 Hz, 1H), 6.79 (d, *J* = 7.8 Hz, 1H), 6.71 (d, *J* = 7.7 Hz, 1H), 4.93 (d, *J* = 2.4 Hz, 2H), 3.75 (s, 2H), 3.31 (s, 2H), 3.16 (t, *J* = 2.4 Hz, 1H), 2.74 (m, 4H), 2.57 (t, *J* = 7.2 Hz, 2H), 1.56 (m, 2H), 0.91 (t, *J* = 7.4 Hz, 3H).; ^13^C (100 MHz, CDCl_3_) δ 195.3, 177.1, 160.9, 144.7, 142.3, 137.1, 136.7, 132.5, 132.4, 131.2, 129.9, 128.3, 128.0, 123.9, 123.1, 114.4, 107.5, 77.9, 76.1, 58.5, 55.9, 53.9, 34.9, 31.0, 20.4, 11.9. HRMS (ESI+) *m*/*z* calcd for fragment C_30_H_30_N_2_O_3_ [M + H]^+^ 467.23, found 467.2315.

#### 4.1.6 3-(3-(But-3-yn-1-yl)-3*H*-diazirin-3-yl)-*N*-(2-(2-oxoindolin-4-yl)ethyl)-*N*-propylpropanamide **(4)**

Compound **4** was made according to the general method of Saghatelian et al., with modifications.^75^ To a 5 mL vial containing 3-(3-(but-3-yn-1-yl)-3*H*-diazirin-3-yl)propanoic acid **15** (25 mg, 0.15 mmol) in CH_2_Cl_2_ (2 mL), amine **13** (42 mg, 0.16 mmol), DIPEA (79 μL, 0.45 mmol), EDC-HCl (43 mg, 0.22 mmol), and HATU (86 mg, 0.23 mmol) were added. The reaction mixture was stirred at room temperature overnight in the dark for 24 h and concentrated in vacuo. The crude residue was diluted with CH_2_Cl_2_ (5 mL), washed with water (5 × 10 mL) and brine, then dried over anhydrous Na_2_SO_4_, and volatiles were removed in vacuo. The resulting oil was purified by flash column chromatography (silica, 70% EtOAc in hexanes) to provide **4** as a colorless solid (14.81 mg, 0.04 mmol, 27% yield). ^1^H NMR (600 MHz, CDCl_3_) ^1^H NMR (600 MHz, CDCl_3_) isomer 1 (66%): δ 8.42 (bs, 1H), 7.14 (t, *J* = 7.7 Hz, 1H), 6.85 (d, *J* = 7.8 Hz, 1H), 6.74 (d, *J* = 7.7 Hz, 1H), 3.51 (s, 2H), 3.47 (t, *J* = 7.8 Hz, 2H), 3.10 (t, *J* = 7.6 Hz, 2H), 2.77 (m, 2H), 2.03 (t, *J* = 7.3 Hz, 2H), 1.98 (m, 2H), 1.87 (t, *J* = 7.1 Hz, 2H), 1.81 (m, 1H), 1.66 (t, *J* = 7.4 Hz, 2H), 1.58 (t, *J* = 7.3 Hz, 2H), 0.90 (t, *J* = 7.4 Hz, 3H); isomer 2 (33%): δ 8.531 (bs, 1H), 7.19 (t, *J* = 7.7 Hz, 1H), 6.80 (d, *J* = 1.9 Hz, 1H), 6.79 (d, *J* = 1.9 Hz, 1H), 3.46 (s, 2H), 3.42 (t, *J* = 7.4 Hz, 2H), 3.29 (t, *J* = 7.7 Hz, 2H), 2.70 (m, 2H), 2.031 (t, *J* = 7.5 Hz, 2H), 2.03 (m, 2H), 1.73 (m, 3H), 1.58 (m, 2H), 1.54 (m, 2H), 0.88 (t, *J* = 7.6 Hz, 3H); 13C NMR (150 MHz, CDCl_3_); isomer 1 (66%): δ 177.4, 170.9, 142.6, 135.6, 128.2, 124.4, 122.9, 108.0, 82.9, 69.3, 50.2, 46.8, 38.8, 35.1, 32.7, 31.3, 28.0, 27.0, 22.3, 13.4, 11.4; isomer 2 (33%): δ 176.9, 170.7, 142.9, 134.6, 128.7, 124.1, 122.9, 108.5, 77.3, 69.3, 47.8, 47.7, 34.9, 32.7, 32.5, 28.1, 28.0, 26.7, 21.0, 13.4, 11.5. HRMS (ESI+) *m*/*z* calcd for fragment C_21_H_26_N_4_O_2_ [M + H]^+^ 367.21, found 367.2138.

#### 4.1.7 4-(2-((4-(4-(Prop-2-yn-1-yloxy)benzoyl)benzyl)amino)ethyl)indolin-2-one (**5**)

Compound **5** was made according to the general method of Chen et al., with modifications.^59^ The reaction vessel was charged with **12** (40 mg, 0.22 mmol), benzophenone **14** (78 mg, 0.22 mmol), cesium carbonate (108 mg, 0.33 mmol), and potassium iodide (55 mg, 0.33 mmol). Acetonitrile (5 mL) was added, and the reaction was stirred under reflux at 100 °C for 24 h, concentrated in vacuo, washed with water, and extracted with ethyl acetate. The combined organic layers were washed with brine and dried over MgSO_4_, filtered, and concentrated. The resulting crude residue was purified by flash column (silica, 0–10% MeOH in CH_2_Cl_2_) to afford product **5** as an orange oil (10 mg, 0.024 mmol, 10%). 1H NMR (400 MHz, acetonitrile-*d*_3_) δ 8.32 (bs, 1H), 7.78 (m, 2H), 7.67 (t, *J* = 7.2 Hz, 2H), 7.52 (m, 2H), 7.08 (m, 3H), 6.69 (t, *J* = 8.2 Hz, 1H), 4.84 (d, *J* = 2.4 Hz, 2H), 4.61 (m, 1H), 4.28 (m, 1H), 3.93 (s, 1H), 2.86 (t, *J* = 2.4 Hz, 1H), 2.27 (bs, 1H), 1.56 (s, 2H), 1.03 (s, 2H); ^13^C δ (100 MHz, acetonitrile-*d*_3_) δ 195.6, 177.9, 161.8, 147.6, 145.3, 142.8, 138.2, 137.4, 133.1, 133.0, 132.0, 131.6, 131.0, 130.5, 129.6, 128.9, 128.7, 123.0, 115.4, 115.2, 107.4, 79.1, 77.3, 56.7, 54.1, 45.6, 23.0 HRMS (ESI+) *m*/*z* calcd for fragment C_27_H_24_N_2_O_3_ [M + H]^+^ 425.18, found 425.1852.

#### 4.1.8 3-(3-(But-3-yn-1-yl)-3*H*-diazirin-3-yl)-*N*-(2-(2-oxoindolin-4-yl)ethyl)propanamide **(6)**

Compound **6** was made according to the general method of Saghatelian et al., with modifications.^75^ To a 5 mL vial containing 3-(3-(but-3-yn-1-yl)-3*H*-diazirin-3-yl)propanoic acid **15** (25 mg, 0.15 mmol) in CH_2_Cl_2_ (2 mL), amine **12** (29 mg, 0.16 mmol), DIPEA (79 μL, 0.45 mmol), EDC-HCl (43 mg, 0.22 mmol), and HATU (86 mg, 0.23 mmol) were added. The reaction mixture was stirred at room temperature overnight in the dark for 20 h and concentrated in vacuo. The crude residue was diluted with CH_2_Cl_2_ (5 mL), washed with water (5 × 10 mL) and brine, then dried over anhydrous Na_2_SO_4_, and volatiles were removed in vacuo. The crude residue was dissolved in acetonitrile and washed with hexanes, and the acetonitrile layer was evaporated to provide **6** as a colorless solid (17 mg, 0.052 mmol, 32%). ^1^H NMR (400 MHz, CDCl_3_) δ 7.83 (bs, 1 h), 7.18 (t, *J* = 8.0 Hz, 1H), 6.83 (d, *J* = 8.0 Hz, 1H), 6.69 (d, *J* = 7.6 Hz, 1H), 3.93 (s, 2H), 3.63 (d, *J* = 16.4 Hz, 1H), 3.10 (d, *J* = 5.2 Hz, 2H), 2.17 (m, 1H), 2.02 (td, *J* = 7.2 Hz, 1.6 Hz, 3H), 1.95 (t, *J* = 2.6 Hz, 1H), 1.86 (m, 4H), 1.65 (td, *J* = 7.6 Hz, 2.8 Hz, 2H); ^13^C (100 MHz, acetone-*d*_6_) δ 176.7, 172.0, 143.7, 136.3, 129.2, 126.0, 123.1, 107.2, 83.7, 70.4, 54.2, 42.6, 41.9, 38.7, 35.7, 33.3, 32.9, 13.6; HRMS (ESI−) *m*/*z* calcd for fragment C_18_H_20_N_4_O_2_ [M]^-^ 324.16, found 324.1545.

#### 4.1.9 (*S*)-(4-(((2-Amino-4,5,6,7-tetrahydrobenzo[*d*]thiazol-6-yl)amino)methyl)phenyl)(4-(prop-2-yn-1-yloxy)phenyl)methanone (**7**)

A 10 mL round-bottom flask was charged with **2** (42 mg, 0.25 mmol), benzophenone cross-linker **14** (83 mg, 0.25 mmol), cesium carbonate (123 mg, 0.38 mmol), and potassium iodide (56 mg, 0.38 mmol). Acetonitrile (5 mL) was added to the mixture, and the reaction was stirred at room temperature for 48 h. The solvent was evaporated in vacuo, washed with water, and extracted with ethyl acetate (3 × 5 mL). The combined organic layers were washed with water and brine, dried over MgSO_4_, filtered, and concentrated. The resulting crude product was purified by column (silica, MeOH/EtOAc, 10:90) to provide **7** as a yellow solid (66 mg, 0.052 mmol, 79%). ^1^H NMR (400 MHz, DMSO-*d*_6_) δ 7.74 (d, 2H, *J* = 6.2 Hz), 7.65 (d, 2H, *J* = 8.0 Hz), 7.52 (d, 2H, *J* = 8.0 Hz), 7.13 (d, 2H, *J* = 8.8 Hz), 6.59 (s, 2H), 4.92 (d, 2H, *J* = 2.4 Hz), 3.87 (s, 2H), 3.64 (d, 1H, *J* = 2.4 Hz), 2.76 (m, 2H), 2.46 (s, 1H), 2.32 (m, 2H), 1.96 (m, 1H), 1.56 (m, 1H), 1.22 (m, 1H); ^13^C NMR (100 MHz, DMSO-*d*_6_) δ 194.6, 166.3, 161.1, 146.6, 145.0, 136.3, 132.4, 130.8, 129.9, 128.3, 115.1, 113.5, 79.2, 79.1, 56.2, 53.5, 50.4, 31.1, 29.9, 29.5, 25.3; HRMS (ESI+) *m*/*z* calcd for fragment C_24_H_23_N_3_O_2_S [M + H]^+^ 418.15, found 418.1588.

#### 4.1.10 (*S*)-*N*-(2-Amino-4,5,6,7-tetrahydrobenzo[*d*]thiazol-6-yl)-3-(3-(but-3-yn-1-yl)-3*H*-diazirin-3-yl)propanamide (**8**)

To a 10 mL round-bottom flask containing 3-(3-(but-3-yn-l-yl)-3*H*-diazirin-3-yl)propanoic acid **15** (50 mg, 0.30 mmol) in CH_2_Cl_2_ (5 mL), **2** (56 mg, 0.33 mmol), DIPEA (157 μL, 0.9 mmol), EDC-HCl (87 mg, 0.45 mmol), and HOAt (172 mg, 0.45 mmol) were added. The reaction mixture was stirred at room temperature, protected from light, for 48 h. The reaction was diluted with CH_2_Cl_2_ (10 mL) and washed successively with saturated aqueous NH_4_CI (10 mL) and saturated aqueous NaHCO_3_ (10 mL), then dried over MgSO_4_, filtered, and concentrated. The resulting crude product was purified by column (silica, MeOH/EtOAc, 15:85) to yield **8** as a yellow solid (43 mg, 0.14 mmol, 45%). ^1^H NMR (400 MHz, DMSO-*d*_6_) δ 7.93 (d, 1H, *J* = 4.8 Hz), 6.64 (s, 2H), 3.96 (m, 1H), 2.73 (m, 2H), 2.36 (m, 2H), 1.95 (m, 5H), 1.62 (m, 5H), 1.24 (s, 1H); ^13^C NMR (100 MHz, DMSO-*d*_6_) δ 170.6, 166.5, 144.7, 112.8, 83.6, 72.2, 45.4, 31.9. 30.0, 29.2, 28.7, 28.6, 24.9, 13.1; HRMS (ESI+) *m*/*z* calcd for fragment C_15_H_19_N_5_OS [M + H]^+^ 318.13, found 318.1379.

### 4.2 Cloning of DRD2 Receptor Constructs

The transfer plasmid vector was derived from pHR-CMV-TetO2-IRES-mRuby2 (addgene #113885) linearized with the following primers: FORWARD 5′-TCCTGAAGATCCACTGCCTTGAGGTGCTGTTTCAGGG-3′, REVERSE 5′-CAGGACAGATTCAGTGGATCTTTCAGCTACGCAACCCATCAG-3′. The gene insert was amplified from GFP-DRD2 (addgene #24099) linearized with the following primers: FORWARD 5′-GATGGGTTGCGTAGCTGAAAGATCCACTGAATCTGTCCTGGTATGA-3′, REVERSE 5′-CCCTGAAACAGCACCTCAAGGCAGTGGAGGATCTTCAGGAAGG-3′. Linearized fragments were assembled via Gibson assembly.

### 4.3 Cloning of Recombinant Gqi5

A human codon-optimized DNA fragment of the Gqi5 chimera was synthesized (IDT Technologies) and amplified from the following primers: FORWARD 5′-AGCTGTACCCGGTCGCAATGACCCTGGAGAGCATCATGG-3′, REVERSE 5′-TGTGCGGGCAGGCAGAGTCAGAACAGGCCGCAGTCC-3′. A pcDNA3.1 vector was derived from GFP-DRD2 (Addgene #24099) linearized with the following primers: FORWARD 5′-AGGACTGCGGCCTGTTCTGACTCTGCTGCCTGCCCG-3′. REVERSE: CCAGGGTCATGGTGGCGACCGGG. Linearized fragments were assembled via Gibson assembly.

### 4.4 Stable Cell Line Generation

A stable HEK293T-derived cell line-expressing DRD2-Strep Tag II fusion was generated via lentiviral transduction following the method of Elegheert et al.^76^ The initial expansion of the polyclonally transduced cells was enriched for the top 10% of expressing cells via fluorescence-assisted cell sorting (BD FACS Aria III). Expression levels remained high for >90% of the population after 15 passages.

### 4.5 Intracellular Ca^2+^ Mobilization Assay

A stable HEK293T-derived cell line-expressing DRD2-Strep Tag II fusion was maintained in DMEM supplemented with 10% FBS, 100 U/mL penicillin, and 100 μg/mL streptomycin in a humidified atmosphere at 37 °C in 5% CO_2_. On day 1, cells were plated at a density of 4 × 10^4^ cells/cm^2^ in a poly-d-lysine-coated 18-well chambered coverslip (Ibidi). The following day (day 2), cells were transfected with a 10× solution of 3:1 mixture of Gqi5/Optifect Transfection Reagent (Thermo) in unsupplemented DMEM. On day 3, the transfection media was removed and calcium-sensitive dye loading was performed following the protocol of the Fluo-4 Direct Calcium Assay Kit (Invitrogen). 5× drug stimulation solutions were prepared in filter-sterilized HBSS. Once Fluo-4 loading was complete, a time series acquisition at a rate of 1 fps was recorded using a Zeiss LSM 980 with Airyscan 2. Basal fluorescence was recorded for 20 s, followed by the addition of drug solution to a 1× final concentration and acquisition for an additional 40 s. Results in the form of fold fluorescence increase over basal were averaged over 50 cells in ImageJ (NIH), and GraphPad Prism was used for the analysis of data.

### 4.6 β Arrestin Assay

HTLA cells were a gift from the laboratory of G. Barnea and were maintained in DMEM supplemented with 10% FBS, 100 U/mL penicillin, 100 μg/mL streptomycin, 2 μg/mL puromycin, 100 μg/mL hygromycin B, and 100 μg/mL G418 in a humidified atmosphere at 37 °C in 5% CO_2_. On day 1, cells were plated at a density of 1 × 10^5^ cells/cm^2^ in a black wall, clear-bottom 96-well plate (Nunc). The following day (day 2), cells were transfected with a 10× solution of 3:1 mixture of DRD2-TANGO/Optifect Transfection Reagent (Thermo) in unsupplemented DMEM. On day 3, 1× drug stimulation solutions were prepared in filter-sterilized unsupplemented DMEM. The transfection media was shaken or aspirated from the wells, and drug stimulation solutions were gently added. On day 4, drug solutions were removed from one well every 10 s (to maintain consistency of incubation time) and 50 μL per well of Bright-Glo solution (Promega) diluted 20-fold in HBSS was added. After incubation for 2 min at room temperature, luminescence was counted with an integration time of 10 s in a Spectramax i3x plate reader (Molecular Devices).

### 4.7 Photo-Cross-Linking of DRD2-Expressing 293T Cells and Primary Neurons with Probes

For all irradiation experiments, a Chemglass Biogrow CLS-1625 UV Lamp (New Jersey) was used and set to an irradiation wavelength of 365 nm, with a lamp power of 6 W. All samples were irradiated for 30 min at approximately 2 cm from the lamp.

### 4.8 Click Chemistry Conjugation and Microscopy

HEK293T cells and a stable HEK293T-derived cell line-expressing DRD2-Strep Tag II fusion were maintained in DMEM supplemented with 10% FBS, 100 U/mL penicillin, and 100 μg/mL streptomycin in a humidified atmosphere at 37 °C in 5% CO_2_. Cells were plated at a density of 4 × 10^4^ cells/cm^2^ in a poly-d-lysine-coated 18-well chambered coverslip (Ibidi) and allowed to grow to a 70% confluency. Probe solutions at a concentration of 5 μM were prepared in sterile-filtered HBSS. Media was removed from the wells, and probe solutions were added and allowed to incubate for 2 min. The probe solutions were removed, and the cells were washed twice with HBSS and irradiated with a 365 nm UV lamp (8 W, 2 cm distance) for 30 min at room temperature. The HBSS was removed, and the cells were fixed in 4% paraformaldehyde solution in PBS (15 min at room temperature), washed twice with PBS, and permeabilized in 0.2% Tween-20 in PBS for 20 min at room temperature. Permeabilized cells were then blocked for 1 h at room temperature, then treated with freshly premixed Click-iT Kit (Invitrogen) reaction buffer (1× reaction buffer, 5 μM AF555 picolyl azide (AAT Bioquest), 100:0 CuSO_4_/copper protectant, 1× reaction buffer additive), and incubated in the dark with constant shaking for 30 min at room temperature. The cells were then washed 3 times with TBS with 3% BSA and incubated with 1 μg/mL Anti-Strep Tag II rabbit polyclonal antibody (Abcam, ab76949) in TBS with 3% BSA overnight at 4 °C. The primary antibody solution was then removed, and the cells were washed 3 times with a 5 min TBS incubation. The cells were then incubated with a 0.1 μg/mL Alexa Fluor 488 conjugated goat antirabbit IgG (Abcam, ab150077) in TBS with 3% BSA for 1 h at room temperature with constant shaking. The cells were then washed 3 times with TBS and imaged for Alexa Fluor 488 fluorescence and AF555 fluorescence using a Zeiss LSM 980 with Airyscan 2. Images were processed using Fiji (NIH).

### 4.9 Western Blot

A stable HEK293T-derived cell line-expressing DRD2-Strep Tag II fusion was maintained in DMEM supplemented with 10% FBS, 100 U/mL penicillin, and 100 μg/mL streptomycin in a humidified atmosphere at 37 °C in 5% CO_2_. Cells were plated at a density of 4 × 10^4^ cells/cm^2^ in T300 tissue culture flasks (CellTreat) and allowed to grow to an 80% confluency. At confluency, media was removed and cells were washed once with cold HBSS, followed by incubation for 15 min in cold HBSS with 0.53 mM EDTA. Following incubation, cells were scraped off of the bottom of the flask and the cell suspension was transferred to a 15 mL conical tube and then pelleted at 500*g* for 5 min. The supernatant was removed, and the cell pellet was resuspended in HBSS followed by centrifugation at 500*g* for 5 min. Probe solutions (**5**, **7**, and **16**) at a concentration of 100 nM and competitor (ropinirole or pramipexole) at a concentration of 50 μM were prepared in both sterile-filtered HBSS. The supernatant of the cell pellet was removed, and the cells were resuspended in probe solution and incubated at room temperature for 2 min followed by centrifugation at 500*g* for 5 min. The cell pellet was resuspended in either HBSS or 50 μM competitor solution in HBSS and irradiated with a 365 nm UV lamp (8 W, 2 cm distance) for 30 min at room temperature. The cells were then pelleted at 500*g* for 5 min, and the supernatant was removed. Membrane fractionation was performed following the MEM-PER Membrane Protein Extraction Kit (Thermo). The solubilized membrane fraction was incubated with hydrophilic streptavidin magnetic beads (NEB) for 1 h at room temperature. The flow-through was removed, and the beads were washed twice with PBS. The beads were then treated with freshly premixed Click-iT Kit (Invitrogen) reaction buffer (1× reaction buffer, 5 μM AF555 picolyl azide (AAT Bioquest), 100:0 CuSO_4_:Copper Protectant, 1× reaction buffer additive) and incubated in the dark with constant shaking for 30 min at room temperature. The captured proteins were eluted by incubation in 6 M urea SDS-PAGE loading buffer (6 M urea, 200 mM tris, 4% SDS, 20% glycerol, 2% β-mercaptoethanol, 20 mM EDTA, 0.04% bromophenol blue, pH 7.4) at 65 °C for 10 min. Samples were then separated via SDS-PAGE using a NuPAGE 4–12% bis–tris precast gel (Invitrogen). The gel was then transferred to a PVDF membrane via an iBlot 2 gel transfer device, and the membrane was blocked for 1 h at room temperature in TBST with 5% BSA. Following blocking, the membrane was incubated with either 1 μg/mL Anti-Strep Tag II rabbit polyclonal antibody (Abcam, ab76949) or 2 μg/mL Anti-DRD2 rabbit polyclonal antibody (AbClonal, A12930) in TBST with 5% BSA overnight at 4 °C. The primary antibody solution was removed, and the membrane was washed 3 times with a 5 min TBST incubation. The membrane was then incubated with 0.1 μg/mL IRdye680RD conjugated goat antirabbit IgG (Abcam, ab216777) in TBST with 5% BSA for 1 h at room temperature with constant shaking. The membrane was washed 3 times with TBST and imaged using an Azure Sapphire Biomolecular Imager. Images were processed using Fiji (NIH).

### 4.10 Photoaffinity Quantification Using FACS

HEK293T cells and a stable HEK293T-derived cell line-expressing DRD2-Strep Tag II fusion were maintained in DMEM supplemented with 10% FBS, 100 U/mL penicillin, and 100 μg/mL streptomycin in a humidified atmosphere at 37 °C in 5% CO_2_. Cells were plated at a density of 4 × 10^4^ cells/cm^2^ in T75 tissue culture flasks (CellTreat) and allowed to grow to an 80% confluency. At confluency, media was removed and cells were washed once with cold HBSS, followed by incubation for 15 min in cold HBSS with 0.53 mM EDTA. Following incubation, cells were scraped off of the bottom of the flask and the cell suspension was transferred to a 15 mL conical tube and then pelleted at 500*g* for 5 min. The supernatant was removed, and the cell pellet was resuspended in HBSS followed by centrifugation at 500*g* for 5 min. Probe solutions at a concentration of 100 nM were prepared in sterile-filtered HBSS. The supernatant of the cell pellet was removed, and the cells were resuspended in probe solution and incubated at room temperature for 2 min followed by centrifugation at 500*g* for 5 min. The cell pellet was then washed twice with HBSS and irradiated with a 365 nm UV lamp (8 W, 2 cm distance) for 30 min at room temperature. The cells were then pelleted at 500*g* for 5 min, the supernatant was removed, and the cell pellet was fixed in 4% paraformaldehyde solution in PBS (15 min at room temperature), washed twice with PBS, and permeabilized in 0.2% Tween-20 in PBS for 20 min at room temperature. Permeabilized cells were then incubated in PBS with 5% BSA for 1 h at room temperature and then treated with freshly premixed Click-iT Kit (Invitrogen) reaction buffer (1× reaction buffer, 5 μM AF555 picolyl azide (AAT Bioquest), 100:0 CuSO_4_:Copper Protectant, 1× reaction buffer additive) and incubated in the dark with constant shaking for 30 min at room temperature. The cells were then washed 3 times with PBS with 5% BSA and analyzed with a BD Accuri C6 flow cytometer. Samples were gated on forward scatter and side scatter to exclude cell debris and aggregates, and red channel fluorescence was analyzed for the percent of events with increased fluorescence over basal.

### 4.11 LC/MS Sample Prep of DRD2 Stable Cell Line with Probe 5 or 7

HEK293T cells and a stable HEK293T-derived cell line-expressing DRD2-Strep Tag II fusion were maintained in DMEM supplemented with 10% FBS, 100 U/mL penicillin, and 100 μg/mL streptomycin in a humidified atmosphere at 37 °C in 5% CO_2_. Cells were plated at a density of 4 × 10^4^ cells/cm^2^ in T300 tissue culture flasks (CellTreat) and allowed to grow to an 80% confluency. At confluency, media was removed, and cells were washed once with cold HBSS, followed by incubation for 15 min in cold HBSS. Following incubation, cells were scraped off of the bottom of the flask and the cell suspension was transferred to a 50 mL conical tube and then pelleted at 500*g* for 5 min. Solutions containing a 30 μM probe were prepared in sterile-filtered HBSS. The supernatant of the cell pellet was removed, and the cells were resuspended in probe solution and incubated at room temperature for 2 min followed by centrifugation at 500*g* for 5 min. The cell pellet was then resuspended in HBSS and irradiated with a 365 nm UV lamp (8 W, 2 cm distance) for 30 min at room temperature. The cells were then pelleted at 500*g* for 5 min, and the supernatant was removed. Membrane fractionation was performed following the MEM-PER Membrane Protein Extraction Kit (Thermo). The solubilized membrane fraction was incubated with hydrophilic streptavidin magnetic beads (NEB) for 1 h at room temperature. The flow-through was removed, and the beads were washed twice with PBS.

### 4.12 Rat Whole Brain Photo-Cross-Linking

To prepare the homogenate, adult rat brain tissue was microdissected and the olfactory bulb was discarded and sliced into 1 mm pieces. The pieces were suspended in a phosphate-based NP-40 lysis buffer (150 mM NaCl, 50 mM phosphate, 1% NP-40, pH 8.0), homogenized with a handheld homogenizer (IKA T-18 digital homogenizer), and incubated on ice for 30 min. The homogenate was then cleared via centrifugation (16,000*g*, 20 min), and the supernatant was reserved. The total protein concentration was determined via BCA assay (Pierce) and adjusted to 6 mg/mL. Probe solutions at a concentration of 5 mM were prepared in sterile-filtered HBSS and were added to protein solutions to a final concentration of 50 μM. Samples were inverted 3× to mix and irradiated with a 365 nm UV lamp (8 W, 2 cm distance) for 30 min at room temperature. Samples were then buffer-swapped (10k MWCO, Pierce) into fresh phosphate-based NP-40 lysis buffer and freshly premixed Click-iT Kit (Invitrogen) reaction buffer (1× reaction buffer, 50 μM PC biotin azide (Click Chemistry Tools), 70:30 CuSO_4_/copper protectant, 1× reaction buffer additive) and incubated in the dark with constant shaking for 30 min at room temperature. Samples were then buffer-swapped (10k MWCO, Pierce) into fresh phosphate-based NP-40 lysis buffer and incubated with hydrophilic streptavidin magnetic beads (NEB) for 1 h at room temperature. The flow-through was removed, and the beads were washed twice with PBS. The protein was then eluted into PBS (150 mM NaCl, 20 mM phosphate, pH 8.0) by 365 nm irradiation (8 W, 2 cm distance) and submitted for LC-MS processing.

### 4.13 Sample Processing for Mass Spectrometry

Samples were reduced and alkylated by sequentially adding TCEP and iodoacetamide to final concentrations of 5 and 10 mM, respectively. The reaction was allowed to proceed in the dark for 25 min. Samples were digested with 125 ng of trypsin gold (Promega), overnight at 37 °C. The following day, samples were acidified using trifluoroacetic acid (TFA, Sigma-Aldrich) to pH ≤ 3 and desalted using 2-core MCX stage tips (3M, 2241).^77^ The stage tips were activated with ACN followed by 3% ACN with 0.1% TFA. Next, samples were applied, followed by two washes with 3% ACN with 0.1% TFA and one wash with 65% ACN with 0.1% TFA. Peptides were eluted with 75 μL of 65% ACN with 5% NH_4_OH (Sigma-Aldrich) and dried.

### 4.14 LC/MS Methods

Samples were dissolved in 20 μL of water containing 2% acetonitrile and 0.5% formic acid, and 5 μL was diluted with 25 μL in a sample vial. Of this solution, 2 μL was injected onto a pulled tip nano-LC column with 75 μm inner diameter packed to 25 cm with 3 μm, 120 Å, C18AQ particles (Dr. Maisch). The peptides were separated using a 60 min gradient from 3 to 28% acetonitrile, followed by a 7 min ramp to 85% acetonitrile and a 3 min hold at 85% acetonitrile. The column was connected inline with an Orbitrap Lumos via a nanoelectrospray source operating at 2.2 kV. The mass spectrometer was operated in data-dependent top speed mode with a cycle time of 2.5 s. MS^1^ scans were collected at a 120,000 resolution with a maximum injection time of 50 ms. Dynamic exclusion was applied for 15 s. HCD fragmentation was used followed by MS^2^ scans in the ion trap with a 35 ms maximum injection time.

### 4.15 Database Searching and Label-Free Quantification

The MS data was searched using SequestHT in Proteome Discoverer (version 2.4, Thermo Scientific) against a human protein database (Uniprot, containing 20392 reviewed entries, retrieved 5/27/2021) and a list of common laboratory contaminant proteins (Thermo Scientific, 298 entries, 2015). Enzyme specificity for trypsin was set to semitryptic with up to two missed cleavages. Precursor and product ion mass tolerances were 10 ppm and 0.6 Da, respectively. Cysteine carbamidomethylation was set as a fixed modification. Methionine oxidation, protein N-terminal acetylation, and the mass of the appropriate photoaffinity tag, allowed on all 20 proteogenic amino acids, were set as variable modification. The output was filtered using the Percolator algorithm with a strict FDR set to 0.01.

## Supporting Information Available

General chemistry procedures; Western blots; bioinformatic pathway lists and local network analyses; and NMR spectra (PDF)
Primary mass spec peak lists, peptide sequence, and protein ID and ID confidence for probe 5 (ELSX)
Primary mass spec peak lists, peptide sequence, and protein ID and ID confidence for probe 7 (XLSX)
