# Supplementary material for: Developing Photoaffinity Probes for Dopamine Receptor D2 to Determine Targets of Parkinson’s Disease Drugs
Source: ACS Chem Neurosci. 2022 Oct 2;13(20):3008–22. doi: 10.1021/acschemneuro.2c00544 (PMC9585581; doi:10.1021/acschemneuro.2c00544)
Supplement: Supplementary file 1 — cn2c00544_si_001.pdf [file cn2c00544_si_001.pdf]

Supporting Information

Developing Photoaffinity Probes for Dopamine Receptor D2 to Determine Targets of Parkinson's Disease Drugs

Spencer T. Kim[1], Emma J. Doukmak[1], Raymond G. Flax[1], Dylan J. Gray[1], Victoria N. Zirimu[1], Ebbing de Jong[3], Rachel C. Steinhardt[1,2,4]

Table of Contents

Synthetic Chemistry General experimental procedures 3
HPLC Purification of Probe 4 4
Figure S1. Western blots for probes 5, 7, and 16. 5
Figure S2. Previously-known proteins modulated by ropinirole according to STICH 6
Table S1. Previously known KEGG pathways for Ropinirole 7
Figure S3. Previously-known proteins modulated by pramipexole according to STICH 8
Table S2. Previously known KEGG pathways for pramipexole 9
Table S3. KEGG Pathway Analysis for Hits in Common for Probes 5 and 7. 10
Table S4. STRING Network Statistics for Hits in Common for Probes 5 and 7 11
Table S5. WikiPathways Hits for probes 5 and 7. 12
Table S6. Local network analysis (via STRING) for hits in common for probes 5 and 7 14
Table S7. STRING Network statistics for probe 5 15
Table S8. KEGG Analysis of hit 5 16
Table S9. STRING Network statistics for probe 7 19
Table S10. Molecular Function (Gene Ontology) Analysis for probe 7 20
NMR Spectra 21

Synthetic Chemistry General experimental procedures

All reactions were performed in flame- or oven-dried glassware under positive pressure of nitrogen or argon unless otherwise noted. Dichloromethane, dimethylacetamide, *N*,*N*-dimethylformamide, triethylamine, and toluene were dried by columns packed with activated alumina on a solvent purification system. Anhydrous pyridine and DMSO were purchased from Acros Organics in AcroSeal bottles. All reagents were used as purchased without further purification. Thin layer chromatography (TLC) was performed on Merck 60 F_{254} pre-coated silica gel plates, and plates were visualized with UV light and ninhydrin stain when appropriate. Flash-column chromatography was performed using silica gel (60 Å, 230-240 mesh, Merck KGA). HPLC runs were conducted on a Waters 2545 binary gradient pump equipped with UV-Vis Detector. Analytical runs were performed using a Phenomenex C18 column (4.6 x 50 mm). Separations were monitored at 254 nm. NMR spectra were recorded with Bruker Avance spectrometers using deuterated solvents. ^{1}H NMR spectra were recorded at 500 MHz as indicated. ^{13}C spectra were recorded at 125 MHz. ^{1}H NMR data are reported in the following order: chemical shift (δ ppm), multiplicity, coupling constant (Hz), and integration. ^{13}C NMR data are reported in terms of chemical shift. Infrared spectra were recorded using a Thermo Scientific iD5 ATR infrared spectrophotometer. High-resolution mass spectra were obtained from the University of Delaware Mass Spectrometry Facility. The abbreviations used can be found in the document *JOC Standard Abbreviations and Acronyms*.

HPLC Purification of Probe 4

Probe 4 was purified on a Waters HPLC, with a Waters 2545 binary gradient pump equipped with Waters 2489 UV-Vis Detector. The column was a Sunfire 5 um silica prep 4.6 x 250 mm, 100% ACN, 2 mL/min.

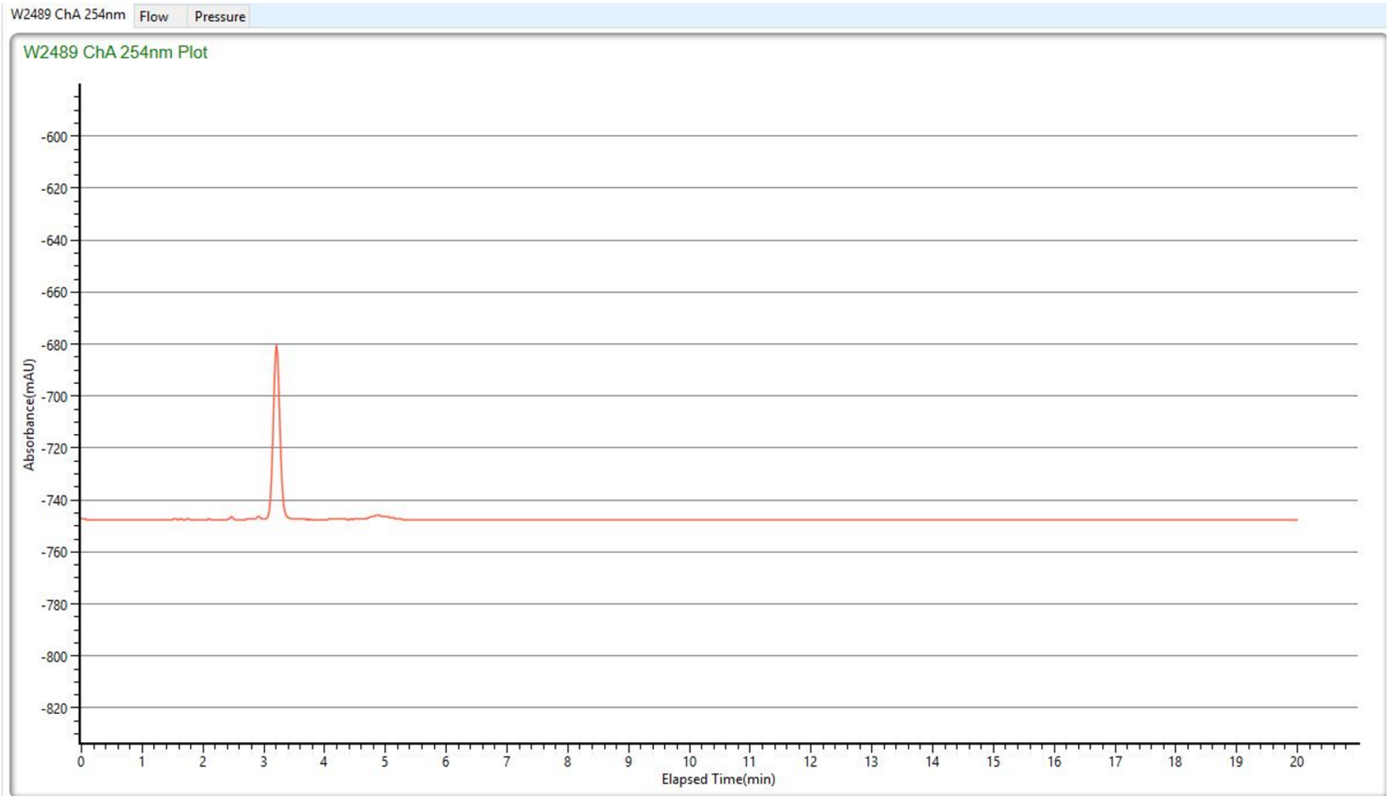

Figure S1. Western blots for probes 5, 7, and 16.

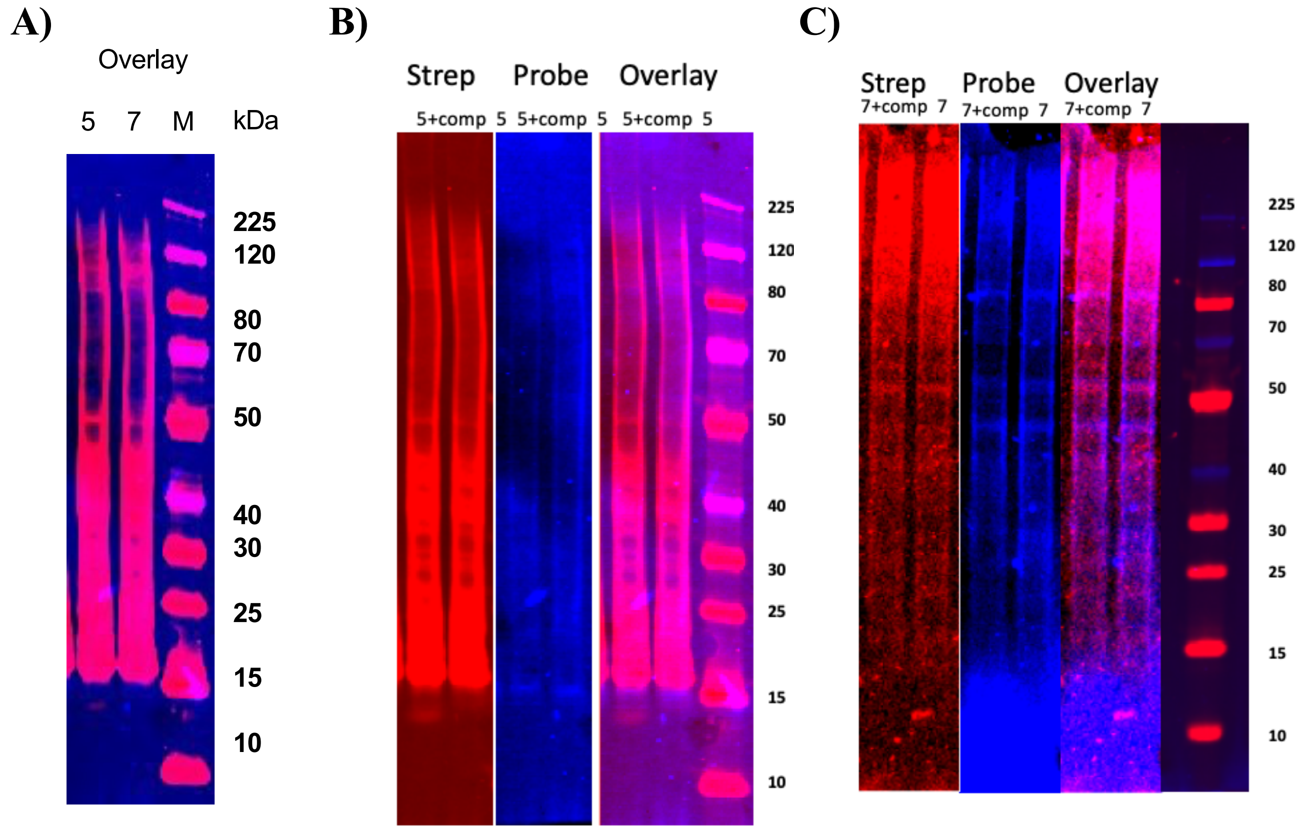

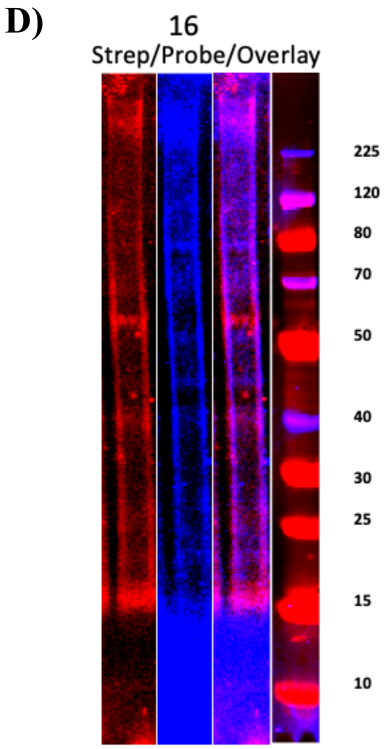

Figure S1. (A) Overlaid gels with strep tag II (DRD2) and probe channels, the band at ~50 kDA corresponds to DRD2. Lanes: 5 is probe 5, 7 is probe 7, M is marker. **(B)** Competition assay with probe **5. (C)** Competition assay with probe **7. (D)** Negative control probe **16**

Figure S2. Previously-known proteins modulated by ropinirole according to STICH

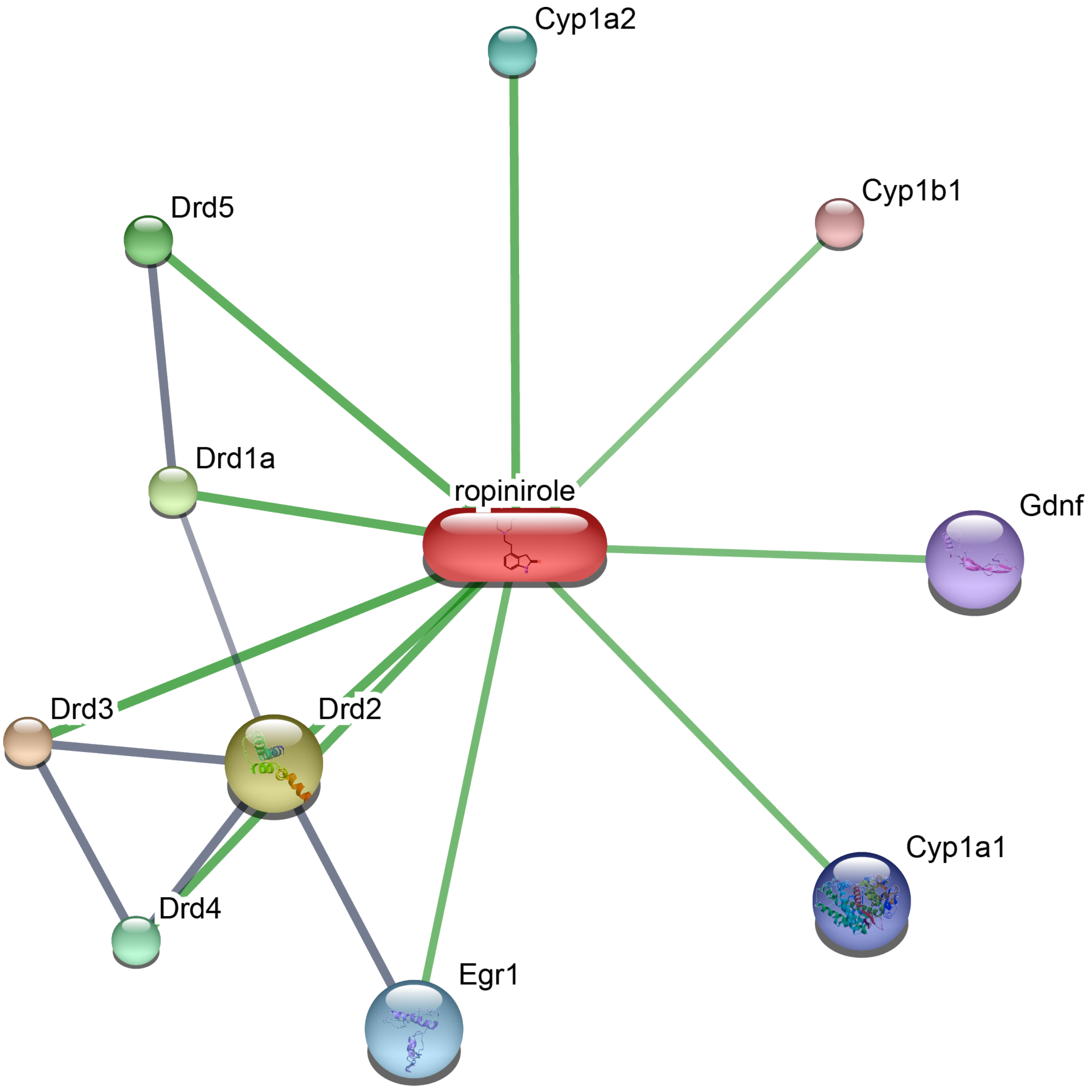

Figure S2. Stronger associations are represented by thicker lines. Protein-protein interactions are shown in grey, chemical-protein interactions in green. Image generated using STICH database and user interface, stich.embl.de.

Table S1. Previously known KEGG pathways for Ropinirole

**pathway description**	**observed gene count**	**false discovery rate**	**matching proteins in network (labels)**
Dopaminergic synapse	5	4.80E-07	Drd1a,Drd2,Drd3,Drd4,Drd5
Neuroactive ligand-receptor interaction	5	1.50E-05	Drd1a,Drd2,Drd3,Drd4,Drd5
Tryptophan metabolism	2	0.0145	Cyp1a1,Cyp1a2
Cocaine addiction	2	0.0145	Drd1a,Drd2
Metabolism of xenobiotics by cytochrome P450	2	0.018	Cyp1a1,Cyp1a2
Steroid hormone biosynthesis	2	0.0195	Cyp1a1,Cyp1a2
Retinol metabolism	2	0.0195	Cyp1a1,Cyp1a2
Chemical carcinogenesis	2	0.0197	Cyp1a1,Cyp1a2
Gap junction	2	0.0204	Drd1a,Drd2
Alcoholism	2	0.046	Drd1a,Drd2

Figure S3. Previously-known proteins modulated by pramipexole according to STICH

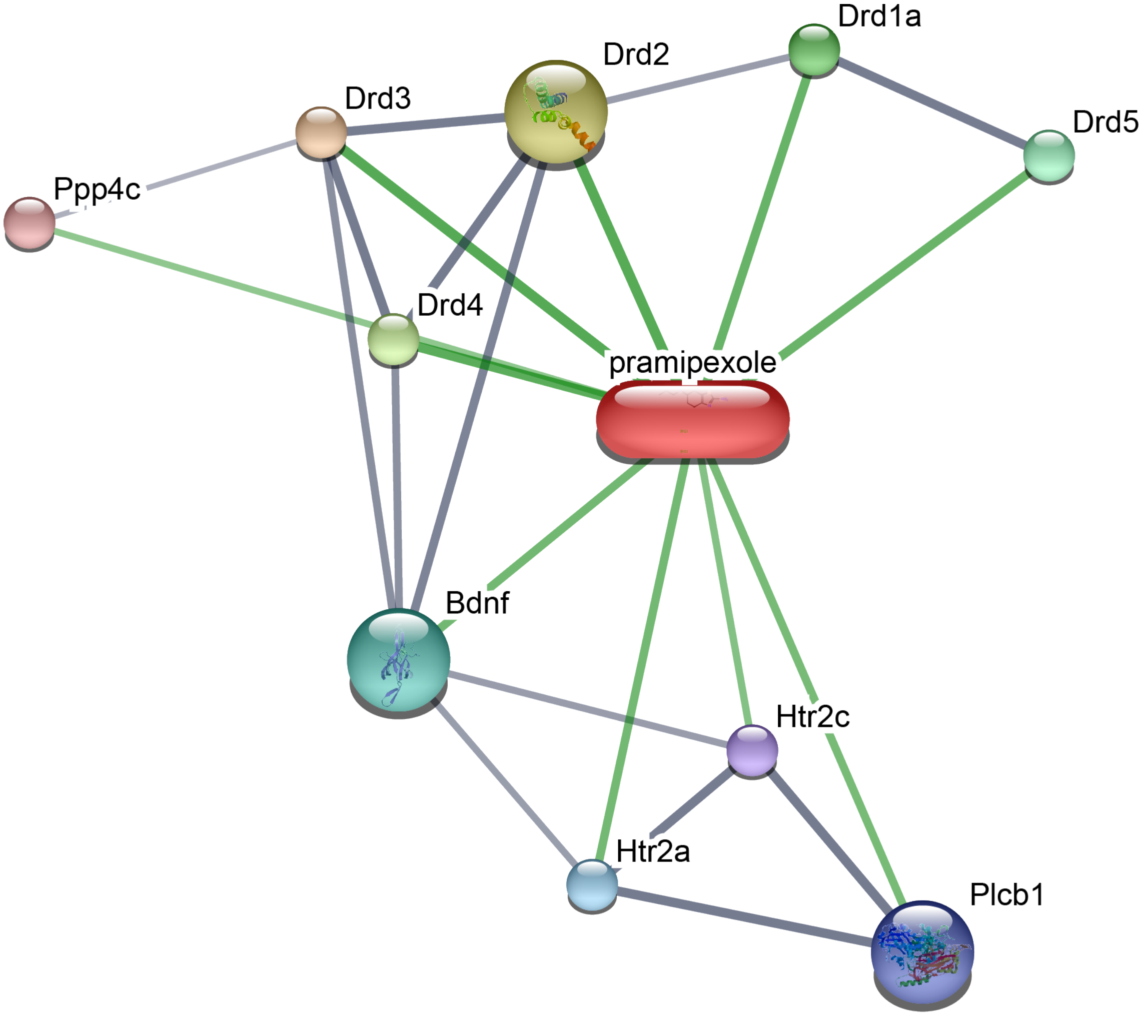

Figure S3. Stronger associations are represented by thicker lines. Protein-protein interactions are shown in grey, chemical-protein interactions in green. Image generated using STICH database and user interface, stich.embl.de.

Table S2. Previously known KEGG pathways for pramipexole.

pathway description	observed gene count	false discovery rate	matching proteins in network (labels)
Neuroactive ligand-receptor interaction	7	3.04E-09	Drd1a,Drd2,Drd3,Drd4,Drd5,Htr2a,Htr2c
Dopaminergic synapse	5	2.40E-07	Drd1a,Drd2,Drd3,Drd4,Drd5
Gap junction	4	4.20E-06	Drd1a,Drd2,Htr2a,Htr2c
Calcium signaling pathway	4	6.54E-05	Drd1a,Drd5,Htr2a,Htr2c
Cocaine addiction	3	6.54E-05	Bdnf,Drd1a,Drd2
Alcoholism	3	0.00125	Bdnf,Drd1a,Drd2
Inflammatory mediator regulation of TRP channels	2	0.0434	Htr2a,Htr2c
Serotonergic synapse	2	0.0464	Htr2a,Htr2c

Table S3. KEGG Pathway Analysis for Hits in Common for Probes 5 and 7.

term description	observed gene count	background gene count	strength	false discovery rate	matching proteins in network (labels)
Alzheimer disease	8	355	0.94	0.0011	PSMA4,PPIF,ATP5E,CYCS, TUBB, PSMD4,ATP5F1,NOS1
Parkinson disease	7	240	1.05	0.0011	PSMA4,PPIF,ATP5E,CYCS, TUBB,PSMD4,ATP5F1
Huntington disease	7	298	0.95	0.0011	PSMA4,PPIF,ATP5E,CYCS, TUBB,PSMD4,ATP5F1
Prion disease	7	265	1.01	0.0011	PSMA4,PPIF,ATP5E,CYCS, TUBB,PSMD4,ATP5F1
Amyotrophic lateral sclerosis	7	352	0.88	0.0025	PSMA4,ATP5E,CYCS, TUBB,PSMD4,ATP5F1, NOS1
Spinocerebellar ataxia	4	135	1.06	0.0261	PSMA4,PPIF,CYCS,PSMD4

Table S4. STRING Network Statistics for Hits in Common for Probes 5 and 7

number of nodes:
101
number of edges:
673
average node degree:
13.3
avg. local clustering coefficient:
0.548
expected number of edges:
240
PPI enrichment p-value:
< 1.0e-16

Table S5. WikiPathways Hits for probes 5 and 7.

term description	observed gene count	background gene count	strength	false discovery rate	matching proteins in your network (labels)
Proteasome degradation	17	60	1.74	7.58E-21	PSMA4,PSMC4, PSMD5,PSMD8, PSMA3,PSMD7, PSMA2,PSMB7, PSMC1,PSMA6, PSMD11,PSMB1, PSMD3,PSMB6, PSMA5,PSMB4, PSMD4
Alzheimers disease and miRNA effects	20	258	1.18	3.36E-15	PSMA4,PSMC4, PSMD8,PSMA3, PSMD7,PSMA2, PPIF,PSMB7, PSMC1, PSMA6,PSMB1, PSMD3,VDAC1, PSMB6,PSMA5, PSMB4,CYCS, TUBB,PSMD4, NOS1
Alzheimers disease	20	255	1.18	3.36E-15	PSMA4,PSMC4, PSMD8,PSMA3, PSMD7,PSMA2, PPIF,PSMB7, PSMC1,PSMA6, PSMB1,PSMD3, VDAC1,PSMB6, PSMA5,PSMB4, CYCS,TUBB, PSMD4,NOS1
Cytoplasmic ribosomal proteins	13	86	1.47	5.67E-13	RPL18A,RPL19, RPS12,RPS16, RPL35,RPL8, RPS11,RPS27A, RPL37,RPS3, RPS23,RPS9, RPS21
Oxidative phosphorylation	10	60	1.51	3.37E-10	ATP5D,ATP5E, ATP5B,ATP5G3,

					ATP5O,ATP5J2, ATP5L,ATP5H, ATP5I,ATP5F1
Electron transport chain: OXPHOS system in mitochondria	11	103	1.32	1.76E-09	ATP5D,ATP5E, ATP5B,ATP5G3, ATP5O,ATP5J2, ATP5L,ATP5H, ATP5I,UQCRFS1, ATP5F1
Parkin-ubiquitin proteasomal system pathway	9	67	1.42	1.79E-08	PSMC4,PSMD5, PSMD8, PSMD7, PSMC1,PSMD11, PSMD3,TUBB, PSMD4
7q11.23 copy number variation syndrome	6	104	1.05	0.0019	ATP5D,ATP5E, ATP5B, ATP5G3,ATP5O, ATP5F1
mRNA processing	6	125	0.97	0.0044	SNRPD3,SNRPA, SNRPA1, SNRPF,SNRPG, HNRNPD
VEGFA-VEGFR2 signaling pathway	10	428	0.66	0.0058	RPL18A,FXR2, SDCBP, PSMD11,RPS11, FARSB,GJA1, CYCS,PSMD4, EWSR1

Table S6. Local network analysis (via STRING) for hits in common for probes 5 and 7

term description	observed gene count	background gene count	strength	false discovery rate
Proteasome	17	46	1.85	1.11E-21
Proteasome	16	35	1.95	1.11E-21
Regulation of ornithine decarboxylase (ODC), and RAS signaling downstream of NF1 loss-of-function variants	18	74	1.67	4.93E-21
Proteasome	13	26	1.99	2.57E-18
Formation of ATP by chemiosmotic coupling	10	15	2.11	9.38E-15
Proteasome subunit, and proteasome regulatory particle	10	21	1.96	1.06E-13
Cytoplasmic ribosomal proteins	13	75	1.53	2.06E-13
Cytoplasmic ribosomal proteins	12	70	1.52	2.40E-12
Formation of ATP by chemiosmotic coupling	8	10	2.19	3.16E-12
Proteasome regulatory particle, and proteasome subunit	8	16	1.99	4.75E-11
Oxidative phosphorylation	11	103	1.32	2.62E-09
Proteasome regulatory particle, and proteasome alpha-type subunit	6	11	2.02	2.83E-08
Cytoplasmic ribosomal proteins	8	57	1.43	2.06E-07
Cytoplasmic ribosomal proteins	7	47	1.46	1.53E-06
Formation of ATP by chemiosmotic coupling	4	5	2.19	1.08E-05
Cytosolic small ribosomal subunit, and ribosomal_l31e	4	13	1.78	0.00019
Proteasome regulatory particle, lid subcomplex, and 26s proteasome non-atpase regulatory subunit 7/8	3	5	2.07	0.00091
U2-type precatalytic spliceosome, and U1 snRNP	5	60	1.21	0.0026

Table S7. STRING Network statistics for probe 5

number of nodes:
119
number of edges:
437
average node degree:
7.34
avg. local clustering coefficient:
0.468
expected number of edges:
181
PPI enrichment p-value:
< 1.0e-16

Table S8. KEGG Analysis of hit 5

term description	observed gene count	strength	false discovery rate	matching proteins in your network (labels)
Parkinson disease	31	1.33	1.49E-28	PSMA4,PSMC4,NDUFB4, UQCRC1,PSMD8,NDUFB7, PSMA3,PSMD7,PSMA2, NDUFS7,NDUFB3,NDUFA2, NDUFA10,NDUFB5,PSMB7, PSMC1,PSMA6,PSMD11, PSMB1,NDUFS3,PSMD3, NDUFA9,UQCRC2,NDUFB10, PSMB6,PSMA5,RPS27A, CYC1,PSMA7,SEPT5, NDUFA6
Prion disease	29	1.26	6.03E-25	PSMA4,PSMC4,NDUFB4, UQCRC1,PSMD8,NDUFB7, PSMA3,PSMD7,PSMA2, NDUFS7,NDUFB3,NDUFA2, NDUFA10,NDUFB5,PSMB7, PSMC1,PSMA6,PSMD11, PSMB1,NDUFS3,PSMD3, NDUFA9,UQCRC2,NDUFB10, PSMB6,PSMA5,CYC1, PSMA7,NDUFA6
Alzheimer disease	31	1.16	2.99E-24	PSMA4,PSMC4,NDUFB4, UQCRC1,PSMD8,NDUFB7, PSMA3,PSMD7,PSMA2, NDUFS7,NDUFB3,NDUFA2, NDUFA10,NDUFB5,PSMB7, PSMC1,PSMA6,PSMD11, PSMB1,NDUFS3,PSMD3, NDUFA9,UQCRC2,NDUFB10, PSMB6,PSMA5,CHRM1, CYC1,PSMA7,RTN3,NDUFA6
Huntington disease	29	1.2	6.60E-24	PSMA4,PSMC4,NDUFB4, UQCRC1,PSMD8,NDUFB7, PSMA3,PSMD7,PSMA2, NDUFS7,NDUFB3,NDUFA2, NDUFA10,NDUFB5,PSMB7, PSMC1,PSMA6,PSMD11, PSMB1,NDUFS3,PSMD3, NDUFA9,UQCRC2,NDUFB10, PSMB6,PSMA5,CYC1, PSMA7,NDUFA6

Amyotrophic lateral sclerosis	29	1.13	4.20E-22	PSMA4,PSMC4,NDUFB4, UQCRC1,PSMD8,NDUFB7, PSMA3,PSMD7,PSMA2, NDUFS7,NDUFB3,NDUFA2, NDUFA10,NDUFB5,PSMB7, SMC1,PSMA6,PSMD11,PSMB1, NDUFS3,PSMD3,NDUFA9, UQCRC2,NDUFB10,PSMB6, PSMA5,CYC1,PSMA7, NDUFA6
Proteasome	16	1.79	5.53E-21	PSMA4,PSMC4,PSMD8, PSMA3,PSMD7,PSMA2,P SMB7,PSMC1,PSMA6, PSMD11,PSMB1,PSMD3, PSMB6,PSMA5,PSME3, PSMA7
Spinocerebellar ataxia	15	1.26	8.39E-13	PSMA4,PSMC4,PSMD8, PSMA3,PSMD7,PSMA2, PSMB7,PSMC1,PSMA6, PSMD11,PSMB1,PSMD3, PSMB6,PSMA5,PSMA7
Oxidative phosphorylation	14	1.25	8.52E-12	NDUFB4,UQCRC1,NDUFB7, NDUFS7,NDUFB3,NDUFA2, NDUFA10,NDUFB5,NDUFS3, NDUFA9,UQCRC2,NDUFB10, CYC1,NDUFA6
Non-alcoholic fatty liver disease	14	1.19	3.88E-11	NDUFB4,UQCRC1,NDUFB7, NDUFS7,NDUFB3,NDUFA2, NDUFA10,NDUFB5,NDUFS3, NDUFA9,UQCRC2,NDUFB10, CYC1,NDUFA6
Retrograde endocannabinoid signaling	12	1.13	6.63E-09	NDUFB4,NDUFB7,NDUFS7, NDUFB3,NDUFA2,NDUFA10, NDUFB5,NDUFS3,NDUFA9, NDUFB10,GNG12,NDUFA6
Thermogenesis	14	1	7.51E-09	NDUFB4,UQCRC1,NDUFB7, NDUFS7,NDUFB3,NDUFA2, NDUFA10,NDUFB5,NDUFS3 ,NDUFA9,UQCRC2,NDUFB10, CYC1,NDUFA6
Ribosome	8	1.01	5.43E-05	RPL18A,RPL19,RPS12, RPS16,RPL35,RPL8, RPS11,RPS27A
Endocytosis	9	0.79	0.00055	ACAP1,WASL,TSG101, CBL,VPS37B,ARFGEF2, WIPF1,SH3KBP1,

				EHD1
Metabolic pathways	20	0.36	0.0117	NDUFB4,UQCRC1,NDUFB7, NDUFS7,NDUFB3,ACYP1, NDUFA2,NDUFA10,NDUFB5, SETD1A,NDUFS3,NDUFA9, UQCRC2,NDUFB10,CYC1, ALDH1A3,ASH1L,AK9, NDUFA6,PGM3
Epstein-Barr virus infection	6	0.71	0.0299	PSMC4,PSMD8,PSMD7, PSMC1,PSMD11,PSMD3

Table S9. STRING Network statistics for probe 7

number of nodes:
94
number of edges:
419
average node degree:
8.91
avg. local clustering coefficient:
0.598
expected number of edges:
193
PPI enrichment p-value:
< 1.0e-16

Table S10. Molecular Function (Gene Ontology) Analysis for probe 7

#term ID	term description	observed gene count	strength	false discovery rate	matching proteins in your network (labels)
GO:0031748	D1 dopamine receptor binding	3	1.8	0.0086	VPS35,PTPN11,SNX5

STK_Probe 3_C1F22-E

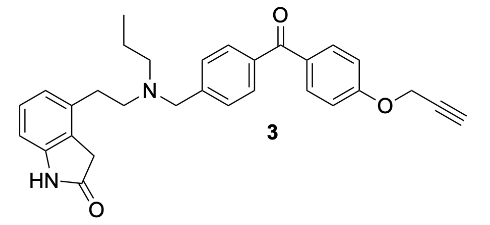

3

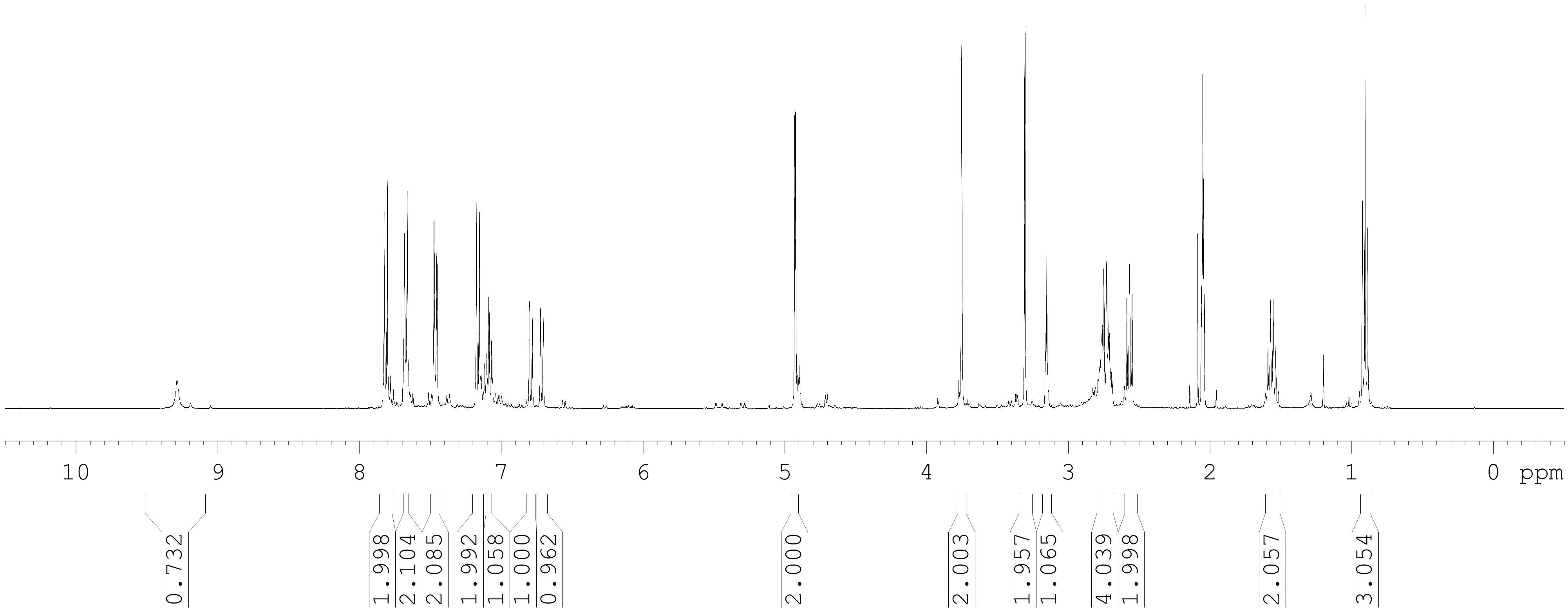

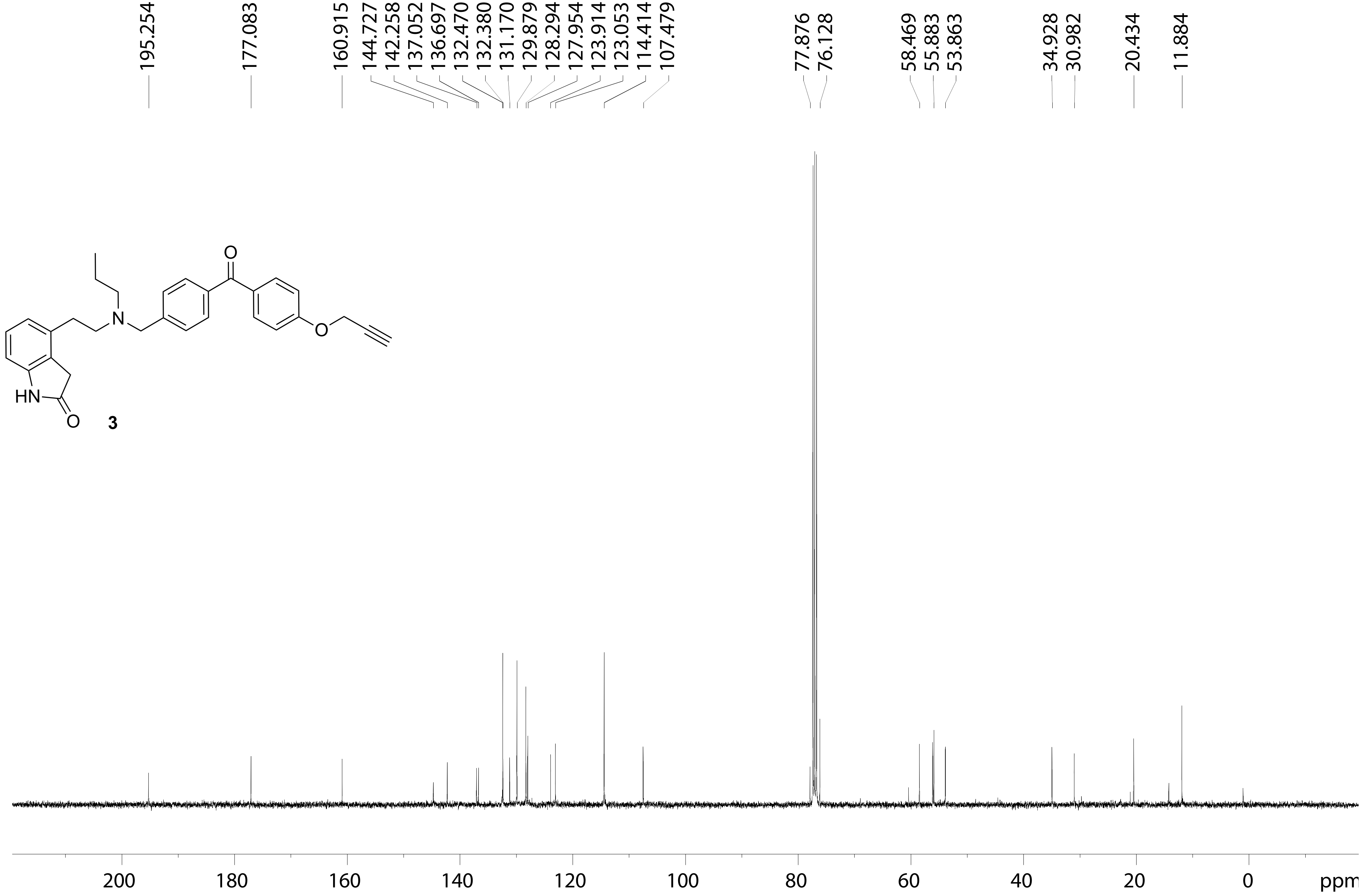

195.254
177.083
160.915
144.727
142.258
137.052
136.697
132.470
132.380
131.170
129.879
128.294
127.954
123.914
123.053
114.414
107.479
77.876
76.128
58.469
55.883
53.863
34.928
30.982
20.434
11.884
200
180
160
140
120
100
80
60
40
20
0
ppm

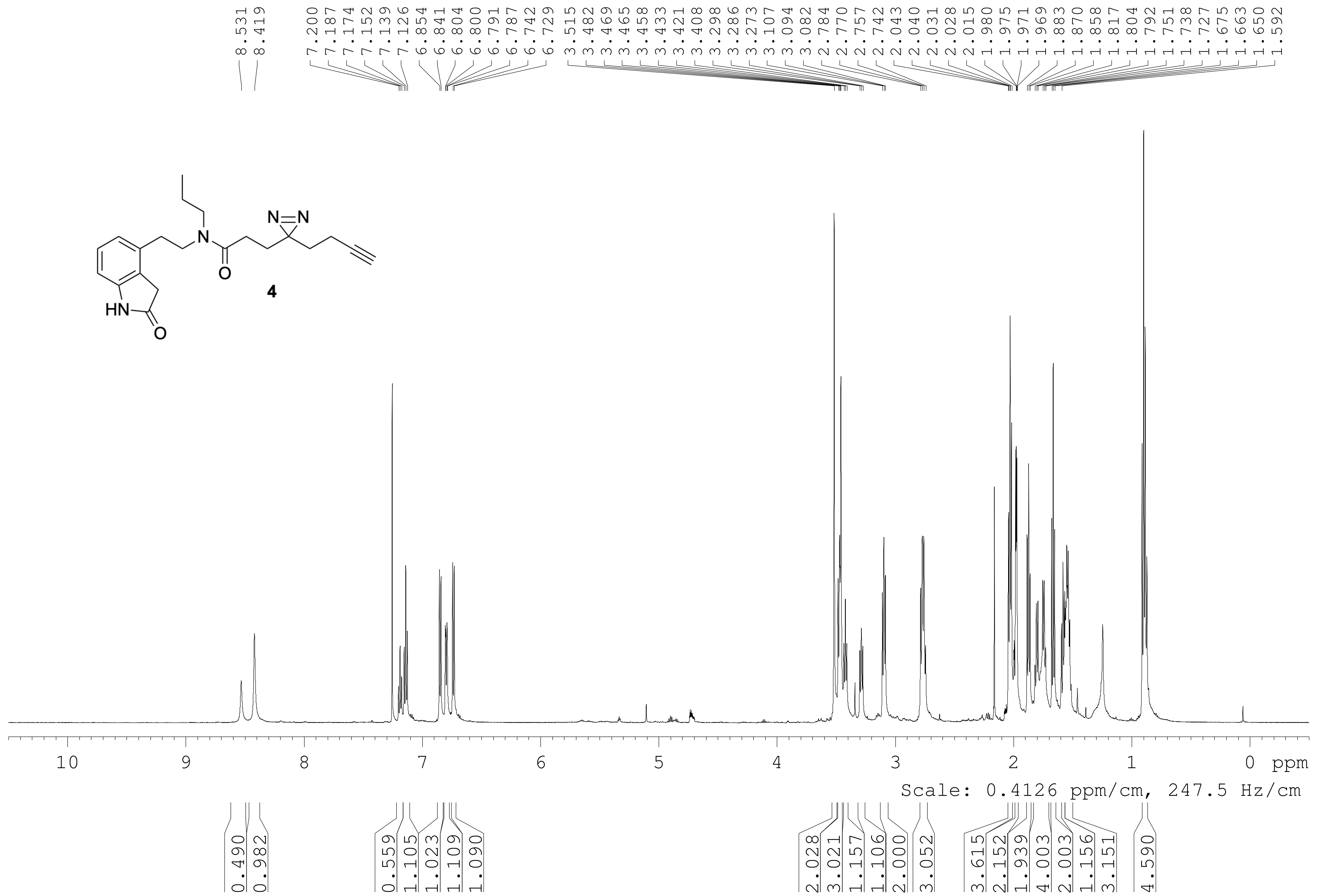
8.531
8.419
7.200
7.187
7.174
7.152
7.139
7.126
6.854
6.841
6.804
6.800
6.791
6.787
6.742
6.729
3.515
3.482
3.469
3.465
3.458
3.433
3.421
3.408
3.298
3.286
3.273
3.107
3.094
3.082
2.784
2.770
2.757
2.742
2.043
2.040
2.031
2.028
2.015
1.980
1.975
1.971
1.969
1.883
1.870
1.858
1.817
1.804
1.792
1.751
1.738
1.727
1.675
1.663
1.650
1.592
HN
O
N
O
N=N
4
10
9
8
7
6
5
4
3
2
1
0 ppm
Scale: 0.4126 ppm/cm, 247.5 Hz/cm
0.490
0.982
0.559
1.105
1.023
1.109
1.090
2.028
3.021
1.157
1.106
2.000
3.052
3.615
2.152
1.939
4.003
2.003
1.156
3.151
4.590

Probe 4_1H

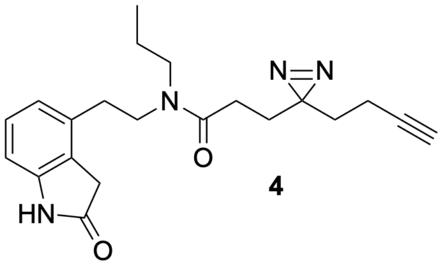

4

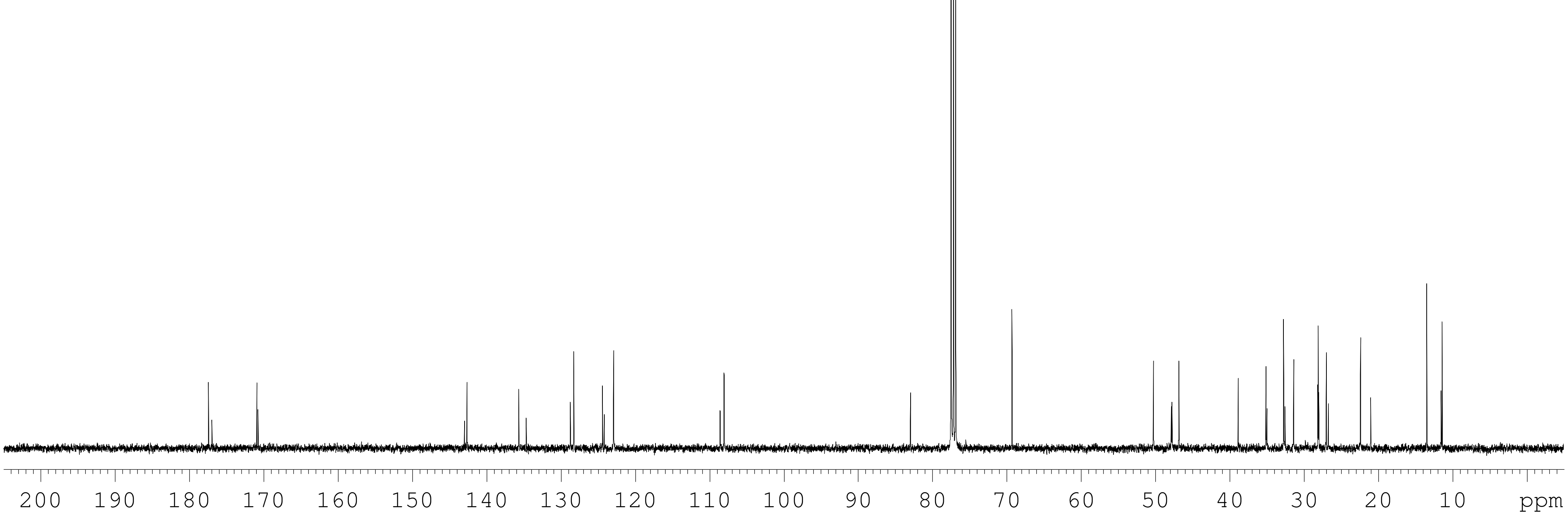
177.396
176.941
170.898
170.725
142.939
142.595
135.635
134.631
128.695
128.239
124.369
124.141
122.903
122.858
108.541
108.002
82.910
77.338
69.252
50.200
47.794
47.705
46.788
38.788
35.065
34.930
32.693
32.646
32.504
31.345
28.107
28.036
27.954
26.945
26.659
22.340
20.971
13.417
11.488
11.351
200 190 180 170 160 150 140 130 120 110 100 90 80 70 60 50 40 30 20 10 ppm

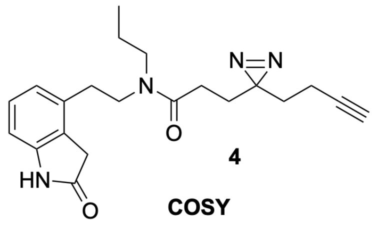

4

COSY

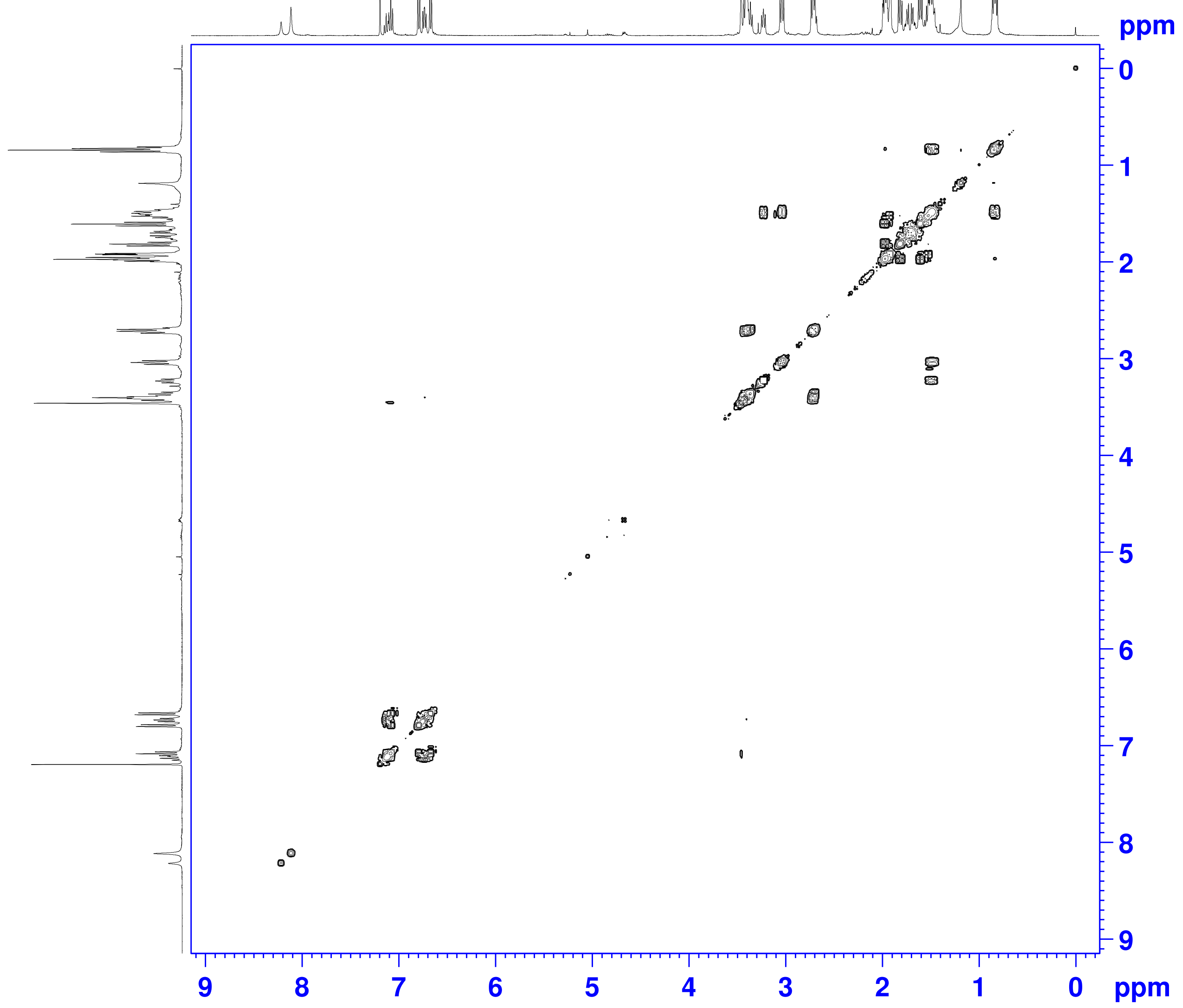

Current Data Parameters	
NAME	cmpd 4 cosy
EXPNO	4
PROCNO	1
F2 - Acquisition Parameters	
Date_	20220825
Time	18.10 h
INSTRUM	spect
PROBHD	Z116098_0203 (
PULPROG	cosygpppqf
TD	2048
SOLVENT	CDCl3
NS	1
DS	16
SWH	3759.398 Hz
FIDRES	3.671288 Hz
AQ	0.2723840 sec
RG	66.01
DW	133.000 usec
DE	6.50 usec
TE	298.0 K
D0	0.00000300 sec
D1	1.92463398 sec
D11	0.03000000 sec
D12	0.00002000 sec
D13	0.00000400 sec
D16	0.00020000 sec
IN0	0.00026600 sec
TDav	1
SFO1	400.1318173 MHz
NUC1	1H
P0	10.00 usec
P1	10.00 usec
P17	2500.00 usec
PLW1	16.20000076 W
PLW10	1.79999995 W
GPNAM[1]	SMSQ10.100
GPZ1	10.00 %
P16	1000.00 usec
F1 - Acquisition parameters	
TD	128
SFO1	400.1318 MHz
FIDRES	58.740601 Hz
SW	9.395 ppm
FnMODE	QF
F2 - Processing parameters	
SI	1024
SF	400.1300361 MHz
WDW	QSINE
SSB	0
LB	0 Hz
GB	0
PC	1.40
F1 - Processing parameters	
SI	1024
MC2	QF
SF	400.1300361 MHz
WDW	QSINE
SSB	0
LB	0 Hz
GB	0

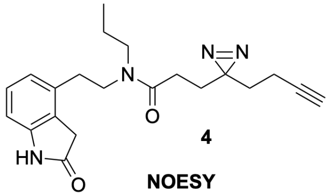

4

NOESY

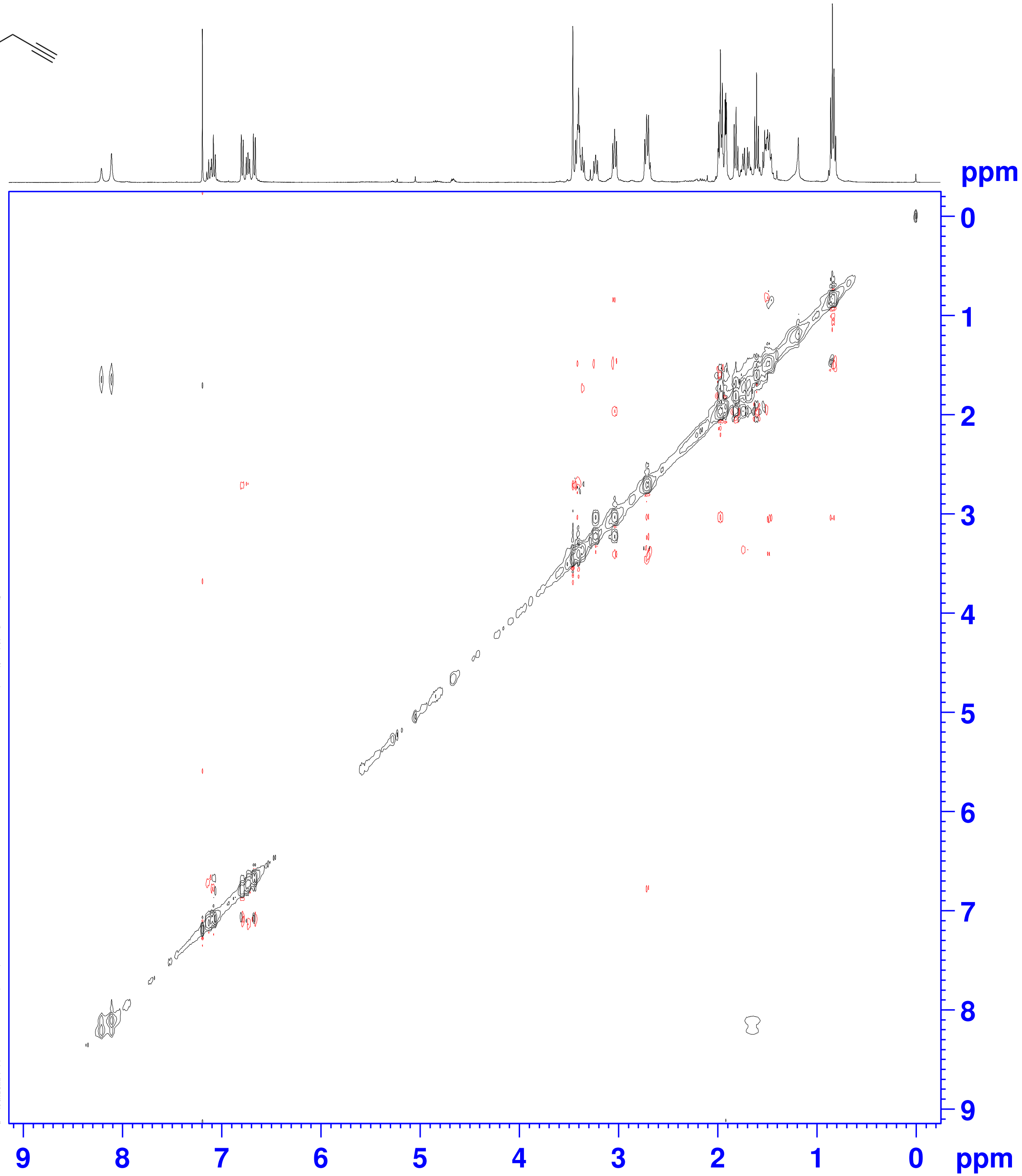

Current Data Parameters
NAME cmpd 4 noesy try 2
EXPNO 2
PROCNO 1

F2 - Acquisition Parameters
Date_ 20220825
Time 2.30 h
INSTRUM spect
PROBHD Z116098_0203 (
PULPROG noesygpphpp
TD 2048
SOLVENT CDCl3
NS 4
DS 32
SWH 3759.398 Hz
FIDRES 3.671288 Hz
AQ 0.2723840 sec
RG 66.01
DW 133.000 usec
DE 18.79 usec
TE 298.0 K
D0 0.00012027 sec
D1 1.98361599 sec
D8 0.30000001 sec
D11 0.03000000 sec
D12 0.00002000 sec
D16 0.00020000 sec
IN0 0.00026600 sec
TDav 1
SFO1 400.1318162 MHz
NUC1 1H
P1 10.00 usec
P2 20.00 usec
P17 2500.00 usec
PLW1 16.20000076 W
PLW10 1.79999995 W
GPNAM[1] SMSQ10.100
GPZ1 40.00 %
P16 1000.00 usec

F1 - Acquisition parameters
TD 256
SFO1 400.1318 MHz
FIDRES 29.370300 Hz
SW 9.395 ppm
FnMODE States-TPPI

F2 - Processing parameters
SI 1024
SF 400.1300361 MHz
WDW QSINE
SSB 2
LB 0 Hz
GB 0
PC 1.00

F1 - Processing parameters
SI 1024
MC2 States-TPPI
SF 400.1300361 MHz
WDW QSINE
SSB 2
LB 0 Hz
GB 0

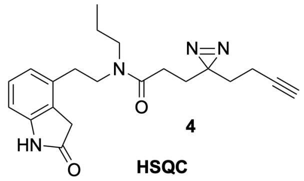

4

HSQC

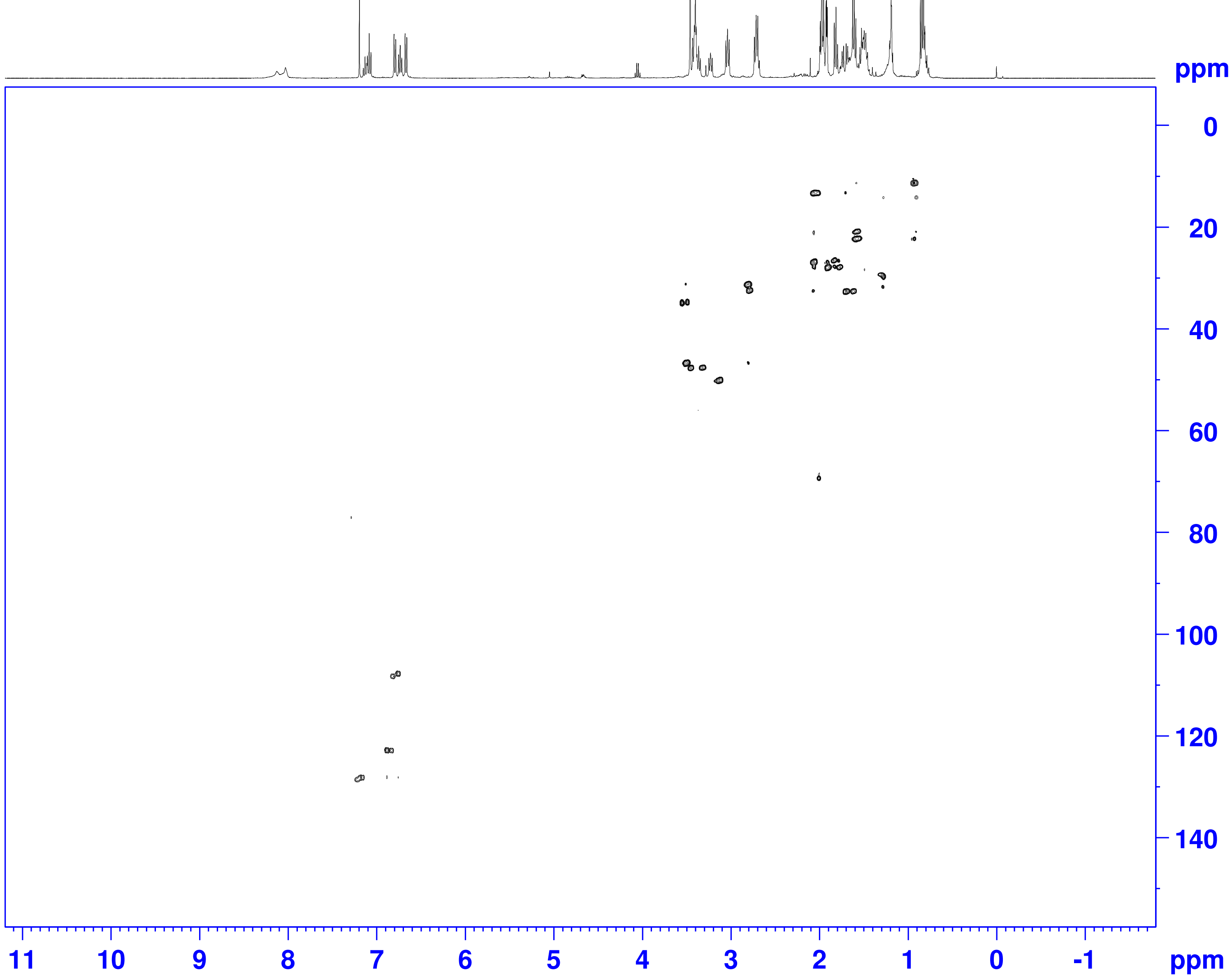


```
Current Data Parameters
NAME          cmpd 4 hsqc
EXPNO                   2
PROCNO                  1

F2 - Acquisition Parameters
Date_            20220831
Time                 1.12 h
INSTRUM             spect
PROBHD   Z116098_0203 (
PULPROG  hsqcedetgpsisp2.3
TD                   2048
SOLVENT             CDCl3
NS                      4
DS                     32
SWH              5197.505 Hz
FIDRES           5.075689 Hz
AQ              0.1970176 sec
RG                 208.09
DW                 96.200 usec
DE                  12.92 usec
TE                  298.0 K
CNST2         145.0000000
CNST17         -0.5000000
D0             0.00000300 sec
D1             1.50000000 sec
D4             0.00172414 sec
D11            0.03000000 sec
D16            0.00020000 sec
D21            0.00344800 sec
D24            0.00086200 sec
IN0            0.00003010 sec
TDav                    1
ZGOPTNS
SFO1          400.1318806 MHz
NUC1                   1H
P1                  10.00 usec
P2                  20.00 usec
PLW1          16.20000076 W
SFO2          100.6203145 MHz
NUC2                  13C
CPDPRG[2            garp4
P3                  10.00 usec
P14                500.00 usec
P24               2000.00 usec
P31               2119.00 usec
PCPD2               80.00 usec
PLW0                    0 W
PLW2          70.00000000 W
PLW12          1.09379995 W
SPNAM[3] Crp60,0.5,20.1
SPOAL3              0.500
SPOFFS3                 0 Hz
SPW3          10.69499969 W
SPNAM[7]     Crp60comp.4
SPOAL7              0.500
SPOFFS7                 0 Hz
SPW7          10.69499969 W
SPNAM[18   Crp60_xfilt.2
SPOAL18             0.500
SPOFFS18                0 Hz
SPW18          2.06040001 W
GPNAM[1]      SMSQ10.100
GPZ1                80.00 %
GPNAM[2]      SMSQ10.100
GPZ2                20.10 %
GPNAM[3]      SMSQ10.100
GPZ3                11.00 %
GPNAM[4]      SMSQ10.100
GPZ4                -5.00 %
P16               1000.00 usec
P19                600.00 usec

F1 - Acquisition parameters
TD                    256
SFO1             100.6203 MHz
FIDRES         129.775742 Hz
SW                165.089 ppm
FnMODE       Echo-Antiecho

F2 - Processing parameters
SI                   1024
SF            400.1300000 MHz
WDW                 QSINE
SSB                     2
LB                      0 Hz
GB                      0
PC                   1.40

F1 - Processing parameters
SI                   1024
MC2          echo-antiecho
SF            100.6127690 MHz
WDW                 QSINE
SSB                     2
LB                      0 Hz
GB                      0
```

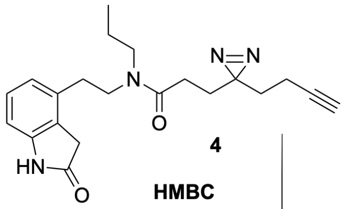

4

HMBC

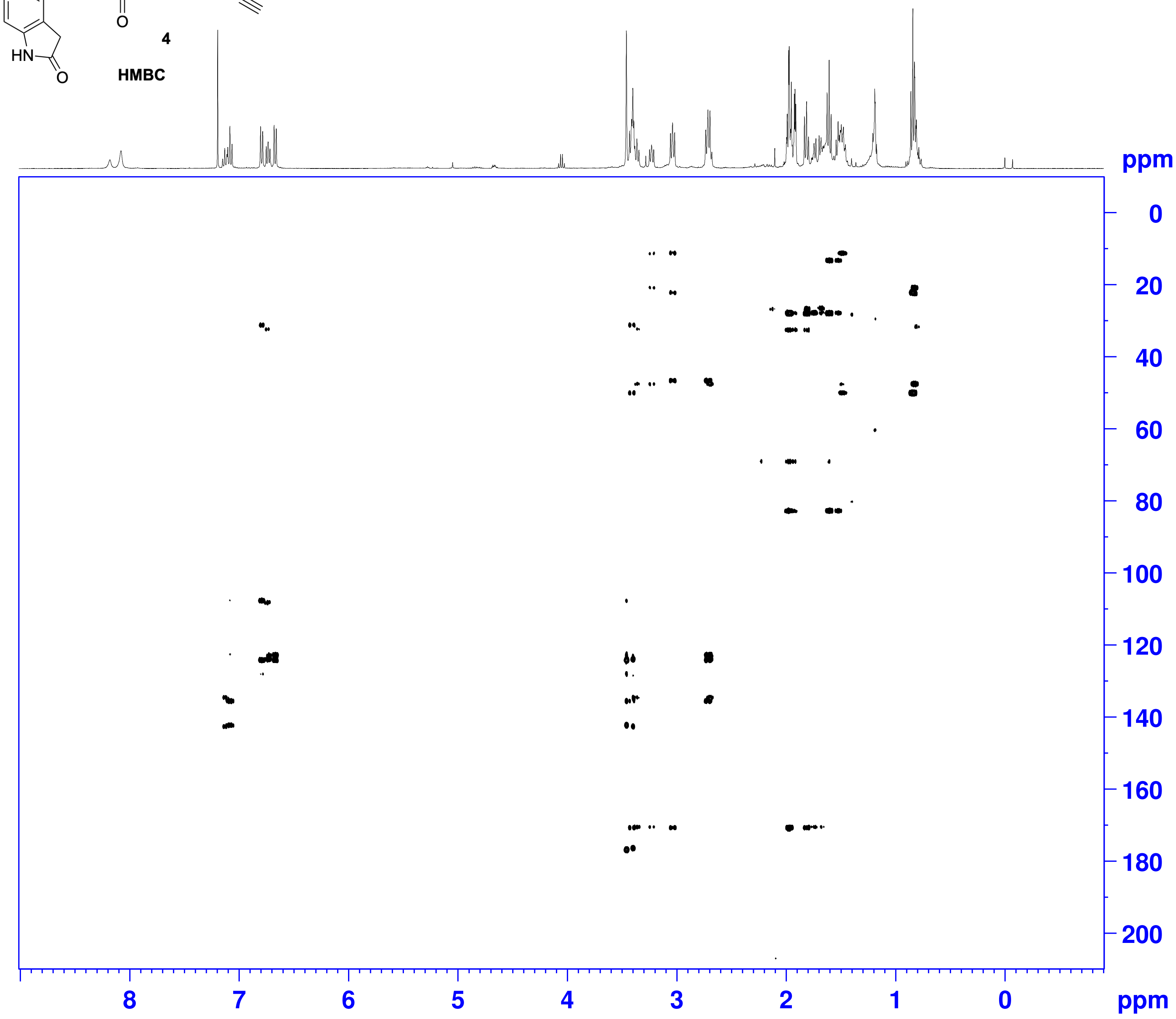


```
Current Data Parameters
NAME              cmpd 4 hmbc
EXPNO                       2
PROCNO                      1

F2 - Acquisition Parameters
Date_                20220831
Time                    18.54 h
INSTRUM                 spect
PROBHD      Z116098_0203 (
PULPROG        hmbcetgpl3nd
TD                       4096
SOLVENT                 CDCl3
NS                          4
DS                         16
SWH                  3968.254 Hz
FIDRES               1.937624 Hz
AQ                  0.5160960 sec
RG                     208.09
DW                    126.000 usec
DE                       6.50 usec
TE                      298.0 K
CNST6             120.0000000
CNST7             170.0000000
CNST13              8.0000000
D0                 0.00000300 sec
D1                 2.00000000 sec
D6                 0.06250000 sec
D16                0.00020000 sec
IN0                0.00002260 sec
TDav                        1
SFO1              400.1316582 MHz
NUC1                       1H
P1                      10.00 usec
P2                      20.00 usec
PLW1              16.20000076 W
SFO2              100.6228298 MHz
NUC2                      13C
P3                      10.00 usec
P24                   2000.00 usec
PLW2              70.00000000 W
SPNAM[7]         Crp60comp.4
SPOAL7                  0.500
SPOFFS7                     0 Hz
SPW7              10.69499969 W
GPNAM[1]          SMSQ10.100
GPZ1                    80.00 %
GPNAM[3]          SMSQ10.100
GPZ3                    14.00 %
GPNAM[4]          SMSQ10.100
GPZ4                    -8.00 %
GPNAM[5]          SMSQ10.100
GPZ5                    -4.00 %
GPNAM[6]          SMSQ10.100
GPZ6                    -2.00 %
P16                   1000.00 usec

F1 - Acquisition parameters
TD                        256
SFO1                 100.6228 MHz
FIDRES             172.842926 Hz
SW                    219.870 ppm
FnMODE          Echo-Antiecho

F2 - Processing parameters
SI                       4096
SF                400.1300359 MHz
WDW                     QSINE
SSB                         4
LB                          0 Hz
GB                          0
PC                       1.40

F1 - Processing parameters
SI                       1024
MC2              echo-antiecho
SF                100.6127690 MHz
WDW                     QSINE
SSB                         2
LB                          0 Hz
GB                          0
```

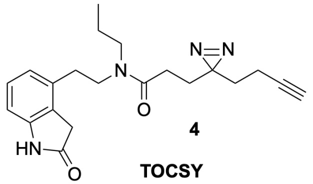

4

TOCSY

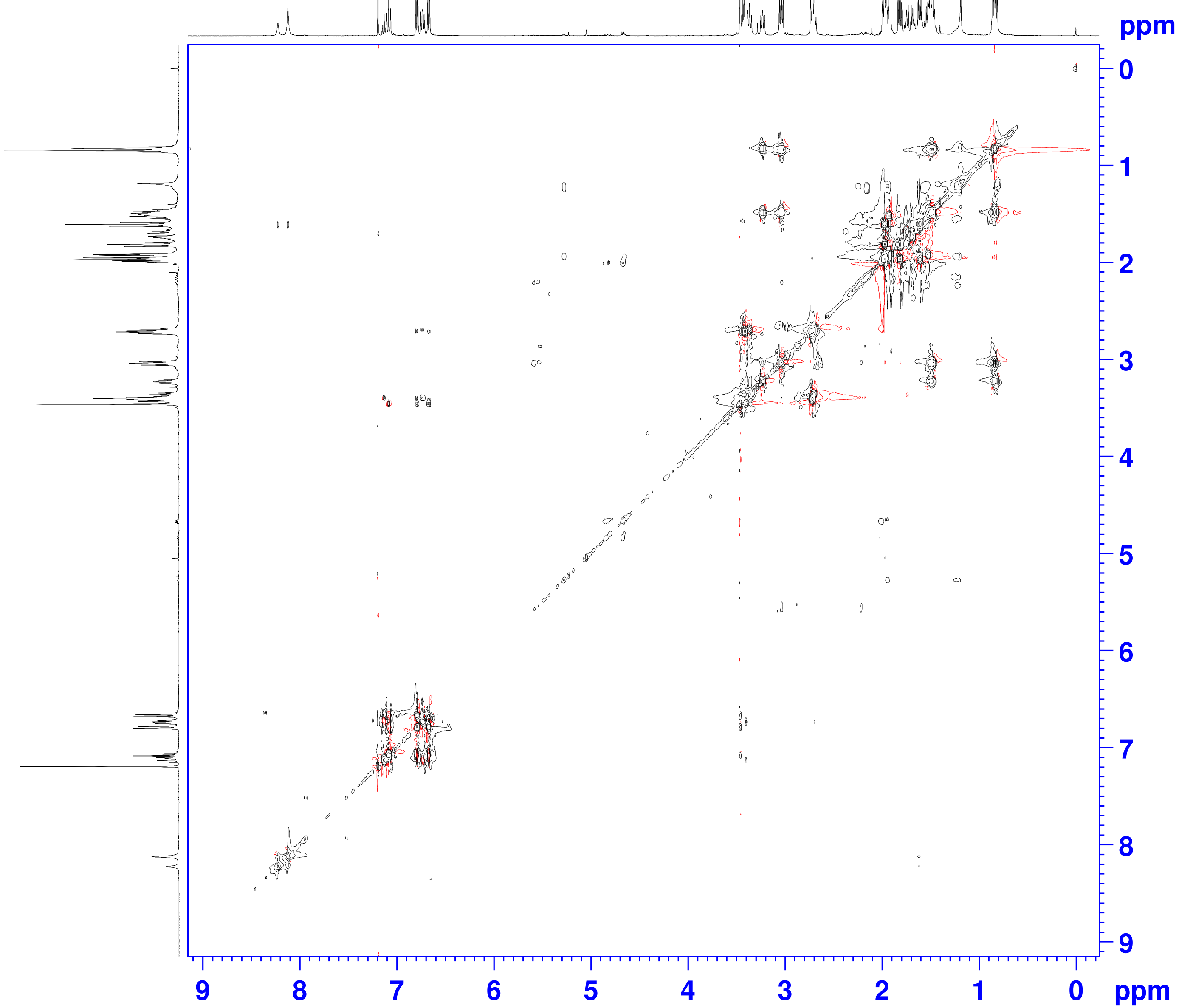


```
Current Data Parameters
NAME          cmpd 4 tocsy
EXPNO                    2
PROCNO                   1

F2 - Acquisition Parameters
Date_             20220827
Time                 11.38 h
INSTRUM              spect
PROBHD      Z116098_0203 (
PULPROG           mlevphpp
TD                    2048
SOLVENT              CDCl3
NS                       8
DS                      16
SWH               3759.398 Hz
FIDRES            3.671288 Hz
AQ               0.2723840 sec
RG                   66.01
DW                 133.000 usec
DE                    6.50 usec
TE                   298.0 K
D0              0.00012263 sec
D1              1.98361599 sec
D9              0.08000000 sec
D11             0.03000000 sec
D12             0.00002000 sec
IN0             0.00026600 sec
L1                      36
TDav                     1
SFO1           400.1318190 MHz
NUC1                    1H
P1                   10.00 usec
P5                   20.01 usec
P6                   30.00 usec
P7                   60.00 usec
P17                2500.00 usec
PLW1           16.20000076 W
PLW10           1.79999995 W

F1 - Acquisition parameters
TD                     256
SFO1              400.1318 MHz
FIDRES           29.370300 Hz
SW                   9.395 ppm
FnMODE         States-TPPI

F2 - Processing parameters
SI                    1024
SF             400.1300361 MHz
WDW                  QSINE
SSB                      2
LB                       0 Hz
GB                       0
PC                    1.40

F1 - Processing parameters
SI                    1024
MC2            States-TPPI
SF             400.1300361 MHz
WDW                  QSINE
SSB                      2
LB                       0 Hz
GB                       0
```

Probe 4 2D NMR Assignments

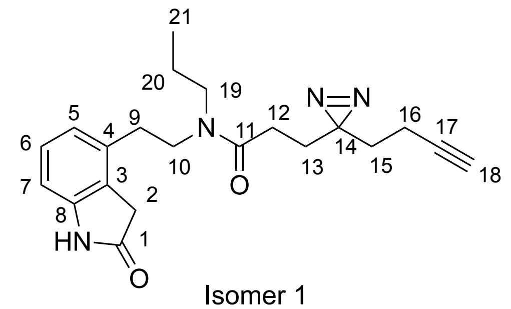

Isomer 1

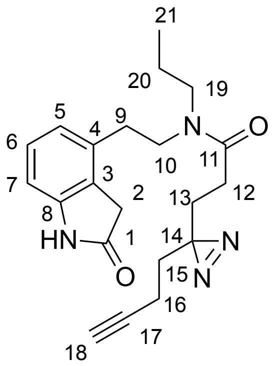

Isomer 2

Isomer 1

	Cδ	HSQC	Hδ	multiplicity	J-value	COSY	HMBC
C1	177.405						H1
C2	35.07	H1	3.51	s		H5, H6	H2, H3, H4, H13
C3	124.37						H1, H2, H4
C4	135.63						H1, H3
C5	122.9	H2	6.847	d	7.8 Hz	H3	H1, H2
C6	128.24	H3	7.1388	t	7.7 Hz	H2, H4	H1
C7	108	H4	6.736	d	7.7 Hz	H3	H1, H2
C8	142.59						H1, H3
C9	31.34	H5	2.77	m		H1,H7	H3
C10	46.79	H6	3.095	t	7.6 Hz	H14	H13
C11	170.9						H6
C12	28.04	H10	2.028	t	7.3 Hz	H11	H2
C13	32.69	H11	1.663	t	7.4 Hz	H10	H3
C14	13.42						H11
C15	38.79	H12	1.87	t	7.1 Hz	H11, H13	H3
C16	11.35	H13	1.975	m		H12, H14	H1
C17	82.91						H8
C18	69.25	H14	1.805	m		H13	H6, H14
C19	50.2	H7	3.469	t	7.8 Hz	H1, H5	
C20	22.34	H8	1.58	t	7.3 Hz	H9	H6
C21	26.95	H9	0.898	t	7.4 Hz	H8	H2

Isomer 2

	Cδ	HSQC	Hδ	multiplicity	J-value	COSY	HMBC
C1	176.94						H1
C2	34.835	H1	3.46	s		H5	H2, H3, H4
C3	124.4						H1
C4	134.63						H1
C5	122.86	H2	6.8	d	1.9 Hz	H3	H1, H4
C6	128.7	H3	7.187	t	7.7 Hz	H2, H4	H1
C7	108.54	H4	6.79	d	1.9 Hz	H3	H1, H2
C8	142.94						
C9	32.67	H5	2.695	m		H1, H6	H2, H4
C10	34.93	H6	3.286	t	7.7 Hz	H5, H11	H8, H14
C11	170.72						H14
C12	28.11	H10	2.031	t	7.5 Hz	H11	H8, H12
C13	32.5	H11	1.73	m		H6, H10	
C14	13.42						H12
C15	47.79	H12	1.58	m		H13	H5, H6, H9,
C16	11.49	H13	2.03	m		H12, H14	H11
C17	77.34						H13
C18	69.25	H14	1.73	m		H13	
C19	47.71	H7	3.421	t	7.4 Hz	H8	H11
C20	20.97	H8	1.54	m		H7, H9	H9, H13
C21	27.95	H9	0.883	t	7.6 Hz	H8	H7, H11

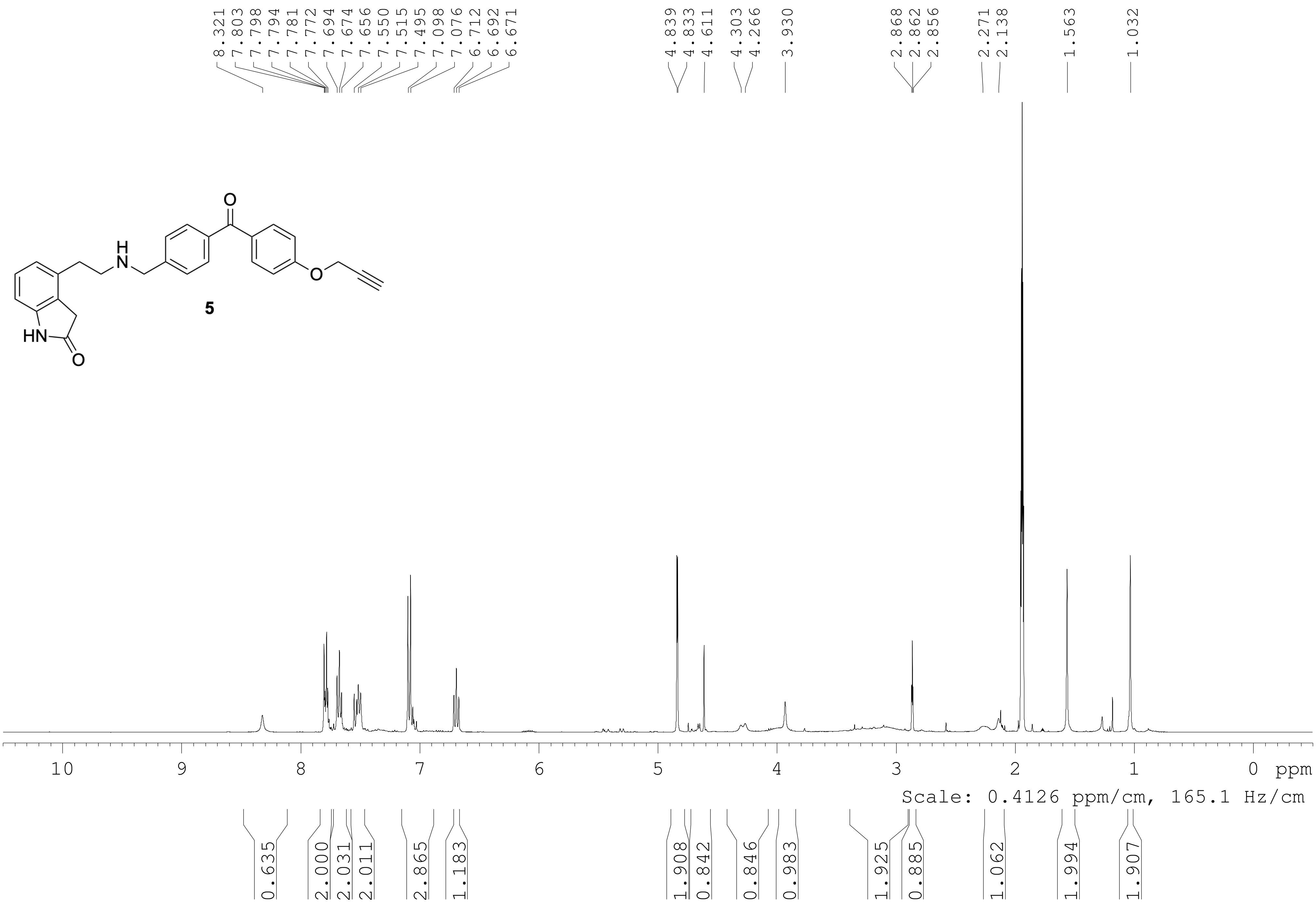

8.321
7.803
7.798
7.794
7.781
7.772
7.694
7.674
7.656
7.550
7.515
7.495
7.098
7.076
6.712
6.692
6.671
4.839
4.833
4.611
4.303
4.266
3.930
2.868
2.862
2.856
2.271
2.138
1.563
1.032
5
10
9
8
7
6
5
4
3
2
1
0 ppm
Scale: 0.4126 ppm/cm, 165.1 Hz/cm
0.635
2.000
2.031
2.011
2.865
1.183
1.908
0.842
0.846
0.983
1.925
0.885
1.062
1.994
1.907

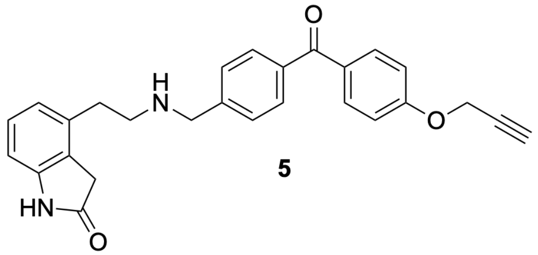

5

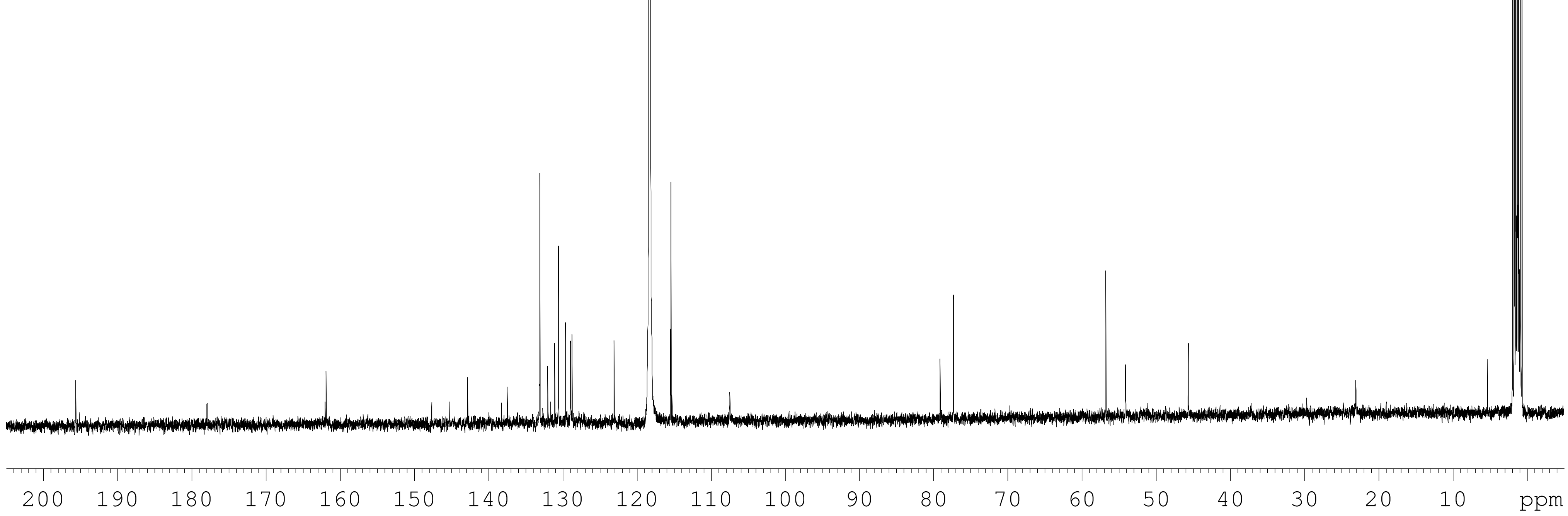
195.613
177.914
161.846
147.609
145.257
142.792
138.212
137.430
133.082
133.033
131.976
131.604
131.039
130.544
129.555
128.891
128.696
123.034
115.351
115.214
107.427
79.084
77.252
56.724
54.076
45.606
23.032
200 190 180 170 160 150 140 130 120 110 100 90 80 70 60 50 40 30 20 10 ppm

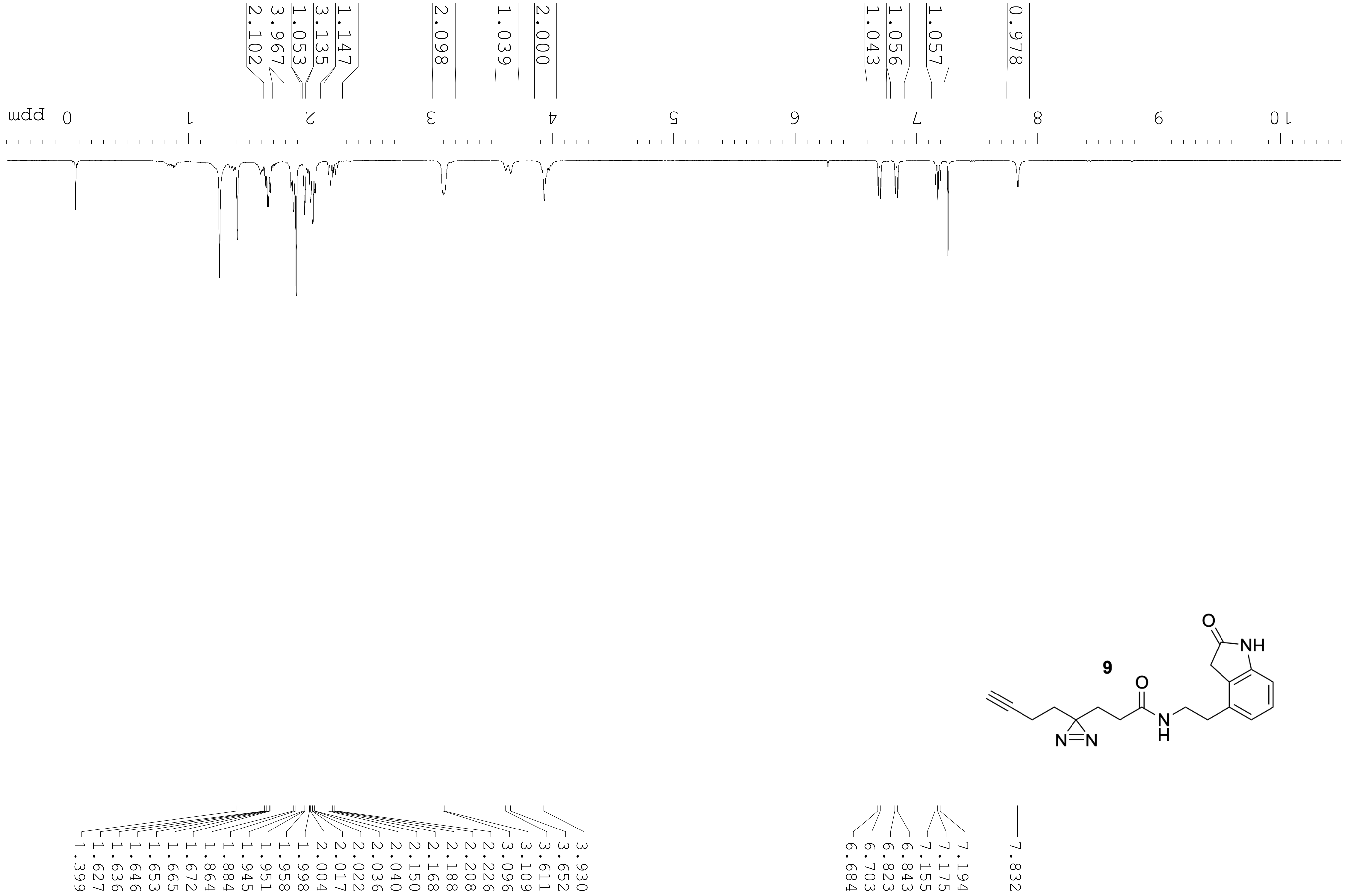
ppm
0
1
2
3
4
5
6
7
8
9
10
2.102
3.967
1.053
3.135
1.147
2.098
1.039
2.000
1.043
1.056
1.057
0.978
1.399
1.627
1.636
1.646
1.653
1.665
1.672
1.864
1.884
1.945
1.951
1.958
1.998
2.004
2.017
2.022
2.036
2.040
2.150
2.168
2.188
2.208
2.226
3.096
3.109
3.611
3.652
3.930
6.684
6.703
6.823
6.843
7.155
7.175
7.194
7.832
O
NH
N
H
N
N

9

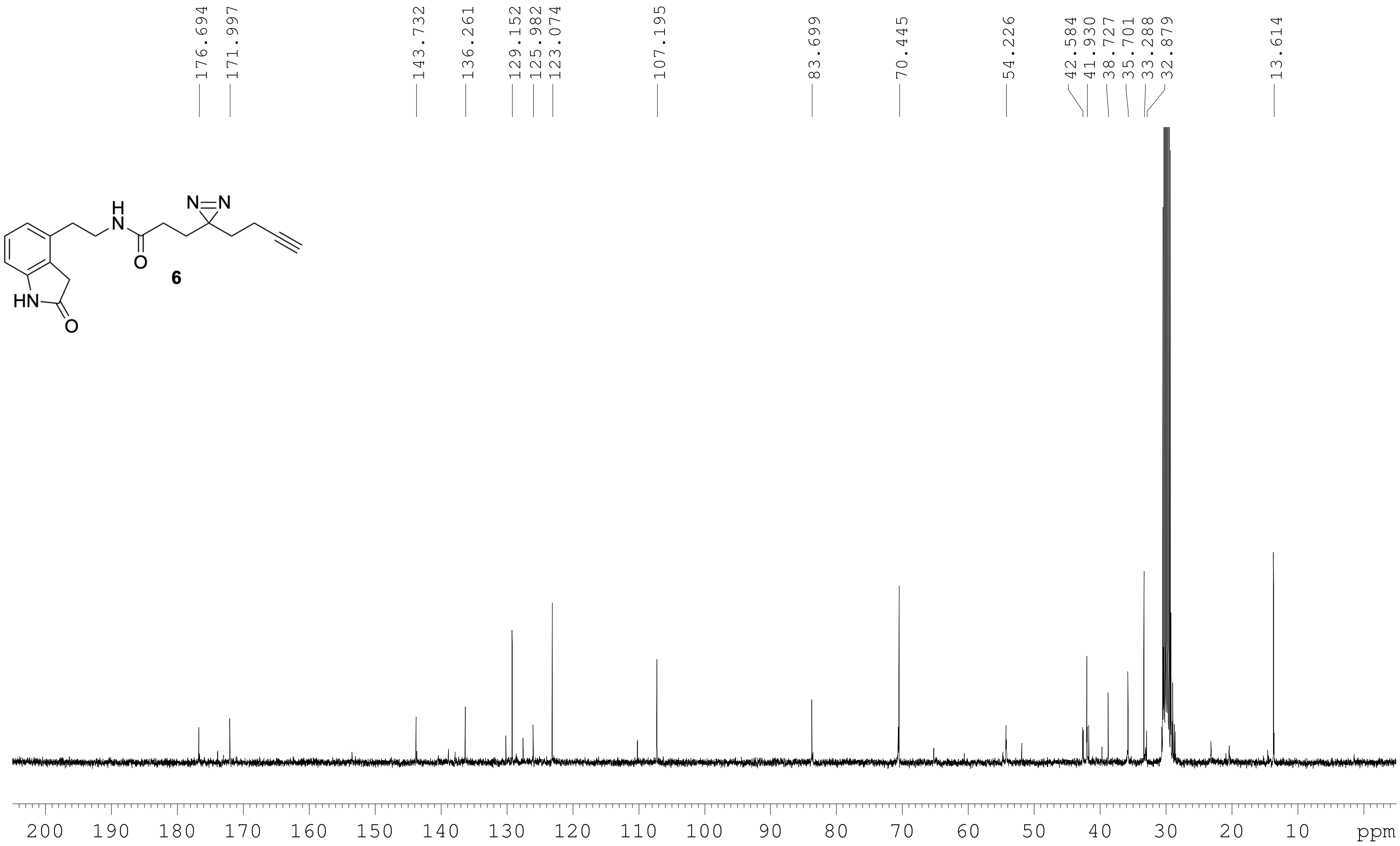
176.694
171.997
143.732
136.261
129.152
125.982
123.074
107.195
83.699
70.445
54.226
42.584
41.930
38.727
35.701
33.288
32.879
13.614
6
200 190 180 170 160 150 140 130 120 110 100 90 80 70 60 50 40 30 20 10 ppm

Benzophenone Pramipexole

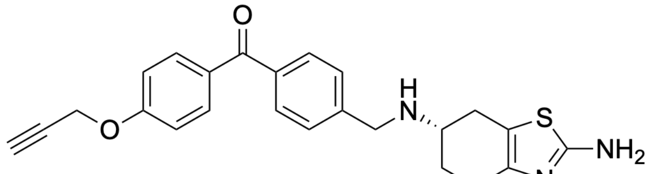

7

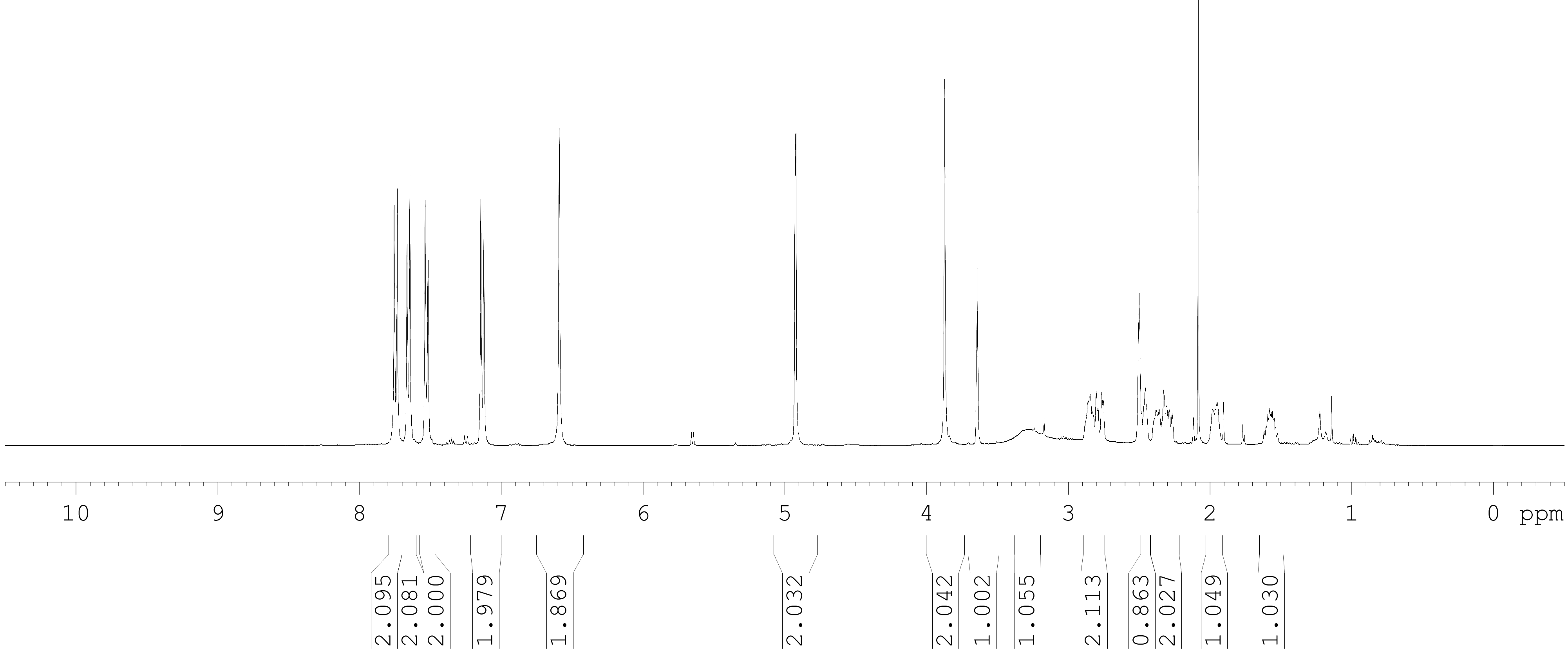

Benzophenone Pramipexole

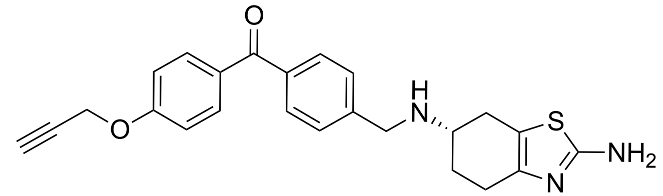

7

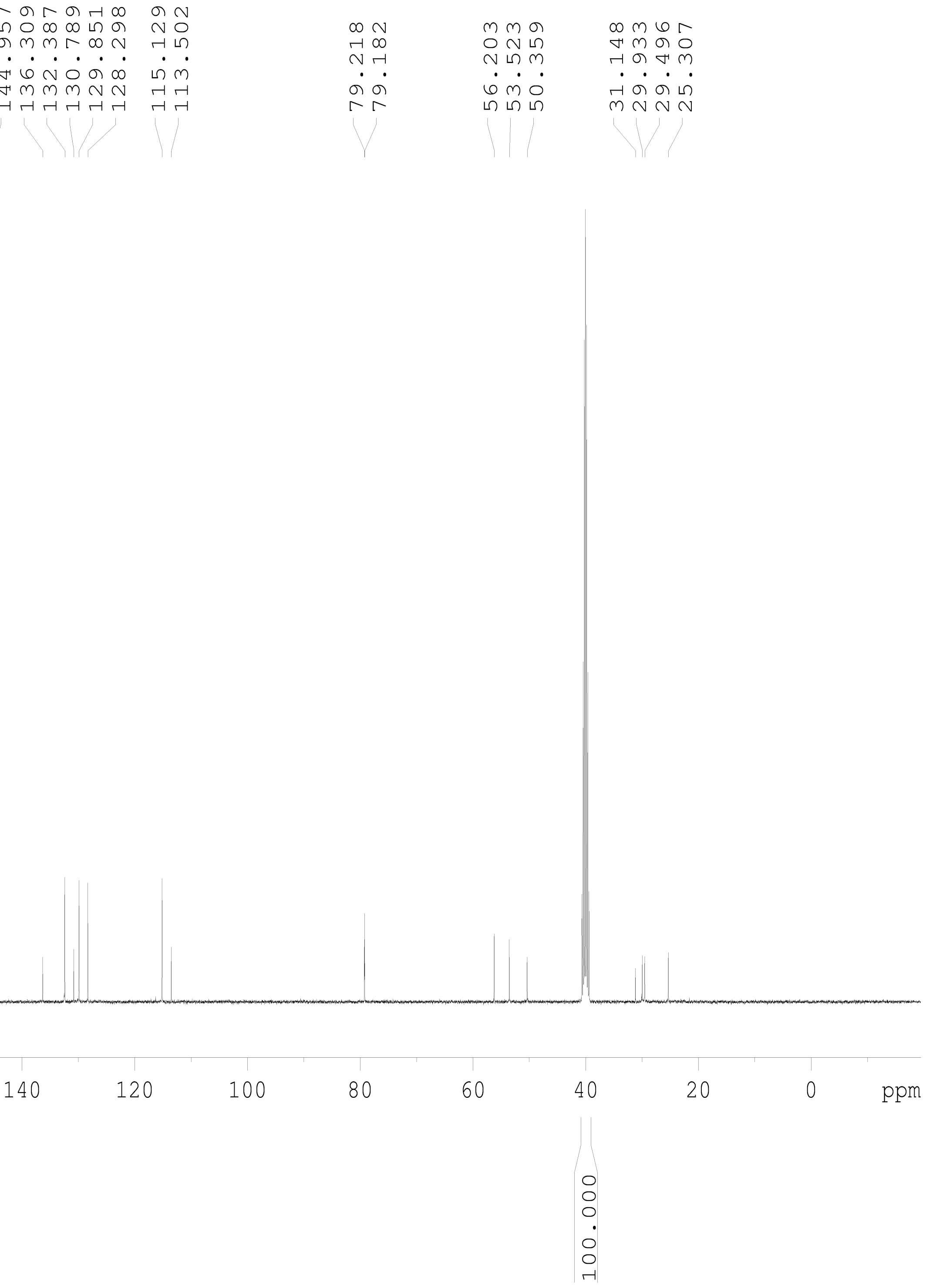

Pramipexole amide diazirine

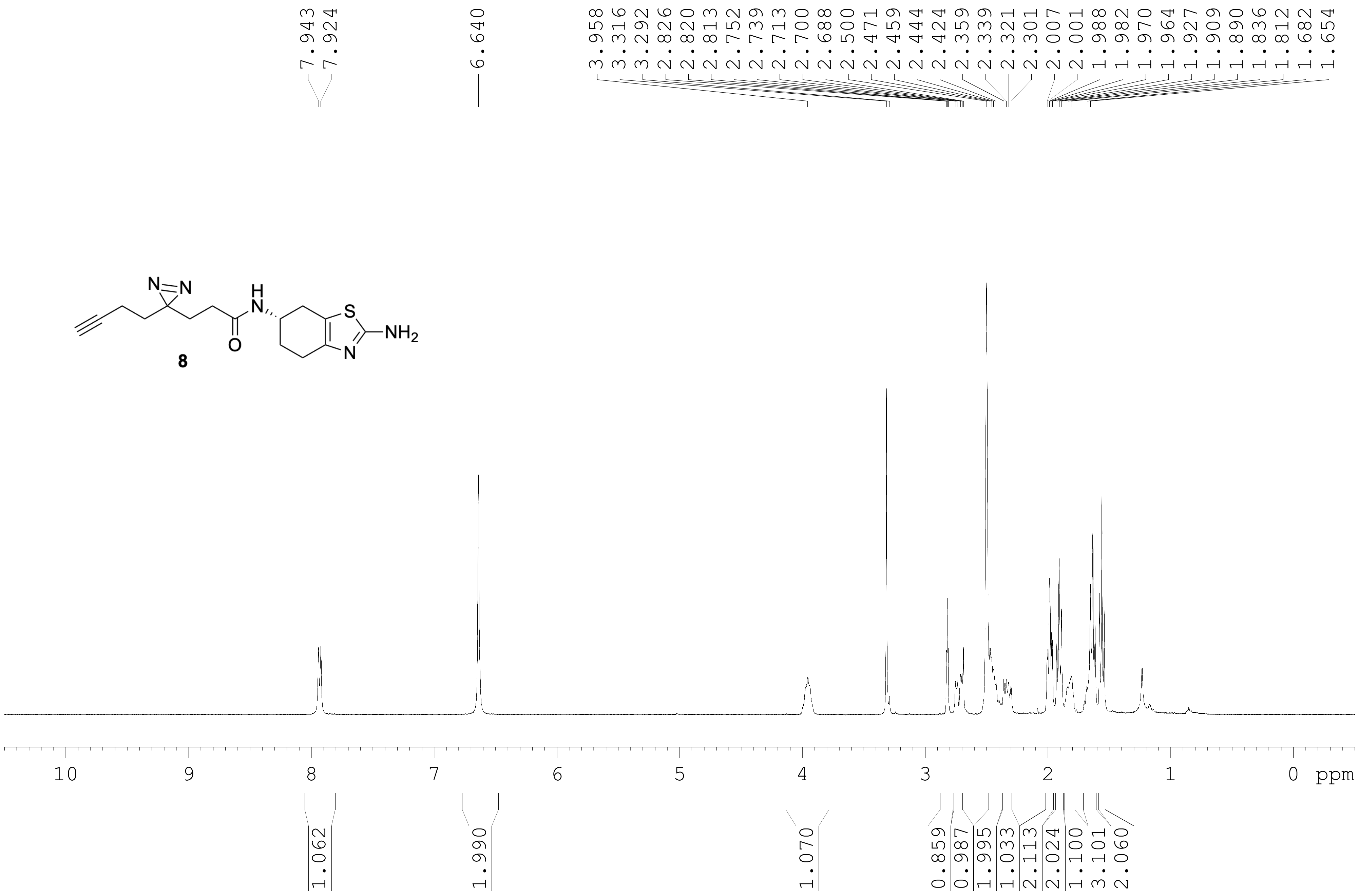

8

Diazirine Pramipexole

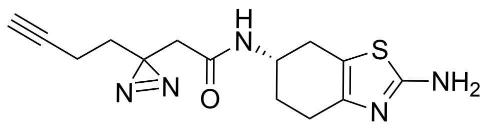

8

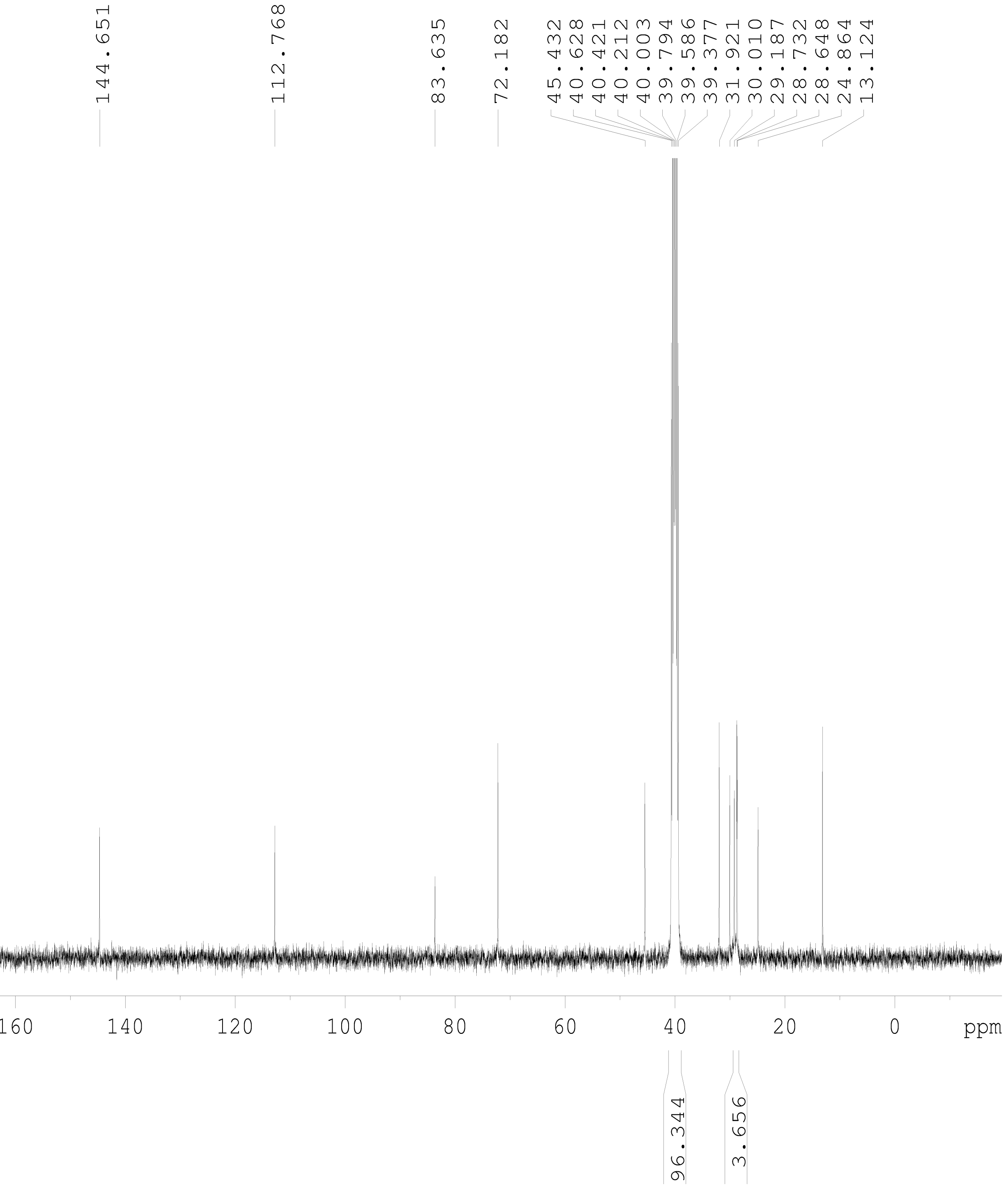

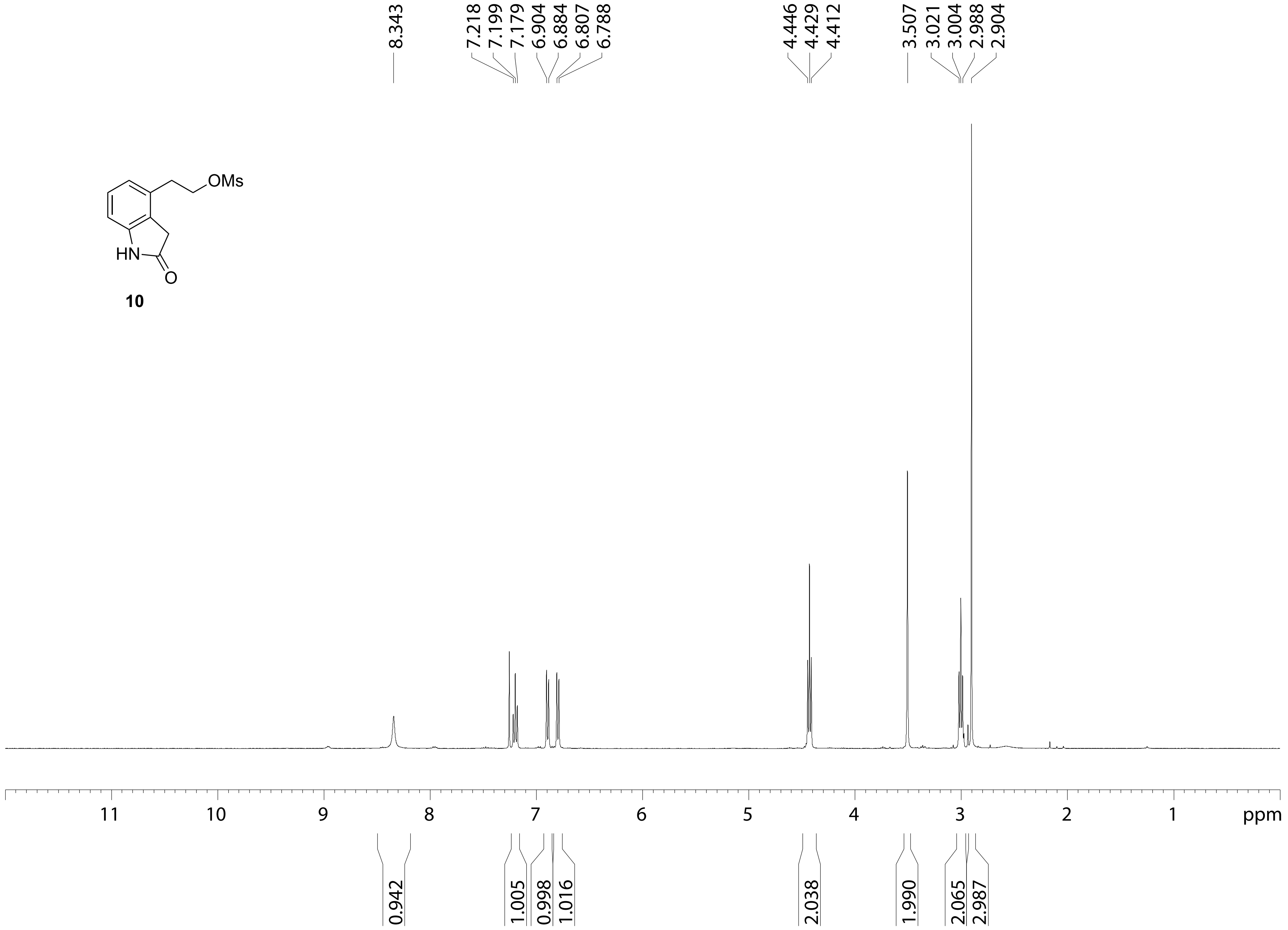
OMs
HN
O
10
8.343
7.218
7.199
7.179
6.904
6.884
6.807
6.788
4.446
4.429
4.412
3.507
3.021
3.004
2.988
2.904
11
10
9
8
7
6
5
4
3
2
1
ppm
0.942
1.005
0.998
1.016
2.038
1.990
2.065
2.987

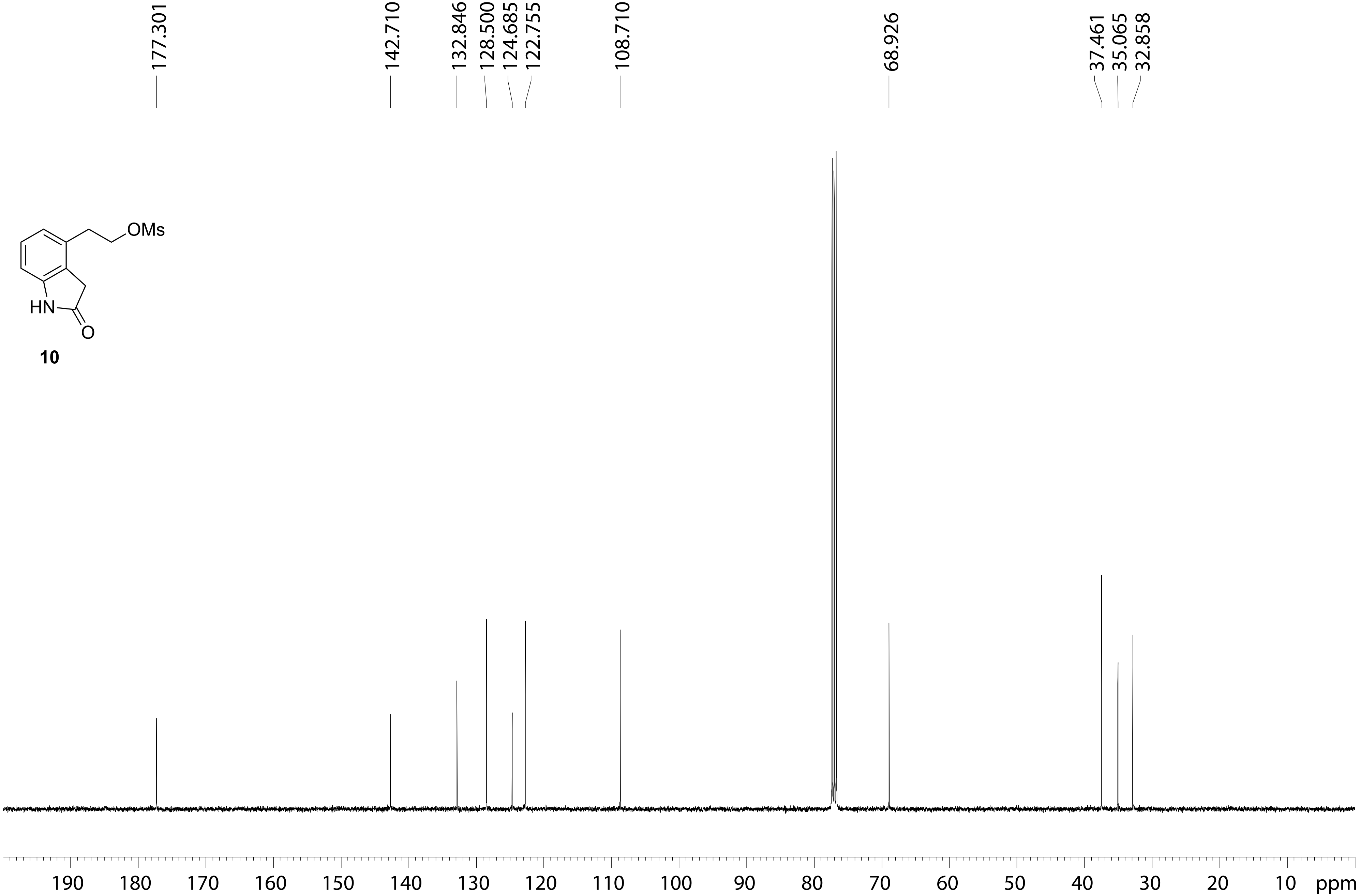
OMs
HN
O
10
177.301
142.710
132.846
128.500
124.685
122.755
108.710
68.926
37.461
35.065
32.858
190 180 170 160 150 140 130 120 110 100 90 80 70 60 50 40 30 20 10 ppm

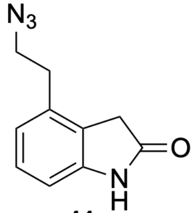

11

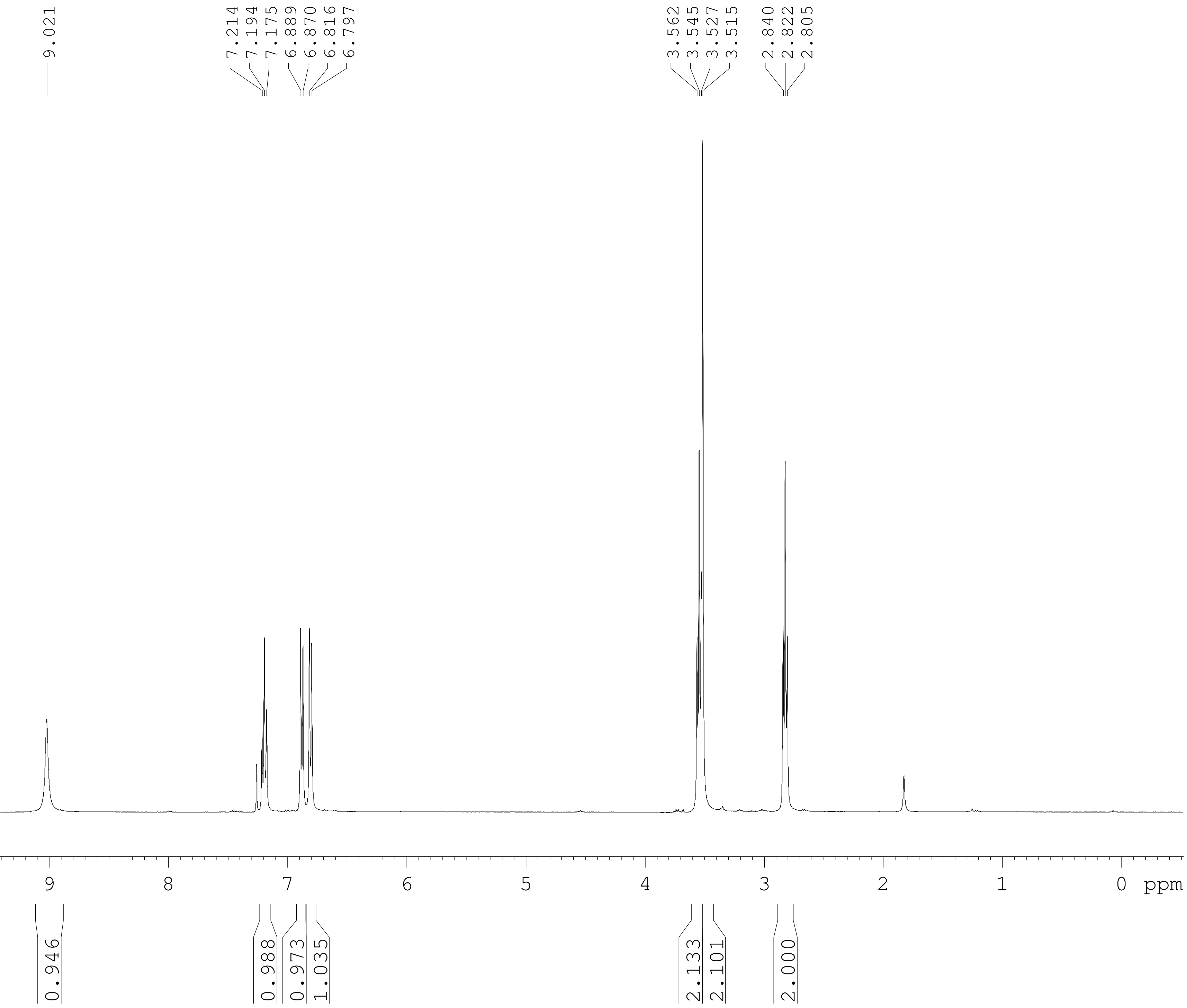
9.021
7.214
7.194
7.175
6.889
6.870
6.816
6.797
3.562
3.545
3.527
3.515
2.840
2.822
2.805
10
9
8
7
6
5
4
3
2
1
0
ppm
0.946
0.988
0.973
1.035
2.133
2.101
2.000

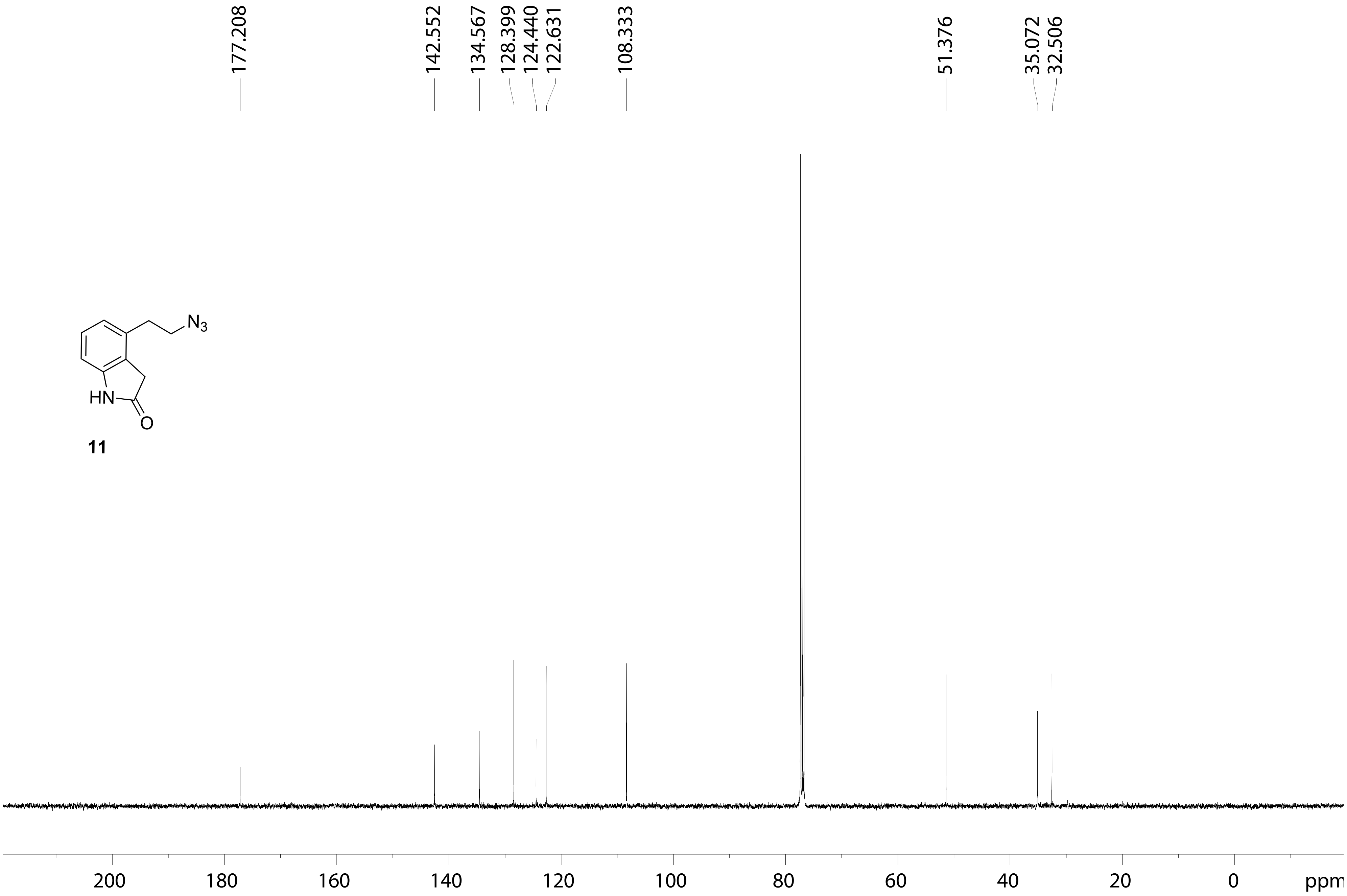
177.208
142.552
134.567
128.399
124.440
122.631
108.333
51.376
35.072
32.506
N3
HN
O
11
200
180
160
140
120
100
80
60
40
20
0
ppm

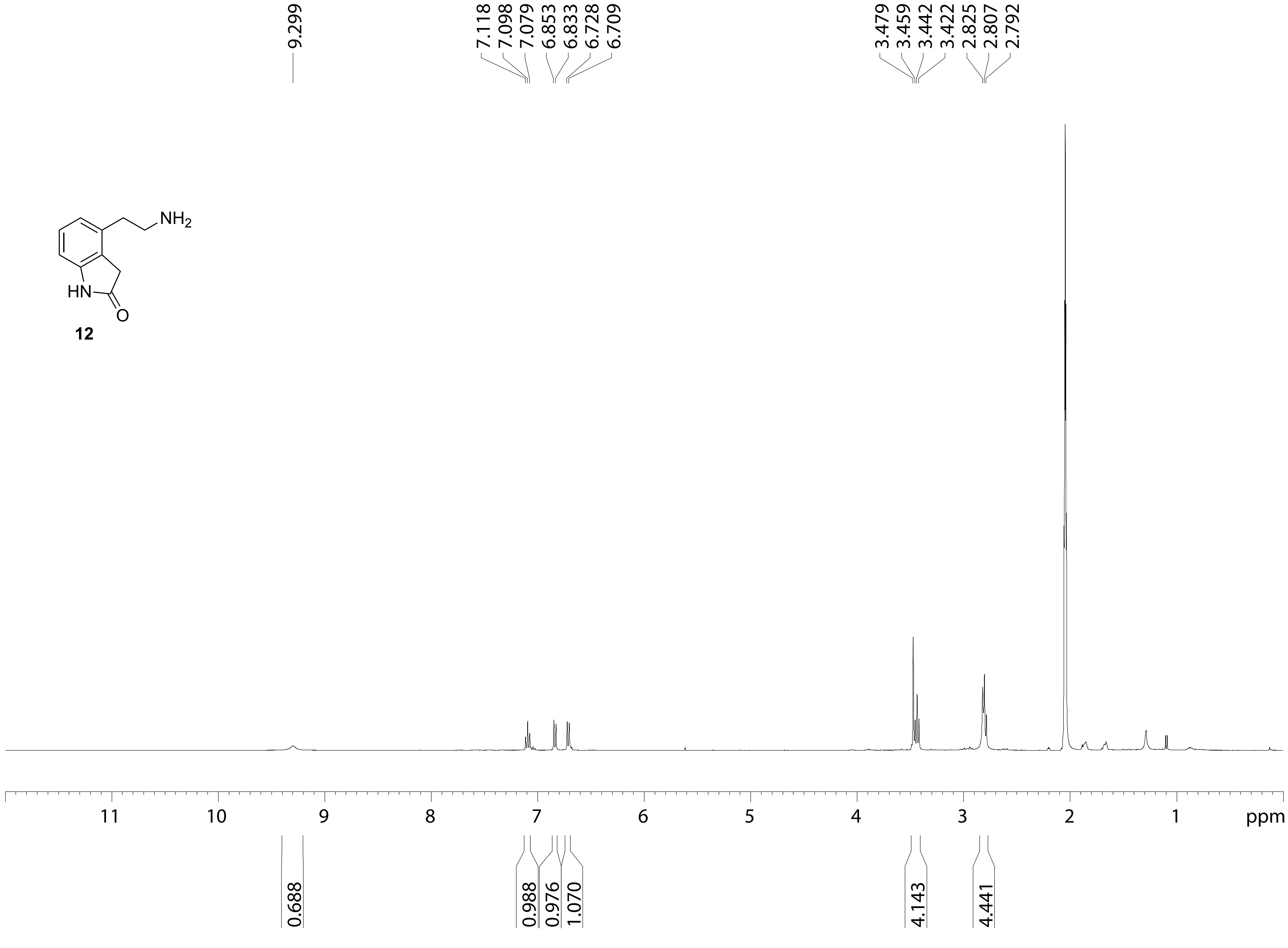
9.299
7.118
7.098
7.079
6.853
6.833
6.728
6.709
3.479
3.459
3.442
3.422
2.825
2.807
2.792
NH2
HN
O
12
11
10
9
8
7
6
5
4
3
2
1
ppm
0.688
0.988
0.976
1.070
4.143
4.441

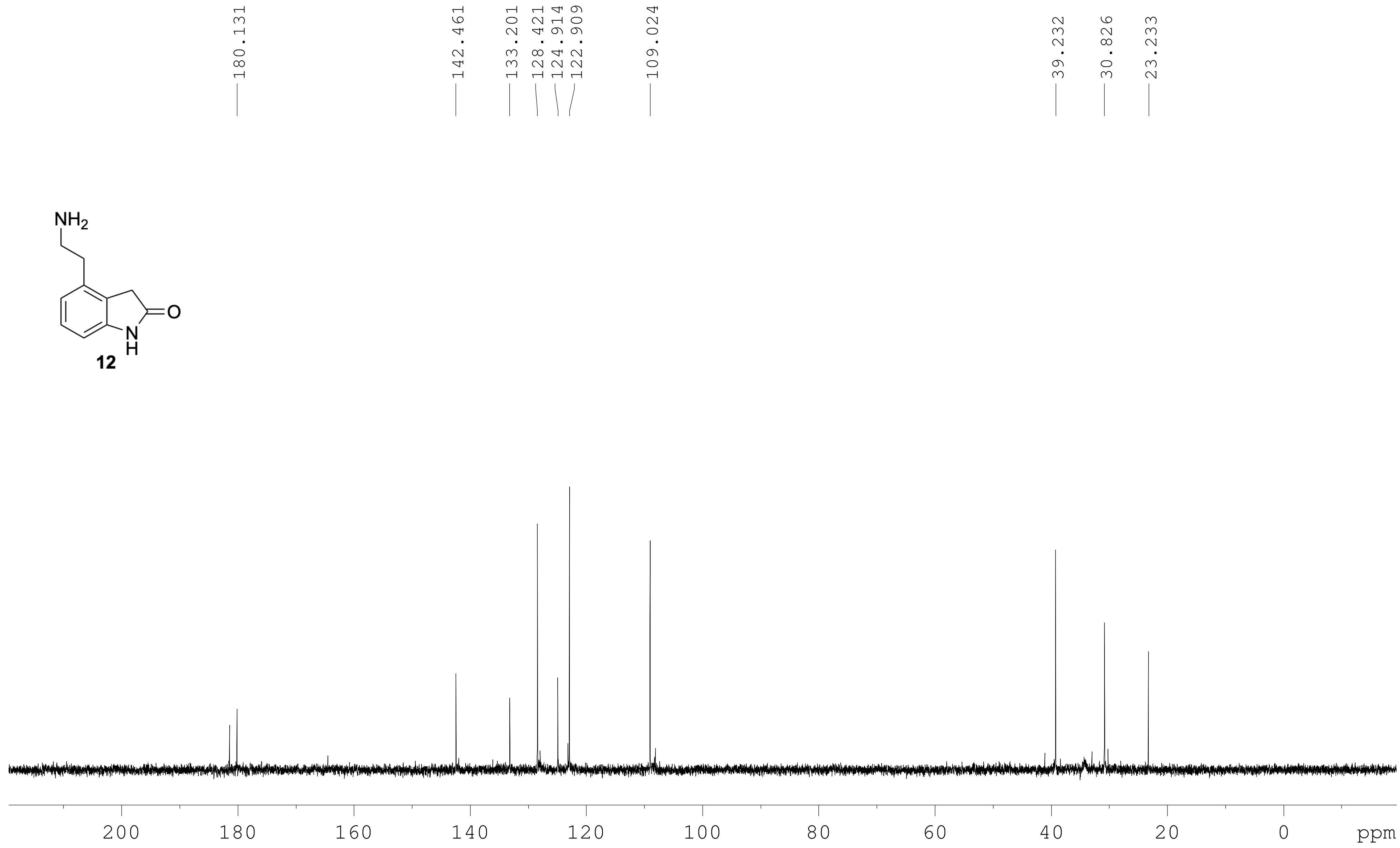
180.131
142.461
133.201
128.421
124.914
122.909
109.024
39.232
30.826
23.233
NH2
O
N
H
12
200
180
160
140
120
100
80
60
40
20
0
ppm
